# Automatic structure classification of small proteins using random forest

**DOI:** 10.1186/1471-2105-11-364

**Published:** 2010-07-01

**Authors:** Pooja Jain, Jonathan D Hirst

**Affiliations:** 1 School of Chemistry, The University of Nottingham, University Park, Nottingham, NG7 2RD, UK

## Abstract

****Background**:**

Random forest, an ensemble based supervised machine learning algorithm, is used to predict the SCOP structural classification for a target structure, based on the similarity of its structural descriptors to those of a template structure with an equal number of secondary structure elements (SSEs). An initial assessment of random forest is carried out for domains consisting of three SSEs. The usability of random forest in classifying larger domains is demonstrated by applying it to domains consisting of four, five and six SSEs.

****Result**s:**

Random forest, trained on SCOP version 1.69, achieves a predictive accuracy of up to 94% on an independent and non-overlapping test set derived from SCOP version 1.73. For classification to the SCOP *Class, Fold, Super-family *or *Family *levels, the predictive quality of the model in terms of Matthew's correlation coefficient (MCC) ranged from 0.61 to 0.83. As the number of constituent SSEs increases the MCC for classification to different structural levels decreases.

**Conclusions:**

The utility of random forest in classifying domains from the place-holder classes of SCOP to the true *Class, Fold, Super-family *or *Family *levels is demonstrated. Issues such as introduction of a new structural level in SCOP and the merger of singleton levels can also be addressed using random forest. A real-world scenario is mimicked by predicting the classification for those protein structures from the PDB, which are yet to be assigned to the SCOP classification hierarchy.

## Background

In the foreseeable future, current practices of cataloging protein structures will need to cope with a rapid increase in the number of unclassified structures. As of February 2010, approximately 64% of the 58,824 proteins deposited in the Protein Data Bank (PDB) [1] are structurally classified. The number of structurally resolved proteins is expected to continue to rise; about 1150 structures have been deposited in the PDB within the first two months of 2010. Moreover, about 29,000 of 40,000 representative proteins selected by the world-wide protein structure initiative (PSI) are at various stages of experimental structure determination. Classification of structures contributes significantly to the study of their evolutionary relatedness [2,3] and functional diversity. Structure Classification of Proteins (SCOP) [4] is the most established and popular scheme for classification. However, classification to SCOP has been largely manual, until a recent upgrade to its classification strategy when semi-automation was implemented; and full automation will eventually be needed [5,6]. Clearly, an automated and reliable classification technique would assist the expert human classifiers and speed up their job. It would be an added advantage if such a technique could predict plausible classifications and identify where rearrangements are needed in the existing classification hierarchy.

Efforts to automate structural classification have been ongoing for over a decade using supervised machine learning (ML) algorithms, such as neural networks [7-12] and support vector machines (SVMs) [13-17]. In general, in these studies, input to the ML algorithms is a one dimensional representation of the tertiary structure, in the form of various physicochemical and structural properties derived from the primary and secondary structure. Using a previously published data set [8], Shen and Chou [18] assigned proteins to one of the 27 most populated folds selected from SCOP. They proposed a weighted voting scheme, which combined SVM based classifiers trained independently on features such as pseudo amino acid composition [19], secondary structure state, hydrophobicity, polarity, polarizability and normalised van der Waals volume. The classification accuracy of 62.1% was higher than other studies [8,9]. Zhao *et al*. have applied ensemble learning to protein structure classification [20]. Such approaches have improved the classification accuracy to 75% [21]. A decision tree based ensemble classifier was proposed by Çamoğlu *et al*. [22], assigning proteins to existing families, super-families and folds with accuracies of 83%, 45% and 31%, respectively. All these efforts attempt binary classification limited to the most populated SCOP levels. Binary classification is possibly not sufficient for the less populated structural levels or for a hierarchical classification scheme. Multiple lineages may be found for multi-domain proteins. This complicates the classification further. Recently, random forest, a decision tree based ensemble learner, was used in predicting glycosylation sites [23], protein-protein interaction sites [24-26], DNA-binding residues [27], disordered regions [28] and enzyme discrimination [29]. These investigations suggest that the random forest is a robust and scalable classifier. In comparison to SVMs and neural networks, relatively few applications of random forest have been reported in structural bioinformatics.

Previously [30] we examined 15 supervised machine learning algorithms, including some, such as decision trees and rule learners, which had not been used before for structure classification. We introduced a one dimensional representation of protein domains, comprising structure and sequence based descriptors characterising the constituent secondary structure elements (SSEs). These features were distance, relative orientation, solvent accessibility, length, type of the SSEs and the level of sequence identity. For all of the structurally equivalent pairs of SSEs these features were calculated using DSSP [44] assignments. Structurally equivalent pairs of SSEs are defined as follows. Consider two domains,*A *and B, each comprising three SSEs: *A*_1_*A*_2_*A*_3 _and *B*_1_*B*_2_*B*_3_, where A1 is the SSE located at the N-terminus of A and so on. The structurally equivalent pairs are {*A*_1_*A*_2_, *B*_1_*B*_2_}, {*A*_1_*A*_3_, *B*_1_*B*_3_} and {*A*_2_*A*_3_, *B*_2_*B*_3_}. The domains being compared should have the same number of SSEs. Using such a representation, we exploited the similarity among the structurally equivalent pairs of SSEs of the two domains and assessed the predictive performance of the 15 algorithms. The random forest [31] outperformed contemporary supervised ML algorithms [30], such as neural networks and SVMs, for hierarchical SCOP structure classification. In contrast to SVMs, the performance of random forest remained unaffected by the imbalance in the dataset and the classification was an order of magnitude faster. On a dataset of domains containing three SSEs, a cross-validated accuracy of 97.0% and F-measures of 0.97, 0.85, 0.93 and 0.98 were observed for classification to the *Class*, *Fold*, *Super*-*Family *and *Family *level, respectively.

Random forest is an ensemble of decision trees, where every tree learns the classification on a random subset of the domain pairs. For every tree, at each splitting or decision node, the most discriminating descriptor is chosen from a randomly selected subset of *m *descriptors, where *m *is much smaller than the total number of descriptors. Decision nodes are added to each of the trees without pruning, until classification for every instance is obtained. This divide and conquer strategy [32,33] combined with the randomised attribute selection strategy automatically weights the domain pairs based on the probability that they share a particular structural level. The prediction probability and its use for the classification are further explained in the Methods section. Finally, for every domain pair, a majority voting algorithm collects the classification decision from each of the trees to make the final prediction.

Here, we treat structure classification as a typical data mining problem and investigate a real-world scenario where random forest can be used to automate SCOP structure classification. As described in an earlier study [30], we consider four structural levels: *Class (CL)*, *Fold (FO)*, *Super-family (SF) and Family (FA)*. These definitions are given elsewhere [4,6,34]. We use the same representation for a pair of domains in terms of sequence and structure based descriptors as described above and used in a previous study [30]. One of the domains serves as the *template *(with known SCOP classification) and the other as the target (for which classification is sought). A high similarity between the pairs of SSEs of the target and template, in terms of descriptors, implies they share a deep level in the classification hierarchy. A pair is termed a *CL*-, *FO*-, *SF*- or *FA*-pair, if the domains belong to the same *Class, Fold, Super-family or Family*, respectively. Pairs not sharing any of these four structure levels are termed *NA*-pairs.

Extending our previous work [30], in this paper we study different aspects of structural classification. For this we have designed four strategies utilising datasets from two consecutive versions of the SCOP database. The ability to differentiate types of pairs and the correct prediction of the shared structural level is reported in terms of precision, recall and Matthew's correlation coefficient (MCC). We address issues such as introduction of a new structural level in SCOP and the merger of singleton levels. Four of the 11 classes defined in SCOP are "Not a True Class" and serve as place-holders for structures awaiting a verified classification. For most of the *NA*-pairs, one of the domains in the pair belongs to a place-holder class of SCOP. Therefore, we focus on classification of domains from place-holder classes to true structural levels. We comprehensively assess the random forest for classifying small domains consisting of three SSEs, and we show that larger domains consisting of four, five and six SSEs are also classified successfully. In addition, we make blind predictions for the structures deposited in the PDB after the release of SCOP version 1.73, which obviously are not yet assigned to the SCOP hierarchy. The quality of the predicted structural levels is demonstrated by the structural similarity among the target and the template domains.

## Result

The performance of random forest has been evaluated using four different strategies to carry out blind predictions (Table 1). Strategies 1, 2 and 4 train random forest on a dataset derived from SCOP version 1.69 and test the model on the non-overlapping dataset derived from SCOP version 1.73. In strategy 3, both the training and test datasets are derived from SCOP version 1.69. However, the prediction has been attempted for those pairs which do not share any of the SCOP structural levels. The prediction accuracies for strategies 1 and 2 are reported in Table 2. As expected, the overall accuracy is higher in strategy 2 than in strategy 1. Both the precision and recall for the *CL*-pairs are higher; for the other types of pairs the difference is not significant. The confusion matrices for the two strategies are given in Table 3. In strategies 3 and 4, about 98% of the *NA*-pairs were predicted to share a class. The remaining 2% of the *NA*-pairs were predicted to share a fold or a family. The *NA*-pairs predicted to share a super-family were negligible in proportion. The pairs predicted to share a particular structural level were divided into four categories based on their prediction probabilities (*p*) (Table 4). In both of the strategies, no pair was predicted to be a *SF*-pair with p > 0.5. Therefore, the following section will discuss a few of the *NA *pairs classified as a *FO *or *FA*-pairs. In such pairs, the domain that belongs to a "True class" is considered as a template and the one belonging to "Not a true class" as a *target*. This exemplifies the use of the random forest to classify domains from the place-holder classes of SCOP. The pairs misclassified with p ≥ 0.9 are confident predictions, which may indicate a change is required in the classification. A shared level predicted lower in the hierarchy than the actual level in the SCOP may suggest a possible merger of the levels. For example, if domains *d*_1 _and *d*_2 _belonging to different folds *FO*_1 _and *FO*_2_, are predicted to be a *FO*-pair then the folds *FO*_1 _and *FO*_2 _may be considered for a possible merger. Similarly, a pair actually sharing a deeper level, but predicted to share the level above in the hierarchy, may indicate that there should be a new element at the deeper level. We attempted to verify the predicted structural levels of the *NA*-pairs by reference to the next official SCOP release (pre-SCOP) as well as to the recently released current version 1.75. However, no change in the status of the *NA*-pairs was found.

**Table 1:** Training and test sets used in the four evaluation strategies

Strategy	Training set	Test set	Training (Test) set size
1	DS1.69	DS1.73_*Uniq*_	6929 (6606)
2	DS1.69_*No - NA*_	DS1.73_*Unique - No - NA*_	4071 (4114)
3	DS1.69_*No - NA*_	DS1.69_*NA*_	4071 (2858)
4	DS1.69_*No - NA*_	DS1.73_*Unique - NA*_	4071 (4653)

DS1.69, set of domain pairs from SCOP version 1.69, DS1.73_*Uniq*_, set of domain pairs exclusive of SCOP version 1.73. DS1.69_*No - NA *_and DS1.73_*Unique - No - NA*_, are the respective DS1.69 and DS1.73_*Uniq *_sets but without *NA*-pairs. DS1.69_*NA *_and DS1.73_*Unique - NA*_, are sets of only *NA*-pairs from DS1.69 and DS1.73_*Uniq*_, respectively.

**Table 2:** SCOP inter-version blind prediction using random forest

**Shared SCOP Level**	**Evaluation Strategies (Accuracy)**					
	
	**Strategy 1 (85%)**			**Strategy 2 (93%)**		
	
	**Pre**	**Rec**	**MCC**	**Pre**	**Rec**	**MCC**
	
Class	0.89	0.84	0.73	0.94	0.99	0.81
Fold	0.86	0.45	0.61	0.93	0.41	0.61
Super-family	0.80	0.55	0.69	0.75	0.55	0.63
Family	0.82	0.87	0.83	0.84	0.85	0.83
None	0.81	0.91	0.76	n/a	n/a	n/a

The prediction accuracies and the class-wise performance of the random forest in strategies 1 and 2. Percent accuracy is the percentage of correctly classified domain pairs from all of the shared SCOP levels. Pre = Precision, Rec = Recall and MCC = Matthew's correlation coefficient.

**Table 3:** Confusion matrices for strategies 1 and 2

Strategy 1						
	**Predicted**					
	
		**CL**	**FO**	**SF**	**FA**	**NA**

**Actual**	CL	2813	10	0	12	511
	
	FO	92	125	1	30	30
	
	SF	21	2	56	23	0
	
	FA	30	5	13	336	4
	
	NA	216	3	0	9	2264

**Strategy 2**						

	**Predicted**					

		**CL**	**FO**	**SF**	**FA**	**NA**
	
**Actual**	CL	3327	4	1	14	-
	
	FO	134	115	6	23	-
	
	SF	21	1	56	24	-
	
	FA	44	3	12	329	-
	
	-	-	-	-	-	-

The confusion matrices used for classification of domain pairs according to their shared structural level following strategies 1 and 2. CL = shared *Class*, FO = shared *Fold*, SF = shared *Super*-*family*, FA = shared *Family *and NA = no shared structural level.

**Table 4:** Classification of NA-pairs in strategies 3 and 4 according to the probability estimates

Predicted Shared Level	Total	p *<*0.5	p = 0.5	0.5 *<*p *<*0.9	p ≥ 0.9
	
	Strategy 3				
Class	2806	14	130	1235	1427
Fold	29	0	6	9	14
Super-family	2	2	0	0	0
Family	21	0	8	12	1

	**Strategy 4**				

Class	4558	37	162	1917	2442
Fold	55	0	5	37	13
Super-family	3	3	0	0	0
Family	37	0	9	20	8

Following strategies 3 and 4, *NA*-pairs can be classified as sharing one of the top four SCOP structural levels. A large fraction of *NA*-pairs are classified to share the same Class in both the strategies. However, further distribution of such pairs according to the probability reflects the classification confidence. The high probability reflects more confidence in the predicted classification.

### Classification of larger domains

In order to classify a *target *domain following the proposed approach, a *template *domain with an equal number of SSEs should exist in SCOP. The approach has been validated in detail using domains consisting of three SSEs. Its application to classifying domains consisting of four, five and six SSEs shows its extensibility (Additional File 1, Table 1). The ten-fold stratified cross-validation accuracy obtained on these domains is given in Table 5 and the corresponding confusion matrices are listed in Table 2 in Additional file 1. The high classification accuracy and the class-wise values of MCC indicate the applicability of the approach to larger domains. The MCC and precision and recall values decrease as the number of SSEs in the domains increases. While the proportion of different types of pairs was almost the same in these larger domain datasets, *FO*- and *SF*-pairs were clearly minority classes (on average 2.5% of the total pairs). Nevertheless, random forest seemed to be efficient at classifying such an imbalanced set.

**Table 5:** Classification performance of the random forest on domains consisting of four, five and six SSEs in ten-fold cross-validation.

Shared SCOP Level	4SSEs			5SSEs			6SSEs		
	
	Accuracy = 98%			Accuracy = 98%			Accuracy = 97%		
	
	Pre	Rec	MCC	Pre	Rec	MCC	Pre	Rec	MCC
Class	0.99	0.99	0.92	0.98	1.00	0.89	0.97	1.00	0.85
Fold	0.96	0.83	0.89	1.00	0.69	0.82	0.95	0.51	0.70
Super-family	0.88	0.69	0.78	0.98	0.65	0.79	0.95	0.57	0.74
Family	0.98	0.92	0.95	0.98	0.92	0.94	0.98	0.84	0.90

Classification performance of the random forest on domains consisting of four, five and six SSEs in ten-fold cross-validation. Pre = Precision, Rec = Recall and MCC = Matthew's correlation coefficient.

### Structurally unclassified proteins from the PDB

For every target-template test pair, the random forest assigns a probability that it is a particular type of pair. The pair type associated with the highest probability is predicted for the pair. For three out of 21 domains, representing nine proteins containing three SSEs, family level predictions were made with a probability of 0.5 or above. A few of the predicted classifications for the nine target proteins are given in Table 6 (see also Additional files 2 and 3). The predicted classifications are the SCOP titles for the respective *template*'*s *structural level, determined by the predicted pair type. For example, for a predicted *FO*-pair, the *target *will be assigned to the same fold as that of the *template*. In general, a *target *is predicted to have the same structure level as all of the templates in that classification lineage(s) if they are in the test set (see the discussion of the triangle equality). However, sometimes the *target *pair with some other template(s) from the same lineage is predicted to a deeper, more specific level in the hierarchy. In such cases, the deeper level is selected to classify the *target*. In an alternative scenario, although the same structure level is predicted for the *target *and the *template *from a lineage, for some target-template pairs the prediction probabilities for the shared level are less than 0.8. If this is the case for multiple target-template pairs and the prediction probability for the level deeper in the hierarchy is greater than 0.2, the deeper level is used to suggest the classification of the *target*. Applying these methods, multiple folds (2JZ6, 2K2 D and 2K5J) and independent lineages (2ZM6 and 3BPJ) are identified as the potential classification for the *target*. Prediction of multiple folds is mainly due to the multi-domain nature of some of the *target *proteins.

**Table 6:** Classification for the selected unclassified target domains

Target PDB Id PDB Title		Predicted Classification (sunid)	Template
2JZ6	50S ribosomal protein L28	Scop_cf DNA/RNA-binding 3-helical bundle (46688)	1SAN, 1HOM
		scop_cf Spectrin repeat-like (46965)	1CUN, 1U4Q

2K2D	C-terminal domain of human pirh2	scop_cf Cupredoxin-like (49502)	1V54, 1OCR, 2DYS, 1OCC
		scop_cf Rubredoxin-like (57769)	2EIM, 2DYS, 1OCZ
		
		scop_cf Glucocorticoid receptor-like (DNA-binding domain) alpha+beta metal(zinc)-bound fold (57715)	1B8T

2K5J	Protein yiiF Uncharacterised protein	scop_cf DNA/RNA binding 3 helical bundle (46688)	1FJL, 1MBJ, 2DS5
		scop_sf "Winged helix" DNA binding domain (46785)	2GZW
		scop_cf Albumin binding domain like (46996)	1GJS(T), 1J78, 1MA9

2RPJ	Fn 14 Cystein Rich Domain (CRD)	scop_sf t-snare proteins (47661)	1S94, 1EZ3, 1BR0
		scop_cf Spectrin repeat-like (46965)	1E2A, 2E2A
2ZM6	30 S ribosomal subunit	Different scop_fa covering 22 lineages of various Ribosomal protein S(2-20) families*	1HNW, 2UU9, 1IBL 2F4V, 1XNQ, 2HGP, 2HGI

3BPJ	Human translation initiation factor 3	scop_cf Long alpha hairpin fold (46556)**	2OTJ, 1YHQ, 1VQK
		scop_cf Tetracyclin repressor-like (48497)	1ZK8

3H3M	Flagellar protein FliT	scop_fa Voltage-gated potassium channels (81323)	2HVK, 1JVM, 1R3J 1K4D
		
		scop_cf Spectrin repeat-like (46965)	1G73, 1FEW
		
		scop_fa MIT domain (116847)	1YXR

3ERM	Conserved protein with unknown function	scop_fa Myb/SANT domain (46739)	1IDY, 1MSE, 1MBJ
		scop_cf alpha-alpha superhelix (48370)	1HF8, 1HG5, 1HFA
		
		scop_fo Spectrin repeat-like (46965)	1E2A, 2E2A

3GI7	Secreted protein of unknown function	scop_cf DNA/RNA-binding 3-helical bundle (46688)	1P7I, 2HDD, 1DU0, 1FTT

Predicted classification for the selected unclassified target domains based on the classified domains (Template). Scop_cf= SCOP Fold, scop_sf = SCOP super-family and scop_fa = SCOP family.* Additional file 2 (2ZM6),** Additional file 3 (3BPJ).

**Figure 1:**
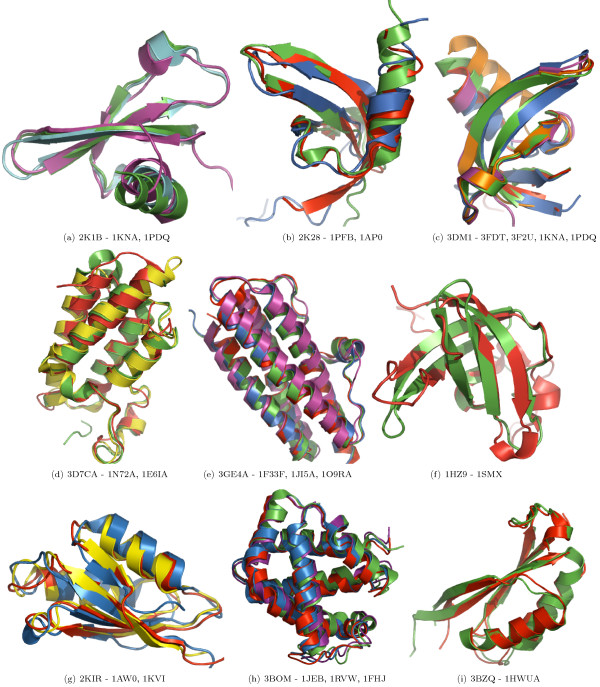
**Structural overlap of domains predicted to share the same SCOP Family**. Structural overlap of some of the selected target-template pairs may confirm the correctness of predicted shared *Family *level. Rows one, two and three show the overlap of domains consisting of four, five and six SSEs, respectively. The PDB identifier for the target is on the left-hand side of the sub-figure caption and for the template domain(s) it is on the right-hand side.

**Figure 2:**
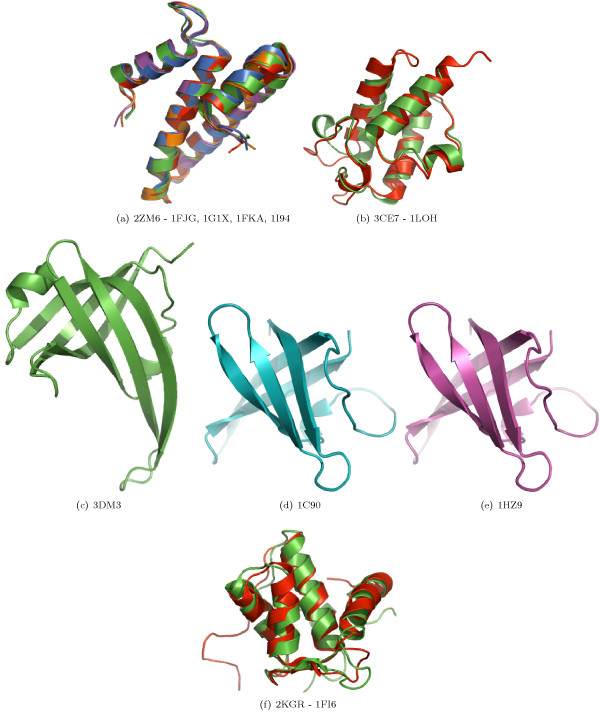
**Structural overlap of domains predicted to share the same SCOP Super-family**. Structural similarity of some of the selected target-template pairs. The target domains on the left were predicted to share the same super-family as the respective template domains on the right-hand side.

**Figure 3:**
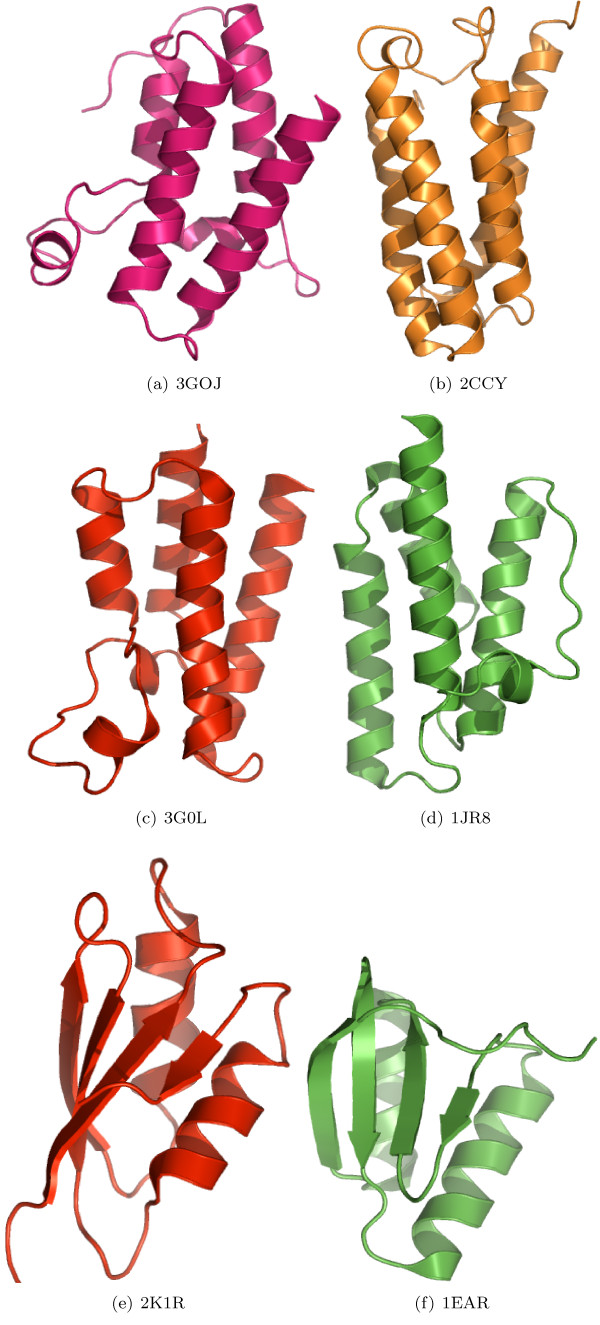
**Structural overlap of domains predicted to share the same SCOP Fold**. Structural similarity of some of the selected target-template pairs. The target domains on the left were predicted to share the same fold as the respective template domains on the right-hand side.

In the case of proteins with larger domains, predicted classifications can be verified on the basis of structural overlap and similarity. For nine of the 32 proteins containing domains consisting of four SSEs, the random forest predicted a structural family with a probability of 0.5 or above. For 11 out of 51 proteins containing domains consisting of five SSEs, and for six proteins out of 62 proteins with six SSEs a structural family was predicted with a probability of 0.5 or above. We believe that these predictions are correct, as suggested by the near perfect overlap of the target and template structures (Figure 1). The structural similarity of those target-template pairs which were predicted to be *FO*- or *SF*-pairs is shown in Figure 2, 3, respectively.

## Discussion

We compared the predicted classification to that defined in SCOP. Structural diversity within a *FO*, *SF *and *FA *level is a major source of difference between classification based on various structure similarity scores and expert knowledge [35-39]. Another contributing factor is the "triangle equality" that cannot be captured by a similarity score alone and requires expert knowledge [3]. A triangle relationship (equality) is the transitivity that is expected from an unbiased automated classification method e.g., if domains *d*_*i *_and *d*_*j *_are classified as a *FO*-pair and if domains *d*_*j *_and *d*_*k *_make a *FO*-pair, *d*_*i *_and *d*_*k *_should be predicted as a *FO*-pair. Irrespective of the agreement between the predicted and actual classification, the random forest was able to detect triangle relationships.

**Figure 4:**
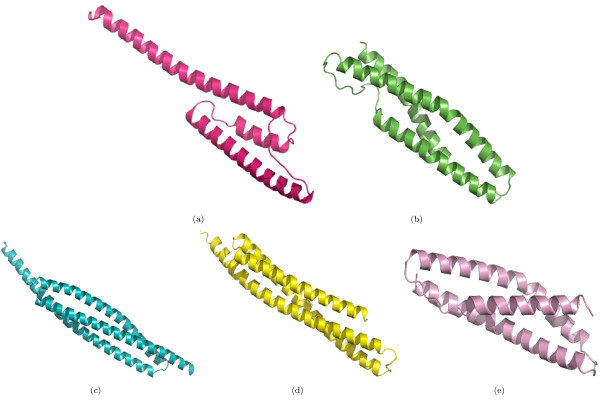
**Structural similarity among *NA*-pairs predicted to share the same SCOP Fold**. The domain d1lj2a (a) is predicted to share the same fold as d1hcia3 (b)(*Spectrin repeat*-*like*, sunid = 46965). The other domains d1fewa (c), d1g73a (d) and d1s35a (e) are also from the same fold and with them the overlapping domains in Figure 5c (d1ibmt (cyan), d1pnxt (magenta) and d1pnst (yellow)) are also predicted to share the *FO *level.

**Figure 5:**
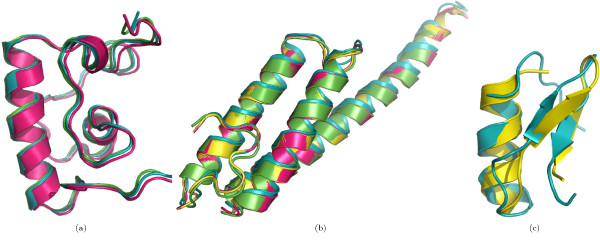
**Structural overlap of *NA*-pairs predicted to be FA-pairs**. (a) The domain d1ibkr (magenta) is currently classified such that it does not share any structural level with domains d1j5er (cyan) and d1n32r (green). However, we predict them to be *FA*-pairs. Similarly in (b) domains d1ibmt (cyan), d1pnxt (magenta) and d1pnst (yellow) do not currently share any structure level with the domain d2uuct1 (green, a SCOP 1.73 only domain). However, following strategy 4, the pairs of d1ibmt, d1pnxt and d1pnst with d2uuct1 are predicted to be *FA*-pairs sharing the *Ribosomal protein S20 *(sunid = 46993) family. (c) The domain d1d5qa (yellow) is a member of singleton family *Mini*-*protein reproducing the core of the CD4 surface *(sunid = 58922) under the not a true SCOP class *Designed proteins*. It and d2ptaa (magenta) are predicted to be a *FA*-pair belonging to the *Short*-*chain scorpion toxins*, (sunid = 57116) family.

**Figure 6:**
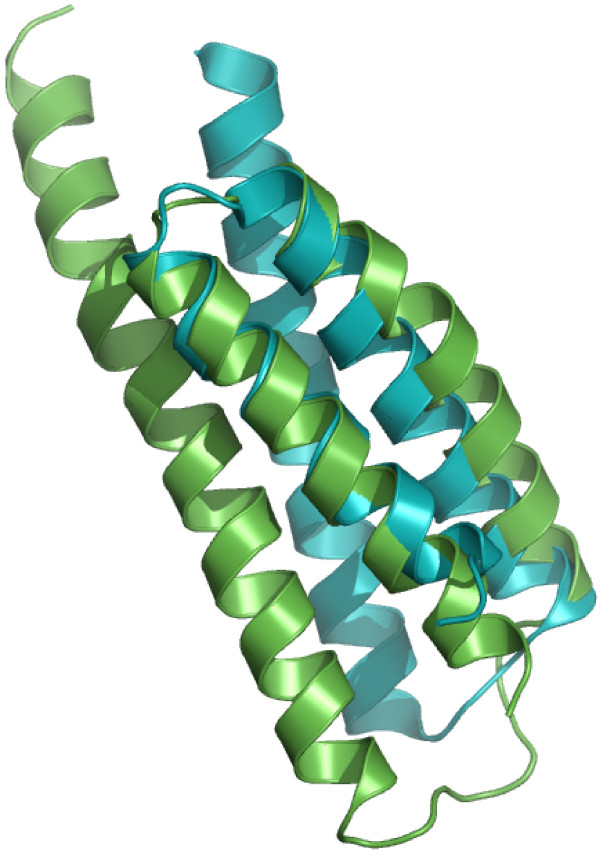
Structural similarity of the two domains d3h3ma (green) and d1yxr (cyan), predicted to share the same family MIT domain (suind = 116847)

The overall accuracies reported in strategies 1 and 2 are encouraging (Table 2). Due to the larger number of *CL*-, *FA*- and *NA*-pairs compared to the number of *FO*- and *SF*- pairs, we have reported the class-wise precision, recall and MCC for such pairs. The high precision indicates that the random forest can selectively identify a given type of pair, although this is at the expense of not identifying all of the pairs of that type (i.e., low recall). MCC reflects the balance in correct and incorrect classification when the classes are of unequal size and therefore is an indicator of a predictor's quality [40]. Based on these measures, the performance of random forest is better in predicting *CL *and *FA*-pairs than predicting *FO *and *SF*-pairs. Following strategy 3, some interesting predictions have been made. For example, the domain d1hcia does not share any structural level with d1lj2a and d1lj2b in SCOP version 1.73, but these pairs were predicted to be *FO*-pairs (Figure 4). Similarly, in SCOP version 1.69, the domain d1ibkr does not share any structural level with domains d1n32r and d1j5er, despite high structural similarity to one of the building blocks of the ribosomal complex (Figure 5a) from the same species (*Thermus thermophilus*). The former is a low resolution ribosomal complex and all of its 20 chains have been classified in SCOP's *Low resolution protein structures class*, "Not a true class". The random forest, however, predicted (*p *= 0.9) these domains to be members of the *Ribosomal protein S18 *(sunid = 46912) family. Although in the latest SCOP version 1.75 the classification for d1ibkr is same, our method predicts that in a future version it will move to a true structural class and perhaps to the same lineage to which d1j5er and d1n32r belong (a.7.6.1). Other examples are shown in Figure 5b. Similar observations are made from predictions following strategy 4. For example, the random forest classifies three domains d1ibmt (cyan), d1pnxt (magenta) and d1pnst (yellow) as belonging to a common family with the domain d2uuct1 (Ribosomal protein S20, sunid = 46993) (Figure 5c). The high structural similarity among the domains in all of these cases confirms the predictions. To exemplify the application of the approach to unclassified structures, we have classified proteins not yet assigned to the SCOP hierarchy (Table 6). For proteins with domains consisting of three, four, five and six SSEs, classification up to at least fold level was obtained. For a few cases, a family level classification could be suggested and was confirmed by the structural similarity (Figure 1). However, for the fold level prediction, instead of high structural similarity, topological similarity was observed (Figure 3 and Figure 6).

**Figure 7:**
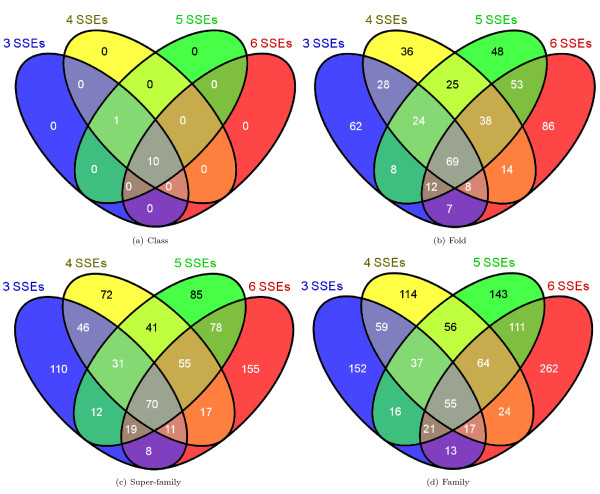
**Venn diagram showing number of different SCOP levels represented by the datasets used**. The overlap of *Class *(a), *Fold *(b), *Super*-*family *(c) or *Family *(d) levels represented by the datasets of domains consisting of three, four, five and six SSEs

Generally, domain pairs which can bind to similar ligands or constitute the same cellular organelle, such as ribosomal and transcription initiation complexes or those responsible for cytoskeletal structure, were predicted as *FO*-pairs. For example, 2K2 D possesses a zinc-binding fold and we found hits to proteins belonging to different lineages, namely cuperodoxin-like (b.6.1.2) and ruberodoxin-like (g.41.5.3) copper-and zinc-binding folds, respectively. These folds are characteristic of cytochrome c oxidases. 2JZ6 possesses two different folds which we assume (this protein is still unclassified) to be predicted correctly. Further, these two folds (sunid 46688 and 46965) have dedicated super-families to classify only those domains, which belong to the ribosomal complexes and perhaps provide a DNA/RNA binding site on ribosomes. The identification of folds in such cases could guide manual assignment of a super-family or family. The prediction of multiple folds having a related super-family, as in the case of 2JZ6 and 2ZM6, may reflect the capability of the random forest to identify classification networks. Similarly, 3BPJ, a human translation initiation factor is classified correctly to the singleton fold *Tetracyclin repressor*-*like*, *C*-*terminal domain *(sunid = 48497) and thereby to the family identified with the same name (sunid = 48499). In this case, the template domains used by the predictor were the *Transcriptional regulator protein *(1ZK8) from bacteria *Bacillus cereus *and also the other domains constituting the ribosomal complexes in different organisms (Table 6). Figure 6 shows the structure similarity between domains d3h3ma (from structurally unclassified protein 3H3 M, a agellar protein) and d1yxra (1YXR), thus supporting the predicted common family (*p *= 0.5, *MIT domain*, suind = 116847). In addition, d3h3ma is predicted to share the same fold (*Spectrin repeat*-*like*, sunid = 46965) as d1fewa and d1g73a (Figure 4c and Figure 4d, respectively), to which domains from various other cytoskeletal proteins in various families, including the *MIT domain family*, belong. We show that the proposed approach is extensible to larger domains. However, the number of SSEs in the target domain should be equal to the number of SSEs in template domains. We checked that this does not limit the approach in predicting only those structural levels which are either solely populated by domains consisting of a specific number of SSEs or are over-represented by such domains. Datasets of domains consisting of three, four, five and six SSEs, as used in this study, represent 1,144 of 3,464 families, 810 of 1,777 super-families, 518 of 1,086 folds and 11 of 11 classes as defined in SCOP version 1.73. The overlap among these datasets has been studied (Figure 7). Obviously, the assignment of SCOP levels is independent of the number of SSEs in the domains. However, the evolutionary constraints used to define the SCOP families and super-families and to some extent structural relatedness defining upper levels in the SCOP hierarchy can in some cases constrain classification of domains consisting of a defined number of SSEs to specific levels. The Venn diagram in Figure 7 shows that only about 5% of families, 8% of super-families and 13% of the folds are represented by all of the four datasets. Comparatively, a large proportion of SCOP levels was found to be populated by domains consisting of a fixed number of SSEs (Figure 7). Nevertheless, classification of domains with unequal number of SSEs will avoid using multiple classification models specific to a given number of constituent SSEs. It would also be advantageous for the classification of multi-domain proteins for which domain boundaries are yet to be defined.

## Conclusions

Determination of protein function based on structure has prompted the need for a repertoire of representative protein structures. Human expertise is key to the reliable classification of such a repertoire. Therefore, we do not propose that the current approach should replace expert knowledge, rather it may assist current manual and semi-automatic practices. Nevertheless, the accuracy of up to 94% is promising and highlights the potential of automated structure classification. Random forest can learn the triangle equality of two pairs with a common domain, thereby predicting the same classification for both the pairs. In the case of difficult targets (proteins with less than 25% sequence similarity), the predicted classification can minimise the number of probable levels for further consideration. Subsequent manual inspection of fewer levels would require less time.

We have analysed mis-classified structures instead of making a detailed comparison with other strategies for the automation of SCOP classification. This is mainly due to the difference in the dataset that we have used, which is not biased to proteins or domains from the most populated folds or families or from the true SCOP classes only. In contrast to the four-level hierarchical classification addressed by our work, other methods are restricted to classification to one or two structural levels. Our intention was also to identify hidden patterns of mis-prediction, which may be suggestive of new structural levels and/or rearrangement in the existing SCOP hierarchy. We have found such patterns and proposed classification of structures from place-holder classes to the true structural classes, although further work may be needed to prove that the mis-classification could guide the merger of a few singleton families, super-families and even folds. These observations are important in light of the foreseeable refinements in the SCOP hierarchy [34] and the rearrangements which occur with every major SCOP release [6]. Moreover, our method is capable of identifying different lineages for a protein with multiple domains. This is attractive, as interacting proteins often share domains and have similar domain architectures [41]. Probably, multi-domain proteins belonging to the same lineage can be studied further to identify interacting pairs. Lastly, we apply random forest to those proteins which are yet to be classified structurally and have extended it to domains consisting of up to six SSEs.

We, therefore, conclude that utilisation of a supervised ML algorithm such as random forest in the structure classification pipeline would accelerate the process. In future work, we will include a sub-structure search algorithm to compare domains consisting of unequal number of SSEs. Such an algorithm will be able to identify common cores, as well as the structural drift [42] among super-families and folds.

## Methods

### Datasets

Non-overlapping pairs of domains from two different versions of SCOP were used as the training and test sets. ASTRAL compendium [43] mappings for the two releases of the SCOP database, versions 1.69 and 1.73, were used to filter 1,394 and 1,988 domains, respectively, consisting of three SSEs (Additional files 4 and 5, respectively). The SSEs were assigned by DSSP [44]. A helix comprises a minimum of four residues and a strand a minimum of three. Only those pairs of these domains with a sequence identity below 35% were retained. This generated two sets (DS1.69 and DS1.73) of 6,929 and 12,115 domain pairs, respectively. These domain pairs represented six *Classes*, seven *Folds*, eight *Super*-*families *and 24 *Families *from the SCOP hierarchy. Every domain was represented by structural descriptors characterising the constituent SSEs. For every possible pair of the SSEs, the distance between their geometric centre of mass and the relative orientation in terms of the angle between the axes that pass through the terminal C_α _atoms were calculated using C_α _coordinates. For every SSE, the solvent accessibility, length and secondary structure state (binary, 0 if α-helix, 1 otherwise) were also used as structural descriptors. Finally, the representation of the two paired domains also includes the root mean square difference (RMSD) for the distance, orientation, solvent accessibility and length descriptors along with the sequence identity of the paired domains [30]. Thus, the number of features defining a pair of domains each with *N *SSEs is .

Random forest was also tested for classification of larger domains consisting of four, five and six SSEs from SCOP version 1.69. These domains are listed in Additional files 6, 7 and 8 respectively. Pairs of these domains with less than 35% sequence identity were used in a ten-fold cross-validation trial. Table 7 lists the composition of these datasets in terms of the number of different types of pairs

**Table 7:** Datasets of domains composed of four, five and six SSEs

Domain Pair Type	Number of SSEs		
	
	Four	Five	Six
*CL*-Pairs	5154	7494	8845
FO-Pairs	221	232	135
SF-Pairs	66	120	154
FA-Pairs	290	462	456

Number of different pairs in the datasets of domains composed of four, five and six SSEs.

### Sets of structurally unclassified proteins

Single and multi-domain proteins deposited after the release of SCOP version 1.73 were obtained. Since these proteins are not yet classified by SCOP, no domain boundaries are available and we considered every chain as a test domain. In the case of homomeric proteins, only the first chain was considered. Such domains or chains, consisting of three, four, five and six SSEs were filtered as individual sets. We name these sets as UnClassified Domains (UCD) datasets followed by the number of SSEs in domains constituting the set, that is, UCD3, UCD4, UCD5 and UCD6, respectively. UCD datasets were subjected to a BLAST search [45] (e-value threshold = 100) against a local database of SCOP domains consisting of the same number of SSEs as in the UCD dataset. The total number of BLAST hits obtained for the UCD3, UCD4, UCD5 and UCD6 datasets was 1,001, 1,060, 1,768 and 1,728, respectively. Pairs of database domains and UCD domains were obtained from these hits for subsequent use as the corresponding UCD test set. The selection of hits ignored sequence identity in order to maximise the number of pairs. The UCD3, UCD4, UCD5 and UCD6 test sets contained pairs for nine, 32, 51 and 62 unclassified proteins, respectively. Table 1 in Additional file 1 lists these proteins. The respective training sets were generated from SCOP version 1.69.

### Evaluation of random forest

The random forest algorithm, as implemented in Weka [46] developer version 3.5.7, without any model selection, was evaluated through four strategies (Table 1). The number of trees to construct the forest was left at the default, that is 10, and the number of features to define each of the nodes in a tree was also left at the default value of log_2_(*M *+ *1*) features. *M *is the number of features defining each of the pairs. Four evaluation strategies (Table 1) were adopted. In strategies 1, 2 and 4, domain pairs from DS1.69 and DS1.73 were used to generate the training and test sets, respectively. In strategy 3, the training and test sets both were derived from DS1.69. Strategy 1 takes into account all of the domain pairs from DS1.69 for training; only those pairs from DS1.73 which are not in DS1.69 are the test set (DS1.73_*Uniq*_). To accommodate new targets in the latest release of SCOP, new structural levels can be introduced with or without rearrangements in the classification hierarchy. This may also alter the classification of previously classified structures. Therefore, using an inter-version test set, it is possible to check if random forest can detect novel relationships that it was not explicitly trained on.

In SCOP, four out of 11 classes are regarded as "Not a true class" and are used as place-holders for domains until further information is available. However, in practice, predicting a structure to any of the "Not a true" class, fold, super-family or family level should be avoided. A predictor should be trained only on those structural levels to which domains have been classified in accordance with experimental, structural, sequence or knowledge-based evidence. Such levels are the "True" SCOP levels. Thus, removing *NA*-pairs (for most of which, one of the domains belongs to one of the place-holder classes) from training, as in strategy 2, would be more realistic. We expect some improvement in the classification accuracy, as most probably some of the *NA*-pairs share some structural features with other types of pairs i.e., *CL*-pairs or *FO*-pairs. In such cases, classification of an *NA*-pair to another type of pair will appear to be an incorrect prediction. To classify domains from place-holder classes, we evaluated the random forest, using DS1.69 with the *NA*-pairs removed as the training set and the *NA*-pairs removed from DS1.69 as the test set (strategy 3). With the same aim, in strategy 4, predictions were obtained for *NA*-pairs that only appear in DS1.73.

For strategies 1 and 2, the overall classification performance of the random forest is given in terms of percent accuracy, representing the fraction of correctly predicted domain pairs. This overall accuracy is a limited statistic, especially when classification is sought for four (strategies 2, 3 and 4) or five (strategy 1) types of pairs. The classification performance, therefore, has been measured independently for every type of pair. Thus, various measures of the multi-class classification performance are used, including precision (*Pre *= *TP*/(*TP *+ *FP*)), recall (*Rec *= *TP*/(*TP *+ *FN*)) and Matthew's correlation coefficient (MCC) (Equ. 1), where *TP *is the total number of true positives (instances correctly classified as belonging to a type of pairs), *TN *is the total number of true negatives, *FP *is the total number of false positives (instances incorrectly classified as belonging to a type of pairs) and *FN *is the total number of false negatives (instances incorrectly classified as not belonging to a type of pairs). MCC ranges from -1 to +1, and is a good measure of the quality of the predictor for a given a class. A perfect predictor will have an MCC of 1, whereas for a random predictor, the MCC will be zero.(1)

Measures used in strategies 1 and 2 are not applicable to strategies 3 and 4, which predict one of the top four structural levels for the classification of the *NA*-pairs. Thus, in strategies 3 and 4 the assessment was based on the prediction probabilities that random forest uses to classify each of the domain pairs. For a domain pair, the prediction probability is the average of the probabilities for classification to a particular structural level from all of the trees (in our case 10) in the forest. In every tree, the classification for every domain pair is given by the leaf node specific to a structural level. The leaf node assigns a probability, which is the ratio of instances belonging to a level to all of the instances that reach the leaf. In strategies 3 and 4, the *NA*-pairs which were predicted to one of the four levels with a prediction probability above 0.5 were considered as being predicted correctly. The correctness of the predicted level is checked by visual inspection. We have also checked the triangular equality of the predicted levels. Where possible, either a merger of the two SCOP levels or the addition of a new structural level to accommodate the *NA*-pairs is suggested based on a probability of 0.9 or above, which is indicative of a greater confidence in the prediction.

## Authors' contributions

JDH proposed the structural descriptors and PJ conceived their use for structural classification. PJ drafted the manuscript and JDH provided advice on presenting results and assisted in drafting the manuscript. Both the authors have read and approved the final version of the manuscript.

## Supplementary Material

Additional file 1 **Unclassified domains consisting of four, five and six SSEs**. This file lists the PDB identifiers for the unclassified proteins deposited in PDB after the release of SCOP 1.73 and the confusion matrices used to classify such proteins. Click here for file

Additional file 2 **Predicted classification for unclassified protein 2ZM6**. This file lists all of the classification hierarchies to which the protein 2HGP is classified in the SCOP version 1.73. These classification hierarchies can be manually checked to classify 2ZM6 that is predicted to share the same *Family *as 2HGP. Click here for file

Additional file 3 **Predicted classification for unclassified protein 3BPJ**. This file lists all of the classification hierarchies to which the protein 2OTJ is classified in the SCOP version 1.73. These classification hierarchies can be manually checked to classify 3BPJ that is predicted to share the same *Fold *as 2OTJ. Click here for file

Additional file 4 **Domains consisting of 3SSEs from SCOP version 1.69**. This file lists the identifiers for the 3SSEs containing domains from SCOP version 1.69. Click here for file

Additional file 5 **Domains consisting of 3SSEs from SCOP version 1.73**. This file lists the identifiers for the 3SSEs containing domains from SCOP version 1.73. Click here for file

Additional file 6 **Domains consisting of 4SSEs from SCOP version 1.69**. This file lists the identifiers for the 4SSEs containing domains from SCOP version 1.69. Click here for file

Additional file 7 **Domains consisting of 5SSEs from SCOP version 1.69**. This file lists the identifiers for the 5SSEs containing domains from SCOP version 1.69. Click here for file

Additional file 8 **Domains consisting of 6SSEs from SCOP version 1.69**. This file lists the identifiers for the 6SSEs containing domains from SCOP version 1.69. Click here for file
