# Supplementary material for: Automatic structure classification of small proteins using random forest
Source: BMC Bioinformatics. 2010 Jul 1;11:364. doi: 10.1186/1471-2105-11-364 (PMC2916923; doi:10.1186/1471-2105-11-364)
Supplement: Additional file 1 — Unclassified domains consisting of four, five and six SSEs. This file lists the PDB identifiers for the unclassified proteins deposited in PDB after the release of SCOP 1.73 and the confusion matrices used to classify such proteins. [file 1471-2105-11-364-S1.PDF]

Additional File 1

Structurally unclassified Proteins

3SSEs	4SSEs	5SSEs	Conti.. 5SSEs	6SSEs	Conti.. 6SSEs
2JZ6	2JZ8	2JY9	3D7C	2K0S	3D0F
2K2D	2K1B	2JYA	3DKA	2K1R	3D3R
2K5J	2K28	2JZ5	3DM3	2K2B	3D7I
2RPJ	2K53	2K1H	3E0Z	2K50	3DAI
2ZM6	2K5E	2K2P	3EUS	2K5C	3DFE
3BPJ	2K7I	2K3I	3F42	2K5Q	3DI5
3ERM	2KCI	2K52	3F6W	2K75	3DKM
3GI7	2KCM	2K57	3FDR	2K8S	3DM3
3H3M	2KGO	2K5H	3FIF	2KAT	3DT5
	2KHQ	2K5N	3FKM	2KC7	3DWY
	2KHR	2K5R	3FVB	2KCK	3E0E
	2ZET	2K87	3FXH	2KDN	3EGP
	2ZM6	2K9Q	3FYB	2KE0	3EN2
	3BQS	2KAF	3G0J	2KEN	3F42
	3BS3	2KCT	3G0L	2KEY	3FB2
	3C90	2KCV	3GE4	2KGR	3FCN
	3CE7	2KCX	3GG3	2ZM6	3FH3
	3D0W	2KD0	3GHD	2ZOP	3FHW
	3DM1	2KD1	3GPV	3BOM	3FIF
	3EGR	2KHV		3BOQ	3FM5
	3EPY	2ROR		3BPD	3FN2
	3ERM	2RPC		3BPU	3FOG
	3EVY	2ZET		3BT5	3FRW
	3F2U	2ZM6		3BV8	3FYB
	3F75	3C4S		3BZQ	3G1C
	3FDT	3C90		3C4R	3G1J
	3FIA	3CAM		3C5K	3GAN
	3FLV	3CAZ		3C9P	3GGE
	3G0J	3CEC		3CAZ	3GP4
	3G27	3CP0		3CE8	3GPV
	3GV6	3CQ1		3CJL	
	3H87	3D3R		3CTV	

Table 1: Structurally unclassified proteins deposited in PDB after the release of SCOP 1.73 for which the classification using random forest was attempted.

Confusion matrices from ten-fold cross-validation classification using larger domains

4SSEs

		Predicted			
		CL	FO	SF	FA
**Actual**	CL	5146	3	1	4
	FO	37	184	0	0
	SF	17	2	46	1
	FA	16	1	5	268

5SSEs

		Predicted			
		CL	FO	SF	FA
**Actual**	CL	7493	0	0	1
	FO	70	160	0	2
	SF	39	0	78	3
	FA	36	0	1	425

6SSEs

		Predicted			
		CL	FO	SF	FA
**Actual**	CL	8843	1	0	1
	FO	64	70	0	1
	SF	62	0	89	3
	FA	66	2	4	384

Table 2: Confusion matrices from ten-fold cross-validation classification of domain pairs with four, five and six SSEs, according to their shared structural level. CL = shared *Class*, FO = shared *Fold*, SF = shared *Super-family* and FA = shared *Family*
