# Supplementary material for: Automatic structure classification of small proteins using random forest
Source: BMC Bioinformatics. 2010 Jul 1;11:364. doi: 10.1186/1471-2105-11-364 (PMC2916923; doi:10.1186/1471-2105-11-364)
Supplement: Additional file 2 — Predicted classification for unclassified protein 2ZM6. This file lists all of the classification hierarchies to which the protein 2HGP is classified in the SCOP version 1.73. These classification hierarchies can be manually checked to classify 2ZM6 that is predicted to share the same Family as 2HGP. [file 1471-2105-11-364-S2.PDF]

Additional File 2

Search Results for “2hgp” [scop 1.73]

Ribosomal protein S18 from Thermus thermophilus [TaxId: 274] [a.4.8.1]
Ribosomal protein S13 from Thermus thermophilus [TaxId: 274] [a.156.1.1]
Ribosomal protein S20 from Thermus thermophilus [TaxId: 274] [a.7.6.1]
Ribosomal protein S15 from Thermus thermophilus [TaxId: 274] [a.16.1.2] Ribosomal protein S7 from Thermus thermophilus [TaxId: 274] [a.75.1.1]
Ribosomal protein S12 from Thermus thermophilus [TaxId: 274] [b.40.4.5] Ribosomal protein S17 from Thermus thermophilus [TaxId: 274] [b.40.4.5] Ribosomal protein S2 from Thermus thermophilus [TaxId: 274] [c.23.15.1]
Ribosomal protein S11 from Thermus thermophilus [TaxId: 274] [c.55.4.1]
Ribosomal protein S5, C-terminal domain from Thermus thermophilus [TaxId: 274] [d.14.1.1]
Ribosomal protein S9 from Thermus thermophilus [TaxId: 274] [d.14.1.1]
Ribosomal protein S16 from Thermus thermophilus [TaxId: 274] [d.27.1.1]
Ribosomal protein S19 from Thermus thermophilus [TaxId: 274] [d.28.1.1]
Ribosomal S5 protein, N-terminal domain from Thermus thermophilus [TaxId: 274] [d.50.1.2]
Ribosomal protein S3 N-terminal domain from Thermus thermophilus [TaxId: 274] [d.52.3.1]
Ribosomal protein S3 C-terminal domain from Thermus thermophilus [TaxId: 274] [d.53.1.1]
Ribosomal protein S6 from Thermus thermophilus [TaxId: 274] [d.58.14.1]
Ribosomal protein S10 from Thermus thermophilus [TaxId: 274] [d.58.15.1]
Ribosomal protein S4 from Thermus thermophilus [TaxId: 274] [d.66.1.2] Ribosomal protein S8 from Thermus thermophilus [TaxId: 274] [d.140.1.1]
Ribosomal protein S14 from Thermus thermophilus [TaxId: 274] [g.39.1.7] 30S ribosomal protein THX from Thermus thermophilus [TaxId: 274] [j.9.1.1]

Potential Targets suggesting a common fold

d1u4qa3, d1cuna2 d2crba1

Potential Targets suggesting a common super family

d2j02r1, d1n32r, d1iblr, d2hgpu1, d1ibkr

Potential Targets suggesting a common family

d1hnwr, d2hgru1, d2uubr1, d1hr0r, d1hnzr, d1xmor, d1n34r, d2uxcr1, d1j5er, d1pnxr, d1pnsr, d1hnxr , dd2f4vr1, d2uuar1, d2uu9r1, 2uucr1, d1xnqr, d1fjgr, d2hhhr1, d2hgiu1, d2e5lr1, d1xnrr , d1xmqr, d1yl4u1
