# Supplementary material for: Automatic structure classification of small proteins using random forest
Source: BMC Bioinformatics. 2010 Jul 1;11:364. doi: 10.1186/1471-2105-11-364 (PMC2916923; doi:10.1186/1471-2105-11-364)
Supplement: Additional file 3 — Predicted classification for unclassified protein 3BPJ. This file lists all of the classification hierarchies to which the protein 2OTJ is classified in the SCOP version 1.73. These classification hierarchies can be manually checked to classify 3BPJ that is predicted to share the same Fold as 2OTJ. [file 1471-2105-11-364-S3.PDF]

Additional File 3

Search Results for “2otj” [scop 1.73]

Ribosomal protein L29 (L29p) from Archaeon Haloarcula marismortui [TaxId: 2238] [a.2.2.1] Ribosomal protein L11, C-terminal domain from Archaeon Haloarcula marismortui [TaxId: 2238] [a.4.7.1]
Ribosomal protein L19 (L19e) from Archaeon Haloarcula marismortui [TaxId: 2238] [a.94.1.1]
Ribosomal proteins L24 (L24p) from Archaeon Haloarcula marismortui [TaxId: 2238] [b.34.5.1]
Ribosomal proteins L21e from Archaeon Haloarcula marismortui [TaxId: 2238] [b.34.5.1]
C-terminal domain of ribosomal protein L2 from Archaeon Haloarcula marismortui [TaxId: 2238] [b.34.5.3] Ribosomal protein L14 from Archaeon Haloarcula marismortui [TaxId: 2238] [b.39.1.1]
N-terminal domain of ribosomal protein L2 from Archaeon Haloarcula marismortui [TaxId: 2238] [b.40.4.5] Ribosomal protein L3 from Archaeon Haloarcula marismortui [TaxId: 2238] [b.43.3.2]
Ribosomal protein L32e from Archaeon Haloarcula marismortui [TaxId: 2238] [c.9.2.1]
Ribosomal protein L15 (L15p) from Archaeon Haloarcula marismortui [TaxId: 2238] [c.12.1.1]
Ribosomal protein L18e from Archaeon Haloarcula marismortui [TaxId: 2238] [c.12.1.1]
Ribosomal protein L13 from Archaeon Haloarcula marismortui [TaxId: 2238] [c.21.1.1]
Ribosomal protein L4 from Archaeon Haloarcula marismortui [TaxId: 2238] [c.22.1.1]
Ribosomal protein L18 (L18p) from Archaeon Haloarcula marismortui [TaxId: 2238] [c.55.4.1]
Ribosomal protein L23 from Archaeon Haloarcula marismortui [TaxId: 2238] [d.12.1.1]
Ribosomal protein L15e from Archaeon Haloarcula marismortui [TaxId: 2238] [d.12.1.2] Ribosomal protein L31e from Archaeon Haloarcula marismortui [TaxId: 2238] [d.29.1.1]
Ribosomal protein L10e from Archaeon Haloarcula marismortui [TaxId: 2238] [d.41.4.1]
Ribosomal protein L22 from Archaeon Haloarcula marismortui TaxId: 2238] [d.55.1.1]
Archaeal L30 (L30a) from Archaeon Haloarcula marismortui [TaxId: 2238] [d.59.1.1]
Ribosomal protein L5 from Archaeon Haloarcula marismortui [TaxId: 2238] [d.77.1.1]
Ribosomal protein L7ae from Archaeon Haloarcula marismortui [TaxId: 2238] [d.79.3.1]
Ribosomal protein L6 from Archaeon Haloarcula marismortui [TaxId: 2238] [d.141.1.1]
Ribosomal protein L24e from Archaeon Haloarcula marismortui [TaxId: 2238] [g.39.1.6] Ribosomal protein L37ae from Archaeon Haloarcula marismortui [TaxId: 2238] [g.41.8.1]
Ribosomal protein L37e from Archaeon Haloarcula marismortui [TaxId: 2238] [g.41.8.2] Ribosomal protein L44e from Archaeon Haloarcula marismortui [TaxId: 2238] [g.41.8.3]

Potential Targets suggesting a common super-family

d2otjv1, d1yhqv, 1d1vqkv1, d1vqnv1, d1vqlv1, d1vqmv1, d1yjwv1, d1kc8w, d1qvfu, d1q86w.
