# Supplementary material for: Automatic structure classification of small proteins using random forest
Source: BMC Bioinformatics. 2010 Jul 1;11:364. doi: 10.1186/1471-2105-11-364 (PMC2916923; doi:10.1186/1471-2105-11-364)
Supplement: Additional file 4 — Domains consisting of 3SSEs from SCOP version 1.69. This file lists the identifiers for the 3SSEs containing domains from SCOP version 1.69. [file 1471-2105-11-364-S4.PDF]

Additional File 4

Domains consisting of 3SSEs from SCOP version 1.69

Table 1: Domains consisting of 3SSEs from SCOP version 1.69

**Domain Identifiers**							
d1a0rg_	d1a62a1	d1a6qa1	d1a7wa_	d1acwa_	d1ahdp_	d1aila_	d1aipc1
d1akha_	d1aoib_	d1aoif_	d1ap5b1	d1ap6b1	d1aplc_	d1au7a1	d1avya_
d1b67a_	d1b67b_	d1b6wa_	d1b8ia_	d1b8ib_	d1b9xb_	d1b9yb_	d1ba5a_
d1bcch_	d1bdca_	d1be3h_	d1bfma_	d1bgyh_	d1bhaa_	d1bhba_	d1bika1
d1bpta_	d1br0a_	d1bs3a1	d1bsma1	d1bt8a1	d1bunb_	d1bw5a_	d1c0wc2
d1c49a_	d1c7ya1	d1ca0d_	d1chla_	d1chua1	d1ck7a6	d1ckta_	d1cmbb_
d1coja1	d1cqta1	d1cuna1	d1cuna2	d1cunb2	d1cunc2	d1cxzb_	d1cyca_
d1d6ba_	d1dd3a1	d1dd4b1	d1ddna2	d1deeh_	d1dema_	d1dena_	d1dfaa2
d1dkyb1	d1dpra2	d1dprb1	d1dtka_	d1dtxa_	d1du0a_	d1du0b_	d1du2a_
d1dwma_	d1e0ha_	d1e0na_	d1e15a1	d1e1qg_	d1e2aa_	d1e3ha1	d1e3oc1
d1e52a_	d1e6na1	d1e6pa1	d1e6ra1	d1e6za1	d1e7da1	d1eawb_	d1ecma_
d1edia_	d1edja_	d1edka_	d1edla_	d1ef4a_	d1efub3	d1egfa_	d1en7a1
d1enka_	d1eqzd_	d1eqzh_	d1erca_	d1erpa_	d1esxa_	d1ev0a_	d1ez3a_
d1ezvi_	d1f2ig1	d1f2ik1	d1f43a_	d1f4ia_	d1f5ta2	d1f66b_	d1f66f_
d1f8ab1	d1fafa_	d1faki_	d1fewa_	d1fexa_	d1fjgt_	d1fjla_	d1fjlb_
d1fjna_	d1fpoa2	d1fqva1	d1fs2a1	d1fsef_	d1ftta_	d1ftza_	d1fx0b1
d1g2c.1	d1g2ha_	d1g3sa2	d1g3wa2	d1g73a_	d1gaba_	d1gaxb4	d1gjsa_
d1gjza_	d1gn2a1	d1gn3a1	d1gn4a1	d1gn6a1	d1goia1	d1gotg_	d1gpfa1
d1gt0c1	d1gt0d_	d1guua_	d1gv2a1	d1gv2a2	d1gv5a_	d1gvda_	d1gvna_
d1h0ia1	d1h0ta_	d1h3ob_	d1h5oa_	d1h88c1	d1h88c2	d1h88c3	d1h89c2
d1h8ac1	d1h8ac2	d1h8hg_	d1h9ta1	d1hbwa_	d1hcia3	d1hcia4	d1hcib4
d1hddc_	d1hdpa_	d1hf0a1	d1hf8a1	d1hfaa1	d1hg2a1	d1hg5a1	d1hgza_
d1hmaa_	d1hmea_	d1hmfa_	d1hnwt_	d1hnxt_	d1hnzt_	d1homa_	d1hq3d_
d1hrya_	d1hrza_	d1hsma_	d1htaa_	d1htya1	d1hwwa1	d1hxka1	d1hypa_
d1i1ga1	d1i5hw_	d1i6za_	d1i94t_	d1ibkr_	d1ibkt_	d1iblr_	d1iblt_
d1ic8a1	d1icfi_	d1id3b_	d1id3f_	d1idsa1	d1idya_	d1idza_	d1ig7a_
d1ihra_	d1iiea_	d1iioa_	d1ijwc_	d1irza_	d1itya_	d1iufa1	d1iufa2
d1iv6a_	d1ivsa1	d1iw7f1	d1ixrb1	d1j1dc_	d1j1ec_	d1j3ca_	d1j3da_
d1j46a_	d1j47a_	d1j5et_	d1j5na_	d1j78a3	d1j7ea3	d1j95a_	d1jc6a_
d1jeqa1	d1jfia_	d1jgga_	d1jj2u_	d1jj6c_	d1jjra_	d1jkpc_	d1jkrc_
d1jm7a_	d1jmla_	d1joya_	d1jt6b1	d1jtxb1	d1jvma_	d1jvmb_	d1jvmd_
d1k36a_	d1k37a_	d1k4cc_	d1k4dc_	d1k61a_	d1k61c_	d1k6ya1	d1k73w_
d1k78i1	d1k8aw_	d1k99a_	d1k9mw_	d1kb9f_	d1kb9i_	d1kbta_	d1kc8w_
d1kdxa_	d1kkca1	d1kmhb1	d1knpa1	d1knra1	d1knta_	d1kqma1	d1kqsu_
d1ktha_	d1ku2a1	d1ku3a_	d1ku5a_	d1kuna_	d1kvd.1	d1kve.1	d1kw2a3
d1kx3f_	d1kx4b_	d1kx4f_	d1kx5b_	d1kx7a_	d1kxpd3	d1kyof_	d1kyoi_
d1l0lj_	d1l0nh_	d1l0nj_	d1l0oc_	d1l4ab_	d1l6ha_	d1l6ja1	d1l6kc_
d1l8ya_	d1l8za_	d1lbua1	d1lcca_	d1lcda_	d1ld5a_	d1ldkd2	d1ldke1
d1lfba_	d1lg4a_	d1lira_	d1lj2a_	d1lj2b_	d1llmc1	d1lmja2	d1lota3
d1lq7a_	d1lqca_	d1lr1a_	d1lrea_	d1lt9d_	d1ltjd_	d1luvb1	d1lvfa_
d1lwua_	d1lwue2	d1lwuh2	d1m18b_	d1m18f_	d1m19b_	d1m19f_	d1m1ab_
d1m1ja_	d1m1jc2	d1m1kw_	d1m1pa_	d1m1pb_	d1m1qa_	d1m1ra_	d1m2sa_

Continued on Next Page…

Table 1 – Continued

Domain Identifiers

d1m62a_	d1m7ka_	d1m8la_	d1m90w_	d1ma1a1	d1ma9a3	d1mbea_	d1mbfa_
d1mbha_	d1mbja_	d1mbka_	d1mdma1	d1mg1a2	d1mh3a1	d1mh4a1	d1mjea2
d1mjlb_	d1mjqd_	d1mm0a_	d1mn3a_	d1mntb_	d1msda1	d1msec1	d1msec2
d1msfc2	d1msla_	d1mt1.1	d1mt1.3	d1mt1.4	d1mt1.5	d1mtnd_	d1mxma3
d1n13.1	d1n13.3	d1n13.4	d1n13.5	d1n13.6	d1n1jb_	d1n32r_	d1n32s_
d1n33c1	d1n33s_	d1n33t_	d1n34t_	d1n36t_	d1n5ga_	d1n86e2	d1n8la_
d1n8rw_	d1n98.1	d1naga_	d1nbmg_	d1ngmb_	d1ngmj_	d1nh2b_	d1nh2c_
d1nhma_	d1nhna_	d1ni8a_	d1njiw_	d1nk2p_	d1nk3p_	d1nl0g_	d1no1a_
d1ns1a_	d1ntkj_	d1ntkk_	d1ntmh_	d1ntmj_	d1ntmk_	d1ntzh_	d1ntzk_
d1nu1k_	d1nu7d1	d1nu9c1	d1nvma1	d1nvob_	d1nvpc_	d1nysd_	d1o3xa_
d1o4xb_	d1o6ia1	d1o6ua1	d1o6wa1	d1o7zb_	d1oa55_	d1oa65_	d1oaia_
d1ocoh_	d1ocoj_	d1ocow_	d1ocpa_	d1ocrb2	d1ocrh_	d1ocri_	d1ocru_
d1octc1	d1oczh_	d1oczw_	d1ofcx1	d1ogba1	d1ogga1	d1ohhg_	d1ohzb_
d1oksa_	d1olma1	d1olza3	d1on1a1	d1oo3a_	d1op1a_	d1oqdl_	d1osla_
d1ov2a_	d1ov3a1	d1ov9a_	d1ov9b_	d1ovxa_	d1p2ii_	d1p2ji_	d1p2ki_
d1p2nb_	d1p2ob_	d1p2qb_	d1p34b_	d1p3ab_	d1p3bb_	d1p3bf_	d1p3fb_
d1p3gf_	d1p3ib_	d1p3kb_	d1p3lb_	d1p3lf_	d1p3mb_	d1p3mf_	d1p3ob_
d1p3pb_	d1p7ga1	d1p7ia_	d1p7ib_	d1p7ic_	d1p7id_	d1p7ja_	d1p84i_
d1p9qc1	d1p9za_	d1pgya_	d1pina1	d1pira_	d1pita_	d1pjva_	d1pl4b1
d1pnha_	d1pnst_	d1pnxt_	d1poga_	d1pon.1	d1pp9h_	d1pp9j_	d1pp9w_
d1ppjw_	d1prba_	d1prua_	d1prva_	d1ps2a_	d1ps3a1	d1pufa_	d1puoa2
d1pvoa1	d1q1va_	d1q2na_	d1q7yw_	d1q81w_	d1q82w_	d1q86w_	d1q8ha_
d1qgwb_	d1qkha_	d1qkya_	d1qlqa_	d1qnma1	d1qp8b2	d1qrva_	d1qrya_
d1qu1a_	d1qu1e_	d1qu1f_	d1qvfu_	d1qvgu_	d1qvhh_	d1qvub1	d1qwna1
d1qx1a1	d1qzea1	d1qzpa_	d1r0bi2	d1r1ga_	d1r3ic_	d1r3jc_	d1r3kc_
d1r49a1	d1r4ag_	d1r4ga_	d1r73a_	d1re4d_	d1resa_	d1reta_	d1rf8b_
d1rj9b1	d1rk4a2	d1rkwb1	d1rp3a1	d1rp3a3	d1rp3c3	d1rp3e3	d1rp3g1
d1rqva1	d1rrza_	d1rsoa_	d1rsob_	d1rsod_	d1s0yb_	d1s1cx_	d1s1ho_
d1s32f_	d1s35a1	d1s5hc_	d1s72v_	d1s94a_	d1s94b_	d1sa0e_	d1sana_
d1sc5a1	d1scya_	d1sddb1	d1sfkb_	d1sfke_	d1sfva_	d1shpa_	d1shyb2
d1smyf1	d1smyf2	d1smyp2	d1sn9c_	d1snab_	d1snea_	d1sp4.1	d1sqbh_
d1sr9a1	d1ss1a_	d1sssa1	d1t6oa_	d1t95a1	d1tafa_	d1tapa_	d1tawb_
d1tbgg_	d1tbgh_	d1tc3c_	d1tdha3	d1tf0b_	d1tf3a3	d1tf6a3	d1tfxc_
d1tocr2	d1tocs2	d1toct2	d1tocu2	d1tpai_	d1trla_	d1ttea1	d1txaa_
d1tzyd_	d1u5td2	d1u78a1	d1u78a2	d1ud0a_	d1ug2a_	d1uhsa_	d1ujwb_
d1umqa_	d1unda_	d1uptb_	d1uptd_	d1upth_	d1ur8a1	d1ur9a1	d1ut3a_
d1uuba_	d1uura1	d1uusa1	d1uxca_	d1uzca_	d1v54h_	d1v54i_	d1v54v_
d1v55i_	d1v55v_	d1v92a_	d1vf6a_	d1vf6c_	d1vf6d_	d1vh6b_	d1vnda_
d1w0aa_	d1w0ba_	d1w0kg_	d1w2bu_	d1wdfa_	d1wdfb_	d1wdga_	d1wjfa_
d1xbla_	d1yrna_	d1ytfc_	d1ytfd2	d1zaac1	d1zeia_	d1zwaa_	d2a3da_
d2bcch_	d2bccj_	d2cpba_	d2crda_	d2drpa2	d2dtra2	d2e2aa_	d2eboa_
d2erla_	d2gata_	d2gf1a_	d2hdda_	d2hexa_	d2hiod_	d2hoaa_	d2hp8a_
d2knta_	d2ktxa_	d2lefa_	d2lfba_	d2occb2	d2occh_	d2occi_	d2occu_
d2ptaa_	d2ptci_	d2spza_	d2tgpi_	d2tpii_	d2trcg_	d3bcch_	d3bccj_
d3btei_	d3btfi_	d3btgi_	d3bthi_	d3btki_	d3btmi_	d3btqi_	d3btti_
d3egfa_	d3gata_	d3hdda_	d3lria_	d3mon.5	d3sdpa1	d3tgii_	d3tgji_
d3tpii_	d4ptia_	d4tpii_	d5ptia_	d5znfa_	d6inse_	d6paxa2	d6ptia_

Continued on Next Page…

Table 1 – Continued

Domain Identifiers

d7znfa_	d8ptia_	d9anta_	d9ptia_	d1a0aa_	d1a0ha1	d1a0rg_	d1a1fa1
d1a1ia2	d1a1ka1	d1a1la2	d1a5ja1	d1a62a1	d1a63a1	d1a6qa1	d1a7ia2
d1a8va1	d1aaba_	d1aala_	d1aapa_	d1aaya1	d1advb2	d1adza_	d1af8a_
d1aila_	d1aipc1	d1aj3a_	d1akha_	d1an4b_	d1anva2	d1aoib_	d1aoif_
d1ap6b1	d1aplc_	d1ar4a1	d1ar5a1	d1ar7.1	d1arfa_	d1atya_	d1au7a1
d1avya_	d1avyb_	d1ayga_	d1azka_	d1b06a1	d1b0ca_	d1b13a_	d1b2ia_
d1b4oa_	d1b50a_	d1b67a_	d1b67b_	d1b6wa_	d1b71a2	d1b72a_	d1b8ia_
d1b8ta4	d1b9xb_	d1b9yb_	d1ba5a_	d1bboa2	d1bbya_	d1bcch_	d1bccj_
d1bdda_	d1bdsa_	d1be3h_	d1bf0a_	d1bfma_	d1bg5a2	d1bgka_	d1bgyh_
d1bhba_	d1bhca_	d1bhgb1	d1bhia_	d1bika1	d1bl8a_	d1boea_	d1bpia_
d1bq0a_	d1bq8a_	d1br0a_	d1brbi_	d1bs3a1	d1bsma1	d1bt8a1	d1bthp_
d1bunb_	d1busa_	d1bw5a_	d1bz5a_	d1bzxi_	d1c04c_	d1c0wc2	d1c17a_
d1c56a_	d1c6sa_	d1c7ya1	d1ca0d_	d1cbwd_	d1chla_	d1chua1	d1ck7a6
d1cmbb_	d1cmwa1	d1co4a_	d1co7i_	d1coja1	d1cqta1	d1crea_	d1csb.1
d1cuna1	d1cuna2	d1cunb2	d1cunc2	d1cyca_	d1d0da_	d1d4ca1	d1d4da1
d1d5qa_	d1d5yb1	d1d6ba_	d1dd3a1	d1dd4b1	d1ddna2	d1deeg_	d1deeh_
d1dena_	d1dfaa2	d1dfna_	d1dkca_	d1dkya1	d1dkyb1	d1dlpb2	d1dp3a_
d1dprb1	d1dtka_	d1dtxa_	d1du0a_	d1du0b_	d1du2a_	d1du6a_	d1du9a_
d1dvba2	d1dw9a2	d1dwka2	d1dwma_	d1dx7a_	d1dx8a_	d1e08d_	d1e0ab_
d1e0la_	d1e0na_	d1e1qg_	d1e2aa_	d1e3ha1	d1e3oc1	d1e3pa1	d1e4qa_
d1e52a_	d1e6na1	d1e6pa1	d1e6ra1	d1e6za1	d1e7da1	d1e7la1	d1eaka1
d1ecma_	d1ecmb_	d1edia_	d1edja_	d1edka_	d1edla_	d1ef4a_	d1efea_
d1egfa_	d1ejmb_	d1en7a1	d1enha_	d1enka_	d1eqzd_	d1eqzh_	d1erca_
d1esxa_	d1ev0a_	d1ez3a_	d1ezvh_	d1ezvi_	d1ezzd2	d1f2ig2	d1f2ih2
d1f2ij2	d1f43a_	d1f4ia_	d1f5ri_	d1f5ta2	d1f5td1	d1f66b_	d1f66f_
d1fafa_	d1faki_	d1fana_	d1fbra1	d1fewa_	d1fexa_	d1fh1a_	d1fjgr_
d1fjla_	d1fjlb_	d1fjlc_	d1fjna_	d1fo1a2	d1fpoa2	d1fqva1	d1fs2a1
d1ftta_	d1ftza_	d1fx0b1	d1fx7a2	d1fy8i_	d1g2c.1	d1g2ha_	d1g3sa2
d1g6xa_	d1g73a_	d1gaba_	d1gata_	d1gaua_	d1gaxb4	d1 dta1	d1gjsa_
d1gjza_	d1gn2a1	d1gn3a1	d1gn4a1	d1gn6a1	d1gotg_	d1gp9a2	d1gpfa1
d1gt0c1	d1gt0d_	d1guua_	d1gv2a1	d1gv2a2	d1gv5a_	d1gvda_	d1gvna_
d1h0ia1	d1h0ta_	d1h3ha_	d1h3ob_	d1h5oa_	d1h88c1	d1h88c2	d1h88c3
d1h89c3	d1h8ac1	d1h8ac2	d1h8hg_	d1h8t.1	d1h9ta1	d1ha8a_	d1hbwa_
d1hcia4	d1hcib4	d1hcra_	d1hd6a_	d1hddc_	d1hdpa_	d1hf0a1	d1hf8a1
d1hg2a1	d1hg5a1	d1hg7a_	d1hi7b_	d1hlqa_	d1hlva2	d1hlya_	d1hmaa_
d1hmfa_	d1hn6a_	d1hnwr_	d1hnwt_	d1hnxn_	d1hnxr_	d1hnxt_	d1hnzr_
d1homa_	d1hp8a_	d1hq3d_	d1hr0r_	d1hr0t_	d1hrya_	d1hrza_	d1hsma_
d1htya1	d1huc.1	d1hwwa1	d1hxka1	d1i11a_	d1i1ga1	d1i3qi1	d1i5hw_
d1i6ca_	d1i6hi2	d1i6za_	d1i8gb_	d1i94t_	d1ibkr_	d1ibkt_	d1iblr_
d1ibmt_	d1ic8a1	d1icaa_	d1icfi_	d1icoa_	d1id3b_	d1id3f_	d1idsa1
d1idza_	d1ieta_	d1ifya_	d1ig7a_	d1igla_	d1ihra_	d1iiea_	d1iioa_
d1imxa_	d1irza_	d1itya_	d1iufa1	d1iufa2	d1iura_	d1iv6a_	d1ivsa1
d1iyma_	d1j1dc_	d1j1ec_	d1j3ca_	d1j3da_	d1j3xa_	d1j46a_	d1j47a_
d1j5et_	d1j5na_	d1j78a3	d1j7ea3	d1j95a_	d1jbda_	d1jc6a_	d1jcha3
d1jfia_	d1jgga_	d1jj2t_	d1jj2u_	d1jj6c_	d1jj8c_	d1jjra_	d1jk1a1
d1jkoc_	d1jkpc_	d1jkrc_	d1jm7a_	d1jmla_	d1jn7a_	d1joya_	d1jt6b1
d1jv8a_	d1jv9a_	d1jvma_	d1jvmb_	d1jvmd_	d1jxca_	d1jy2o_	d1jy3o_

Continued on Next Page...

Domain Identifiers

d1jy6b_	d1jyba2	d1k1va_	d1k4cc_	d1k4dc_	d1k61a_	d1k61c_	d1k6ua_
d1k73w_	d1k78a1	d1k78i1	d1k8aw_	d1k99a_	d1k9mw_	d1kb9f_	d1kb9i_
d1kbhb_	d1kc4a_	d1kc8w_	d1kd1w_	d1kdxa_	d1kigi_	d1kj5a_	d1kkca1
d1knpa1	d1knra1	d1knta_	d1kqsu_	d1krl.1	d1kssa1	d1ktha_	d1ktxa_
d1ku3a_	d1ku5a_	d1kuna_	d1kvd.1	d1kve.1	d1kw2a3	d1kx3b_	d1kx3f_
d1kx4f_	d1kx5b_	d1kxpd3	d1kyof_	d1kyoi_	d1kyot_	d1l0lj_	d1l0nh_
d1l0oc_	d1l3ha_	d1l3ya_	d1l4ab_	d1l4ta_	d1l6kc_	d1l6l1_	d1l8ya_
d1lbua1	d1lcca_	d1lcda_	d1ld5a_	d1ldkc_	d1ldkd2	d1ldke1	d1le8a_
d1lg4a_	d1lira_	d1lj2a_	d1lj2b_	d1lkma2	d1lkoa2	d1lkpa2	d1llmc1
d1lota3	d1lp1a_	d1lqca_	d1lrea_	d1lt9d_	d1ltjd_	d1lujb_	d1luvb1
d1lwma_	d1lwua_	d1lwue2	d1lwuh2	d1m18b_	d1m18f_	d1m19b_	d1m19f_
d1m1af_	d1m1ja_	d1m1jc2	d1m1kw_	d1m2sa_	d1m36a_	d1m5ia_	d1m62a_
d1m8la_	d1m90w_	d1ma1a1	d1ma9a3	d1mbea_	d1mbfa_	d1mbga_	d1mbha_
d1mbka_	d1mdma1	d1mg1a2	d1mh3a1	d1mh4a1	d1mjkb_	d1mjlb_	d1mjqd_
d1mm0a_	d1mmaa1	d1mn3a_	d1mntb_	d1msda1	d1msec1	d1msec2	d1msfc1
d1mt1.1	d1mt1.3	d1mt1.4	d1mt1.5	d1mtnd_	d1mtxa_	d1n0ja1	d1n13.1
d1n13.4	d1n13.5	d1n13.6	d1n1jb_	d1n32r_	d1n32t_	d1n33c1	d1n33s_
d1n86e2	d1n8la_	d1n8ma_	d1n8r2_	d1n8rw_	d1n98.1	d1naga_	d1nbmg_
d1nf7a3	d1ngmb_	d1ngmj_	d1ngmn_	d1nh2b_	d1nh2c_	d1nh2d2	d1nhma_
d1njiw_	d1njqa_	d1nk2p_	d1nk3p_	d1no1a_	d1nrea_	d1ns1a_	d1ntkj_
d1ntmh_	d1ntmj_	d1ntmk_	d1ntzh_	d1ntzk_	d1nu1h_	d1nu1k_	d1nu7d1
d1nvma1	d1nvob_	d1nvpc_	d1nysd_	d1o3xa_	d1o4xa1	d1o4xb_	d1o6ia1
d1o6wa1	d1o7zb_	d1o9aa1	d1oa55_	d1oa65_	d1oaia_	d1oawa_	d1occh_
d1ocoj_	d1ocow_	d1ocpa_	d1ocrb2	d1ocrh_	d1ocri_	d1ocru_	d1ocrv_
d1oczh_	d1oczw_	d1ofcx1	d1ogga1	d1ohhg_	d1ohzb_	d1olma1	d1on1a1
d1osla_	d1ov2a_	d1ov3a1	d1ov9a_	d1ov9b_	d1p2ha1	d1p2ii_	d1p2ji_
d1p2mb_	d1p2nb_	d1p2ob_	d1p2qb_	d1p34b_	d1p3ab_	d1p3bb_	d1p3bf_
d1p3gb_	d1p3gf_	d1p3ib_	d1p3kb_	d1p3lb_	d1p3lf_	d1p3mb_	d1p3mf_
d1p3of_	d1p3pb_	d1p47a1	d1p7ga1	d1p7ia_	d1p7ib_	d1p7ic_	d1p7id_
d1p7pa3	d1p84i_	d1p94a_	d1p9qc1	d1p9za_	d1pcna2	d1pgya_	d1pina1
d1pita_	d1pjua1	d1pjva_	d1pl4b1	d1pnha_	d1pnsr_	d1pnst_	d1pnxr_
d1po1.1	d1poga_	d1pon.1	d1pp9h_	d1ppjh_	d1prba_	d1prua_	d1prva_
d1pufa_	d1puoa2	d1pv4a1	d1pvoa1	d1q02a_	d1q1va_	d1q2ka_	d1q2na_
d1q7yw_	d1q812_	d1q81w_	d1q82w_	d1q86w_	d1qa6a_	d1qcva_	d1qgwa_
d1qkha_	d1qkya_	d1qlia2	d1qlqa_	d1qnma1	d1qo6a2	d1qo8a1	d1qp8b2
d1qrya_	d1qsda_	d1qu1a_	d1qu1e_	d1qu1f_	d1quza_	d1qvfu_	d1qvgu_
d1qwna1	d1qwua1	d1qx1a1	d1qyba2	d1qzea1	d1qzpa_	d1r0bi2	d1r1ga_
d1r3jc_	d1r3kc_	d1r3lc_	d1r4ag_	d1r4ga_	d1r73a_	d1rdga_	d1rdva_
d1rf8b_	d1ri7a1	d1rika_	d1rj9b1	d1rk4a2	d1rkwb1	d1rmda1	d1rofa_
d1rp3a3	d1rp3c3	d1rp3e3	d1rrza_	d1rsoa_	d1rsob_	d1rsod_	d1rwsa_
d1rzih2	d1s0yb_	d1s1cx_	d1s1ho_	d1s24a_	d1s2za2	d1s30a2	d1s32b_
d1s35a1	d1s5hc_	d1s72v_	d1s94a_	d1s94b_	d1sa0e_	d1sana_	d1sb0a_
d1sfkb_	d1sfke_	d1sfva_	d1shpa_	d1shyb2	d1skza1	d1smyf1	d1smyf2
d1sn9c_	d1snea_	d1soza1	d1ss1a_	d1sssa1	d1sxma_	d1t50a_	d1t6oa_
d1tafa_	d1taqa1	d1tawb_	d1tbgg_	d1tbgh_	d1tc3c_	d1tf0b_	d1tf3a3
d1tjla1	d1tocr1	d1tpai_	d1tpga2	d1trla_	d1ttea1	d1txma_	d1tzyd_
d1u78a2	d1ubdc1	d1ucsa_	d1ud0a_	d1ug2a_	d1ugla_	d1uhaa1	d1uhsa_

Continued on Next Page…

Table 1 – Continued

Domain Identifiers							
d1ujwb_	d1uk5a_	d1umqa_	d1unca_	d1unda_	d1uptb_	d1upth_	d1ur9a1
d1uuaa_	d1uuba_	d1uura1	d1uusa1	d1uxca_	d1uzca_	d1v54h_	d1v54i_
d1v55h_	d1v55i_	d1v55v_	d1v6ga2	d1vf6c_	d1vf6d_	d1vh6b_	d1vnda_
d1w0aa_	d1w0ba_	d1w0kg_	d1w2bu_	d1wdfa_	d1wdfb_	d1wdga_	d1wjfa_
d1wo9a_	d1xbla_	d1yrna_	d1ytfc_	d1ytfd2	d1yuja_	d1zeia_	d1zioa2
d1zwaa_	d2a3da_	d2a8va1	d2bcch_	d2bccj_	d2cbha_	d2cdxa_	d2cpba_
d2dtra2	d2e2aa_	d2eboa_	d2eiaa1	d2erla_	d2glia4	d2hdda_	d2hexa_
d2hipa_	d2hoaa_	d2hp8a_	d2kaii_	d2knta_	d2lefa_	d2lfba_	d2msia_
d2occh_	d2occi_	d2occu_	d2occv_	d2pspa2	d2rdva_	d2spga_	d2spza_
d2trcg_	d3bbga_	d3bcch_	d3btei_	d3btfi_	d3btgi_	d3bthi_	d3btki_
d3btqi_	d3btti_	d3btwi_	d3ci2a_	d3egfa_	d3gata_	d3hdda_	d3lria_
d3msia_	d3sdpa1	d3tgji_	d3tgki_	d3tpii_	d4msia_	d4ptia_	d4sgbi_
