# Supplementary material for: Automatic structure classification of small proteins using random forest
Source: BMC Bioinformatics. 2010 Jul 1;11:364. doi: 10.1186/1471-2105-11-364 (PMC2916923; doi:10.1186/1471-2105-11-364)
Supplement: Additional file 5 — Domains consisting of 3SSEs from SCOP version 1.73. This file lists the identifiers for the 3SSEs containing domains from SCOP version 1.73. [file 1471-2105-11-364-S5.PDF]

Additional File 5

Domains consisting of 3SSEs from SCOP version 1.73

Table 1: Domains consisting of 3SSEs from SCOP version 1.73

Domain Identifiers							
d1a1fa1	d1a62a1	d1a6qa1	d1a7wa_	d1aaba_	d1aila_	d1aj3a_	d1akha_
d1ap6b1	d1aplc_	d1ar4a1	d1ar5a1	d1atya_	d1au7a1	d1avma1	d1avya_
d1b67a_	d1b67b_	d1b6wa_	d1b72a_	d1b8ia_	d1b8ib_	d1b9xb_	d1b9yb_
d1bbya_	d1bcch_	d1bccj_	d1be3h_	d1bfma_	d1bgyh_	d1bhaa_	d1bhba_
d1bl8a_	d1bpta_	d1br0a_	d1bs3a1	d1bsma1	d1bt8a1	d1bunb_	d1bw5a_
d1c1gc_	d1c6sa_	d1c7ya1	d1ca0d_	d1chua1	d1ckta_	d1cmbb_	d1co7i_
d1cqta1	d1cuna1	d1cuna2	d1cunb2	d1cunc2	d1cxzb_	d1cyca_	d1d0da_
d1dd3a1	d1dd4b1	d1ddna2	d1deeh_	d1dema_	d1dena_	d1dkya1	d1dkyb1
d1dprb1	d1dtxa_	d1du0a_	d1du0b_	d1du2a_	d1du6a_	d1dwma_	d1e08d_
d1e0na_	d1e15a1	d1e1qg_	d1e2aa_	d1e3ha1	d1e3oc1	d1e3pa1	d1e52a_
d1e6pa1	d1e6ra1	d1e6za1	d1e7da1	d1eawb_	d1ecma_	d1ecmb_	d1edia_
d1edka_	d1edla_	d1ef4a_	d1efub3	d1en7a1	d1enha_	d1enka_	d1erpa_
d1ev0a_	d1ez3a_	d1ezvh_	d1ezvi_	d1f2ig1	d1f2ik1	d1f43a_	d1f4ia_
d1f80f_	d1f8ab1	d1fafa_	d1faki_	d1fewa_	d1fexa_	d1fjgt_	d1fjla_
d1fjlc_	d1fpoa2	d1ftta_	d1ftza_	d1fx0b1	d1fx7a2	d1g2c.1	d1g2ha_
d1g3wa2	d1g73a_	d1gaxb4	d1 dta1	d1gjza_	d1gn2a1	d1gn3a1	d1gn4a1
d1goia1	d1gotg_	d1gpfa1	d1gpja1	d1gt0c1	d1gt0d_	d1guua_	d1gv2a1
d1gv5a_	d1gvda_	d1gvna_	d1h0ga1	d1h0ia1	d1h0ta_	d1h3ob_	d1h88c1
d1h88c3	d1h89c2	d1h89c3	d1h8ac1	d1h8ac2	d1h8hg_	d1h9ta1	d1hcia3
d1hcib4	d1hddc_	d1hdpa_	d1hf0a1	d1hf8a1	d1hfaa1	d1hg2a1	d1hg5a1
d1hlva2	d1hmaa_	d1hmea_	d1hmfa_	d1hnwt_	d1hnxt_	d1hnzt_	d1homa_
d1hr0t_	d1hrya_	d1hrza_	d1hsma_	d1htaa_	d1htya1	d1hwwa1	d1hxka1
d1i11a_	d1i1ga1	d1i5hw_	d1i6za_	d1i94t_	d1ibkr_	d1ibkt_	d1iblt_
d1ic8a1	d1icfi_	d1id3b_	d1id3f_	d1idsa1	d1idya_	d1idza_	d1ig7a_
d1iiea_	d1iioa_	d1ijwc_	d1irza_	d1itya_	d1iufa1	d1iufa2	d1iura_
d1ivsa1	d1iw7f1	d1ixrb1	d1j1dc_	d1j1ec_	d1j3ca_	d1j3da_	d1j3xa_
d1j47a_	d1j5et_	d1j5na_	d1j78a3	d1j7ea3	d1j95a_	d1jc6a_	d1jcha3
d1jgga_	d1jj2u_	d1jj6c_	d1jjra_	d1jm7a_	d1jmla_	d1joya_	d1jt6b1
d1jvma_	d1jvmb_	d1jvmd_	d1jy3p_	d1k1va_	d1k4cc_	d1k4dc_	d1k61a_
d1k6ya1	d1k73w_	d1k78a1	d1k78i1	d1k8aw_	d1k99a_	d1k9mw_	d1kb9f_
d1kc8w_	d1kd1w_	d1kdxa_	d1kkca1	d1kmhb1	d1knpa1	d1knra1	d1knta_
d1kqsu_	d1krl.1	d1ktha_	d1ku2a1	d1ku3a_	d1ku5a_	d1kuna_	d1kvd.1
d1kw2a3	d1kx3b_	d1kx3f_	d1kx4b_	d1kx4f_	d1kx5b_	d1kx7a_	d1kxpd3
d1kyoi_	d1kyot_	d1l0lj_	d1l0nh_	d1l0nj_	d1l0oc_	d1l4ab_	d1l6ha_
d1l6kc_	d1l6l1_	d1l8ya_	d1l8za_	d1lbua1	d1lcca_	d1lcda_	d1ld5a_
d1le8a_	d1lfba_	d1lg4a_	d1lgha_	d1lira_	d1lj2a_	d1lj2b_	d1llmc1
d1lp1a_	d1lq7a_	d1lqca_	d1lr1a_	d1lrea_	d1lt9d_	d1ltjd_	d1luvb1
d1lwma_	d1lwua_	d1lwue2	d1lwuh2	d1m18b_	d1m18f_	d1m19b_	d1m19f_
d1m1af_	d1m1ja_	d1m1jc2	d1m1kw_	d1m1pa_	d1m2sa_	d1m5ia_	d1m62a_
d1m8la_	d1m90w_	d1ma1a1	d1ma9a3	d1mbea_	d1mbfa_	d1mbga_	d1mbha_
d1mbka_	d1mdma1	d1mg1a2	d1mh3a1	d1mh4a1	d1mjea2	d1mjkb_	d1mjlb_
d1mm0a_	d1mn3a_	d1msda1	d1msec1	d1msec2	d1msfc1	d1msfc2	d1mt1.1

Continued on Next Page…

Table 1 – Continued

Domain Identifiers

d1mt1.4	d1mt1.5	d1mtnd_	d1n0ja1	d1n13.1	d1n13.3	d1n13.4	d1n13.5
d1n1jb_	d1n32r_	d1n32s_	d1n32t_	d1n33c1	d1n33s_	d1n33t_	d1n34t_
d1n8ma_	d1n8rw_	d1n98.1	d1naga_	d1nbmg_	d1ngmb_	d1ngmj_	d1nh2b_
d1nh2d2	d1nhma_	d1nhna_	d1ni8a_	d1njiw_	d1nk2p_	d1nk3p_	d1nl0g_
d1nrea_	d1ns1a_	d1ntkj_	d1ntkk_	d1ntmh_	d1ntmj_	d1ntmk_	d1ntzh_
d1nu1h_	d1nu1k_	d1nu7d1	d1nu9c1	d1nvma1	d1nvob_	d1nvpc_	d1nysd_
d1o4xa1	d1o4xb_	d1o6ia1	d1o6ua1	d1o6wa1	d1oa55_	d1oa65_	d1oaia_
d1ocoh_	d1ocoj_	d1ocow_	d1ocpa_	d1ocrb2	d1ocrh_	d1ocri_	d1ocru_
d1octc1	d1oczh_	d1oczw_	d1ofcx1	d1ogba1	d1ogga1	d1ohhg_	d1ohzb_
d1oksa_	d1olma1	d1on1a1	d1oo3a_	d1op1a_	d1osla_	d1otra_	d1ov2a_
d1ov9a_	d1ov9b_	d1ovxa_	d1p2ii_	d1p2ji_	d1p2ki_	d1p2mb_	d1p2nb_
d1p2qb_	d1p34b_	d1p3ab_	d1p3bb_	d1p3bf_	d1p3fb_	d1p3gb_	d1p3gf_
d1p3kb_	d1p3lb_	d1p3lf_	d1p3mb_	d1p3mf_	d1p3ob_	d1p3of_	d1p3pb_
d1p7ia_	d1p7ib_	d1p7ic_	d1p7id_	d1p7ja_	d1p84i_	d1p94a_	d1p9qc1
d1pina1	d1pira_	d1pita_	d1pjva_	d1pl4b1	d1pmxa_	d1pnha_	d1pnst_
d1poga_	d1pon.1	d1pp9h_	d1pp9j_	d1pp9w_	d1ppjh_	d1ppjw_	d1prba_
d1prva_	d1ps3a1	d1pufa_	d1puoa2	d1pv4a1	d1pvoa1	d1q1va_	d1q2na_
d1q81w_	d1q82w_	d1q86w_	d1q8ha_	d1qa6a_	d1qgwa_	d1qgwb_	d1qkha_
d1qlqa_	d1qnma1	d1qp8b2	d1qrva_	d1qrya_	d1qsda_	d1qu1a_	d1qu1e_
d1qvfu_	d1qvgu_	d1qvhh_	d1qvub1	d1qwna1	d1qwua1	d1qx1a1	d1qzea1
d1r0bi2	d1r1ga_	d1r33a1	d1r34a1	d1r3ic_	d1r3jc_	d1r3kc_	d1r3lc_
d1r4ag_	d1r4ga_	d1r73a_	d1re4d_	d1resa_	d1reta_	d1rf8b_	d1ri7a1
d1rk4a2	d1rkwb1	d1rm1b2	d1rp3a1	d1rp3a3	d1rp3c3	d1rp3e3	d1rp3g1
d1rq6a_	d1rqva1	d1rrza_	d1rsoa_	d1rsob_	d1rsod_	d1s0yb_	d1s1cx_
d1s32b_	d1s32f_	d1s35a1	d1s5hc_	d1s72v_	d1s7ea1	d1s94a_	d1s94b_
d1sana_	d1sb0a_	d1sc5a1	d1scya_	d1sddb1	d1se7a_	d1sfkb_	d1sfke_
d1shpa_	d1slma1	d1smyf1	d1smyf2	d1smyp2	d1sn9c_	d1sp4.1	d1sqbh_
d1sqph1	d1sqpj1	d1sqqh1	d1sqqj1	d1sqvh1	d1sqvj1	d1sqxh1	d1sqxj1
d1ss1a_	d1su3a1	d1t6oa_	d1t7cb1	d1t8ia1	d1t8lb1	d1t8mb1	d1t8nb1
d1t95a1	d1tafa_	d1tapa_	d1tawb_	d1tbge_	d1tbgg_	d1tbgh_	d1tc3c_
d1tf0b_	d1tf3a3	d1tf6a3	d1tfxc_	d1tjla1	d1tl8a1	d1tocr2	d1tocs2
d1tocu2	d1toza1	d1tpai_	d1tqsa1	d1tqta1	d1tqua1	d1tqva1	d1tqwa1
d1ttea1	d1txma_	d1tzyd_	d1u35b1	d1u4qa2	d1u4qa3	d1u5pa1	d1u5pa2
d1u78a1	d1u78a2	d1u7mb1	d1u85a1	d1u8cb6	d1u8rb2	d1ud0a_	d1ug2a_
d1uj8a1	d1ujwb_	d1uk5a_	d1umqa_	d1unda_	d1uptb_	d1uptd_	d1upth_
d1ur9a1	d1ut3a_	d1uuaa_	d1uuba_	d1uura1	d1uusa1	d1uxca_	d1uzca_
d1v54i_	d1v54v_	d1v55h_	d1v55i_	d1v55v_	d1v92a_	d1vcta1	d1vega_
d1veka_	d1vf6a_	d1vf6c_	d1vf6d_	d1vf9a1	d1vfca1	d1vh6b_	d1vnda_
d1voqt_	d1vosj_	d1vost_	d1vovj_	d1vovt_	d1voxj_	d1voxt_	d1vozj_
d1vq4i1	d1vq4v1	d1vq5v1	d1vq6v1	d1vq7i1	d1vq7v1	d1vq8v1	d1vq9v1
d1vqlv1	d1vqmv1	d1vqnv1	d1vqov1	d1vqpv1	d1vrya1	d1vyxa_	d1w09a_
d1w0ba_	d1w0kg_	d1w0ta_	d1w0ua_	d1w2bu_	d1wa8a1	d1wa8b1	d1wb7a1
d1wdfa_	d1wdfb_	d1wdga_	d1wfda_	d1wgla_	d1wgxa_	d1wh5a_	d1wh7a_
d1whua_	d1wi3a_	d1wiva_	d1wjfa_	d1wjia_	d1wmta_	d1wncf_	d1wpaa1
d1wr1b1	d1wsuc2	d1wt7a_	d1x2ma1	d1x2na1	d1x3ha2	d1x41a1	d1x58a1
d1x6ea1	d1x6fa1	d1x9ba_	d1xawa1	d1xb2b1	d1xbla_	d1xc5a1	d1xcva2
d1xeqa1	d1xf6a_	d1xf6b_	d1xf7a_	d1xg0a_	d1xg0b_	d1xg1a1	d1xilb1

Continued on Next Page. . .

Table 1 – Continued

**Domain Identifiers**							
d1xmot_	d1xmqr_	d1xmqt_	d1xnqr_	d1xnqt_	d1xnrr_	d1xnrt_	d1xoub_
d1xprb1	d1xrza_	d1xt3b_	d1xu2r_	d1xvha2	d1xzya_	d1y01a_	d1y43.1
d1y74b1	d1y74d1	d1y76a1	d1y9ba1	d1ybza1	d1yhqv1	d1yi2v1	d1yijv1
d1yitv1	d1yj9v1	d1yjni1	d1yjnv1	d1yjwv1	d1ykea1	d1yked1	d1ykha1
d1yktb1	d1yl3i1	d1yl3j1	d1yl4u1	d1yl4v1	d1yl4w1	d1ylaa1	d1yrna_
d1ytfd2	d1ytzi1	d1yu8x1	d1yxra1	d1yz8p1	d1z8ua1	d1zaac1	d1zaea1
d1zjdb1	d1zk8b1	d1zkea1	d1zl8a1	d1zl8b1	d1zlab1	d1zoqc1	d1zpya1
d1zr2a1	d1zspa1	d1zteb1	d1zuqa1	d1zwaa_	d1zx3a1	d1zyrf1	d2a06h1
d2a3da_	d2a68f1	d2a69f1	d2a6ef1	d2a6hf1	d2a8va1	d2ae9a1	d2aghb1
d2alwa1	d2arod1	d2awbx1	d2az2b1	d2b63d1	d2b64t1	d2b66k1	d2b8kd1
d2b9nk1	d2b9ot1	d2b9za1	d2bbja2	d2bcch_	d2bccj_	d2be5f1	d2bkna1
d2bkpa1	d2bnka1	d2bobc1	d2bocc1	d2bska1	d2bsqe1	d2bsqf1	d2bsqh1
d2bwea1	d2c2aa1	d2c5ra1	d2cfxa1	d2cg4a1	d2cjja1	d2cnwd1	d2cnwf1
d2cosa1	d2cota1	d2cp9a1	d2cpba_	d2cpta1	d2cpwa1	d2cqqa1	d2cqra1
d2craa1	d2crba1	d2crda_	d2crga1	d2csha2	d2ct1a2	d2cu7a1	d2cuea1
d2cv5b1	d2cw0f1	d2cw0f2	d2cyya1	d2d3ea1	d2d8da1	d2d8ea1	d2daha1
d2df4c1	d2di0a1	d2diia1	d2dkla1	d2dlka2	d2dqnc1	d2drpa2	d2ds5a1
d2ds8a1	d2dtra2	d2dwdc1	d2dwec1	d2dyrh1	d2dyri1	d2dyrv1	d2dysh1
d2dysv1	d2e1oa1	d2e2aa_	d2e5lr1	d2e5lt1	d2e7sc1	d2e7sh1	d2e7sq1
d2eboa_	d2ecca1	d2eiaa1	d2eijh1	d2eiji1	d2eijv1	d2eikh1	d2eiki1
d2eilh1	d2eili1	d2eilv1	d2eimh1	d2eimi1	d2eimv1	d2einh1	d2eini1
d2ev0a1	d2ev5a1	d2ev6b1	d2ezwa1	d2f18a1	d2f1aa1	d2f1ba1	d2f21a1
d2f4vt1	d2f5da1	d2f5ea1	d2f7oa1	d2f7pa1	d2f7qa1	d2f7ra1	d2f8nb1
d2fd5a1	d2fhsc1	d2fj7b1	d2ftua1	d2fyla1	d2fyva1	d2fzta1	d2g42a1
d2gata_	d2gboa1	d2 dsc1	d2gf1a_	d2gm4a1	d2gyaw1	d2gyc31	d2gycw1
d2gzwb1	d2h9ca1	d2h9da1	d2hdda_	d2hexa_	d2hgha1	d2hgiu1	d2hgiv1
d2hgj81	d2hgpu1	d2hgpv1	d2hgpw1	d2hgq81	d2hgru1	d2hgrv1	d2hgrw1
d2hhht1	d2hi3a1	d2hiod_	d2hjfc1	d2hn2a2	d2hoaa_	d2hodg1	d2hp8a_
d2huec1	d2hvjc1	d2hvkc1	d2hyfb1	d2ibzh1	d2ibzi1	d2idob1	d2ijoi1
d2iszb2	d2itcc1	d2itdc1	d2itka1	d2iu7a2	d2iuoa2	d2iyld1	d2j00t1
d2j02t1	d2j28x1	d2j7pd1	d2j7pe1	d2ja5d1	d2ja6d1	d2ja7d1	d2ja8d1
d2knta_	d2ktxa_	d2lefa_	d2lfba_	d2np3a1	d2nvqi1	d2nvxj1	d2nzdb1
d2oaua3	d2occb2	d2occh_	d2occi_	d2occu_	d2occv_	d2otjv1	d2otlv1
d2ptaa_	d2ptci_	d2q5aa1	d2spza_	d2tgpi_	d2tpii_	d2trcg_	d2uu9t1
d2uuat1	d2uubt1	d2uuct1	d2uxct1	d3bcch_	d3bccj_	d3btdi_	d3btei_
d3btgi_	d3bthi_	d3btki_	d3btmi_	d3btqi_	d3btti_	d3btwi_	d3gata_
d3lria_	d3mon.5	d3sdpa1	d3tgii_	d3tgji_	d3tgki_	d3tpii_	d4ptia_
d5ptia_	d5znfa_	d6inse_	d6paxa2	d6ptia_	d7ptia_	d7znfa_	d8ptia_
d9ptia_	d1a0aa_	d1a0rg_	d1a1fa1	d1a1ga2	d1a1ha1	d1a1ia2	d1a1ka1
d1a5ja1	d1a62a1	d1a63a1	d1a6qa1	d1a7ia2	d1a7wa_	d1a8va1	d1aaba_
d1aapa_	d1aaya1	d1adza_	d1af8a_	d1ahdp_	d1aila_	d1aipc1	d1aj3a_
d1an4b_	d1aoib_	d1aoif_	d1ap5b1	d1ap6b1	d1aplc_	d1ar4a1	d1ar5a1
d1au7a1	d1avma1	d1avya_	d1avyb_	d1ayga_	d1azka_	d1b06a1	d1b0ca_
d1b50a_	d1b67a_	d1b67b_	d1b6wa_	d1b71a2	d1b72a_	d1b8ia_	d1b8ib_
d1b9xb_	d1b9yb_	d1ba5a_	d1bbya_	d1bcch_	d1bccj_	d1bdca_	d1bdda_
d1be3h_	d1bf0a_	d1bfma_	d1bg5a2	d1bgka_	d1bgyh_	d1bhba_	d1bhca_
d1bhia_	d1bika1	d1bl8a_	d1boea_	d1bpia_	d1bpta_	d1bq0a_	d1bq8a_

Continued on Next Page…

Table 1 – Continued

Domain Identifiers

d1br0a_	d1brbi_	d1brfa_	d1bs3a1	d1bsma1	d1bt8a1	d1bthp_	d1btia_
d1busa_	d1bw5a_	d1bz5a_	d1bzxi_	d1c04c_	d1c0wc2	d1c17a_	d1c1gc_
d1c6sa_	d1c7ya1	d1ca0d_	d1caaa_	d1cada_	d1cbwd_	d1chla_	d1chua1
d1ck7a6	d1ckta_	d1cmbb_	d1cmwa1	d1co7i_	d1coja1	d1cqta1	d1csb.1
d1cuna1	d1cuna2	d1cunb2	d1cunc2	d1cyca_	d1d0da_	d1d4da1	d1d4ea1
d1d5yb1	d1d6ba_	d1dd3a1	d1dd4b1	d1ddna2	d1deeg_	d1deeh_	d1dema_
d1dfaa2	d1dfna_	d1dkca_	d1dkya1	d1dkyb1	d1dlpb2	d1dp3a_	d1dpra2
d1dtxa_	d1du0a_	d1du0b_	d1du2a_	d1du6a_	d1dv0a_	d1dv5a_	d1dvba2
d1dwka2	d1dwma_	d1dx7a_	d1e08d_	d1e0ab_	d1e0ha_	d1e0na_	d1e1qg_
d1e3ha1	d1e3oc1	d1e3pa1	d1e4qa_	d1e52a_	d1e6na1	d1e7da1	d1e7la1
d1eawb_	d1ecma_	d1ecmb_	d1edia_	d1edja_	d1edka_	d1edla_	d1ef4a_
d1efub3	d1egfa_	d1ejmb_	d1en7a1	d1enha_	d1enka_	d1eqzd_	d1eqzh_
d1erpa_	d1esxa_	d1ev0a_	d1ez3a_	d1ezvh_	d1ezvi_	d1ezzd2	d1f2ig1
d1f2ih2	d1f2ii2	d1f2ij2	d1f2ik1	d1f2ik2	d1f43a_	d1f4ia_	d1f5ri_
d1f5td1	d1f66b_	d1f66f_	d1f7zi_	d1fafa_	d1faki_	d1fana_	d1fbra1
d1fexa_	d1fjgr_	d1fjgt_	d1fjla_	d1fjlb_	d1fjlc_	d1fpoa2	d1fqva1
d1fsef_	d1ftta_	d1ftza_	d1fx0b1	d1fx7a2	d1fy8i_	d1g2c.1	d1g2ha_
d1g3wa2	d1g6xa_	d1g73a_	d1gaba_	d1gata_	d1gaua_	d1gaxb4	d1 dta1
d1gjta_	d1gjza_	d1gn2a1	d1gn3a1	d1gn4a1	d1gn6a1	d1gotg_	d1gp9a2
d1gpja1	d1gt0c1	d1gt0d_	d1guua_	d1gv2a1	d1gv2a2	d1gv5a_	d1gvda_
d1gzrb_	d1gzzb_	d1h02b_	d1h0ta_	d1h3ha_	d1h3ob_	d1h59a_	d1h88c1
d1h88c3	d1h89c2	d1h89c3	d1h8ac1	d1h8ac2	d1h8hg_	d1h9ta1	d1hbwa_
d1hcia4	d1hcib4	d1hcra_	d1hd6a_	d1hddc_	d1hdpa_	d1hf0a1	d1hf8a1
d1hg2a1	d1hg5a1	d1hg7a_	d1hlqa_	d1hlva2	d1hlya_	d1hmaa_	d1hmea_
d1hn6a_	d1hnra_	d1hnwr_	d1hnwt_	d1hnxt_	d1hnzr_	d1hnzt_	d1homa_
d1hq3d_	d1hqba_	d1hr0r_	d1hr0t_	d1hrya_	d1hrza_	d1hsma_	d1htaa_
d1huc.1	d1hwwa1	d1hxka1	d1hypa_	d1i11a_	d1i1ga1	d1i5hw_	d1i5ka_
d1i94t_	d1ibkr_	d1ibkt_	d1iblr_	d1iblt_	d1ibmt_	d1ic8a1	d1icfi_
d1id3f_	d1idsa1	d1idya_	d1idza_	d1ieta_	d1ifya_	d1ig7a_	d1igla_
d1iiea_	d1iioa_	d1ijwc_	d1imxa_	d1irza_	d1itya_	d1iufa1	d1iufa2
d1iv6a_	d1ivsa1	d1iw7f1	d1iyma_	d1izl0_	d1izlv_	d1j1dc_	d1j1ec_
d1j3da_	d1j3xa_	d1j46a_	d1j47a_	d1j5er_	d1j5et_	d1j5na_	d1j78a3
d1j95a_	d1jc6a_	d1jcha3	d1jeqa1	d1jfia_	d1jgga_	d1jj2u_	d1jj6c_
d1jjra_	d1jjsa_	d1jk1a1	d1jk2a2	d1jkoc_	d1jkpc_	d1jkrc_	d1jm7a_
d1jn7a_	d1joya_	d1jt6b1	d1jv8a_	d1jv9a_	d1jvma_	d1jvmb_	d1jvmd_
d1jy2o_	d1jy3o_	d1jy6b_	d1jyba2	d1k1va_	d1k4cc_	d1k4dc_	d1k61a_
d1k6ua_	d1k6ya1	d1k73w_	d1k78a1	d1k78i1	d1k81a_	d1k8av_	d1k8aw_
d1k9mw_	d1k9ra_	d1kb9f_	d1kb9i_	d1kbha_	d1kbhb_	d1kc4a_	d1kc8w_
d1kd1w_	d1kdxa_	d1kigi_	d1kkca1	d1kmhb1	d1knpa1	d1knra1	d1knta_
d1kqsu_	d1krl.1	d1kssa1	d1ktha_	d1ku2a1	d1ku3a_	d1ku5a_	d1kuna_
d1kve.1	d1kw2a3	d1kwoa1	d1kx3b_	d1kx3f_	d1kx4b_	d1kx4f_	d1kx5b_
d1kyof_	d1kyoi_	d1kyot_	d1l0nh_	d1l0nj_	d1l0oc_	d1l3ha_	d1l4ab_
d1l6ha_	d1l6kc_	d1l6l1_	d1l8cb_	d1l8ya_	d1l8za_	d1lbua1	d1lcca_
d1ld5a_	d1ldkc_	d1ldkd2	d1ldke1	d1le8a_	d1lfba_	d1lg4a_	d1lira_
d1lj2b_	d1lkma2	d1lkoa2	d1lkpa2	d1llmc1	d1lmja2	d1lota3	d1lp1a_
d1lrea_	d1lt9d_	d1ltjd_	d1lujb_	d1luvb1	d1lvfa_	d1lwma_	d1lwua_
d1lwuh2	d1m18b_	d1m18f_	d1m19b_	d1m19f_	d1m1ab_	d1m1af_	d1m1eb_

Continued on Next Page…

Domain Identifiers							
d1m1jc2	d1m1kw_	d1m1pa_	d1m1pb_	d1m1qa_	d1m1ra_	d1m2sa_	d1m36a_
d1m62a_	d1m7ka_	d1m8la_	d1m90w_	d1ma1a1	d1ma9a3	d1mbea_	d1mbfa_
d1mbha_	d1mbja_	d1mbka_	d1mdma1	d1mg1a2	d1mh3a1	d1mh4a1	d1mjkb_
d1mjqd_	d1mkna_	d1mm0a_	d1mmaa1	d1mn3a_	d1mntb_	d1msda1	d1msec1
d1msfc1	d1msfc2	d1mt1.1	d1mt1.3	d1mt1.4	d1mt1.5	d1mtnd_	d1mtxa_
d1n13.1	d1n13.3	d1n13.4	d1n13.5	d1n13.6	d1n1jb_	d1n32r_	d1n32t_
d1n33s_	d1n36s_	d1n36t_	d1n86e2	d1n8la_	d1n8ma_	d1n8rv_	d1n8rw_
d1naga_	d1nbmg_	d1neia_	d1nf7a3	d1ngmb_	d1ngmj_	d1ngmn_	d1nh2b_
d1nh2d2	d1nhma_	d1nhna_	d1njiw_	d1njqa_	d1nk2p_	d1nk3p_	d1no1a_
d1ns1a_	d1ntkj_	d1ntkk_	d1ntmh_	d1ntmj_	d1ntmk_	d1ntzh_	d1ntzk_
d1nu1k_	d1nu7d1	d1nu9c1	d1nvma1	d1nvob_	d1nvpc_	d1nysd_	d1o3xa_
d1o4xb_	d1o6ia1	d1o6ua1	d1o6wa1	d1o7zb_	d1o9aa1	d1oa55_	d1oa65_
d1occh_	d1ocoh_	d1ocoj_	d1ocow_	d1ocpa_	d1ocrb2	d1ocrh_	d1ocri_
d1ocrv_	d1octc1	d1oczh_	d1oczw_	d1ofcx1	d1ohhg_	d1ohzb_	d1okkd1
d1on1a1	d1oo3a_	d1op1a_	d1oqdl_	d1osla_	d1ov2a_	d1ov3a1	d1ov9a_
d1p2ii_	d1p2ji_	d1p2ki_	d1p2mb_	d1p2nb_	d1p2ob_	d1p2qb_	d1p34b_
d1p3bb_	d1p3bf_	d1p3fb_	d1p3gb_	d1p3gf_	d1p3ib_	d1p3kb_	d1p3lb_
d1p3mb_	d1p3mf_	d1p3ob_	d1p3of_	d1p3pb_	d1p7ga1	d1p7ia_	d1p7ib_
d1p7id_	d1p7ja_	d1p84i_	d1p94a_	d1p9qc1	d1pgya_	d1pira_	d1pita_
d1pjva_	d1pl4b1	d1pmxa_	d1pnha_	d1pnsr_	d1pnst_	d1pnxr_	d1pnxt_
d1poga_	d1pon.1	d1pp9h_	d1ppjh_	d1prba_	d1prua_	d1prva_	d1ps3a1
d1puoa2	d1pv4a1	d1pvoa1	d1q02a_	d1q1va_	d1q2ka_	d1q2na_	d1q7yw_
d1q81v_	d1q81w_	d1q82w_	d1q86w_	d1q8ha_	d1q9ia1	d1qa6a_	d1qcva_
d1qgwb_	d1qkha_	d1qkya_	d1qlia2	d1qlqa_	d1qnma1	d1qo6a2	d1qo8a1
d1qrva_	d1qrya_	d1qsda_	d1qu1a_	d1qu1e_	d1qu1f_	d1qvfu_	d1qvgu_
d1qwna1	d1qwua1	d1qx1a1	d1qyba2	d1qzea1	d1qzpa_	d1r1ga_	d1r33a1
d1r3ic_	d1r3jc_	d1r3kc_	d1r3lc_	d1r4ag_	d1r4ga_	d1r73a_	d1rdva_
d1resa_	d1rf8b_	d1ri7a1	d1rika_	d1rj9b1	d1rk4a2	d1rkwb1	d1rm1b2
d1rp3a1	d1rp3a3	d1rp3c3	d1rp3e3	d1rp3g1	d1rq6a_	d1rrza_	d1rsoa_
d1rsod_	d1rwsa_	d1ryta2	d1s0yb_	d1s1cx_	d1s1ho_	d1s2za2	d1s30a2
d1s32f_	d1s35a1	d1s5hc_	d1s72v_	d1s7ea1	d1s94a_	d1s94b_	d1sa0e_
d1sb0a_	d1sc5a1	d1scya_	d1sddb1	d1se7a_	d1sfkb_	d1sfke_	d1sfva_
d1shyb2	d1slma1	d1smyf1	d1smyf2	d1smyp2	d1sn9c_	d1snab_	d1snea_
d1sqbh_	d1sqph1	d1sqpj1	d1sqqh1	d1sqvh1	d1sqxh1	d1sr9a1	d1ss1a_
d1su3a1	d1t6oa_	d1t7cb1	d1t8ia1	d1t8lb1	d1t8mb1	d1t8nb1	d1t8ob1
d1t95a1	d1tafa_	d1tapa_	d1taqa1	d1tawb_	d1tbge_	d1tbgg_	d1tbgh_
d1tdha3	d1tf0b_	d1tf3a1	d1tf3a2	d1tf3a3	d1tf6a3	d1tfxc_	d1tjla1
d1tocr1	d1tocr2	d1tocs2	d1toct2	d1tocu2	d1tpai_	d1tqsa1	d1tqta1
d1tqva1	d1tqwa1	d1trla_	d1ttea1	d1txaa_	d1txma_	d1tzyd_	d1u35b1
d1u4qa3	d1u5pa1	d1u5pa2	d1u5td2	d1u78a1	d1u78a2	d1u7mb1	d1u85a1
d1u8cb6	d1u8rb2	d1ubdc1	d1ud0a_	d1ug2a_	d1uhaa1	d1uhsa_	d1ujsa_
d1uk5a_	d1unca_	d1unda_	d1uptb_	d1upth_	d1ut3a_	d1uuaa_	d1uuba_
d1uusa1	d1uxca_	d1uxda_	d1uzca_	d1v54h_	d1v54i_	d1v54v_	d1v55h_
d1v55v_	d1va2a1	d1vcta1	d1vcxa_	d1vega_	d1veka_	d1vf6a_	d1vf6c_
d1vf9a1	d1vfca1	d1vh6b_	d1viia_	d1vk6a4	d1vnda_	d1voqh_	d1voqo_
d1vosh_	d1voso_	d1voss_	d1vovh_	d1vovo_	d1vovs_	d1voxh_	d1voxo_
d1vozh_	d1vozo_	d1vozs_	d1vq4i1	d1vq4v1	d1vq5v1	d1vq611	d1vq6v1

Continued on Next Page. . .

Table 1 – Continued

Domain Identifiers

d1vq7v1	d1vq8v1	d1vq9v1	d1vqkv1	d1vqlv1	d1vqmv1	d1vqnv1	d1vqov1
d1vrya1	d1w09a_	d1w0aa_	d1w0ba_	d1w0kg_	d1w0ta_	d1w0ua_	d1w2bu_
d1wa8b1	d1wb7a1	d1wb8a1	d1wdfa_	d1wdfb_	d1wdga_	d1wfda_	d1wgla_
d1wh5a_	d1wh7a_	d1whca_	d1whua_	d1wi3a_	d1wiva_	d1wjfa_	d1wncf_
d1wr0a1	d1wr1b1	d1wsuc2	d1wt7a_	d1wvka_	d1wyha1	d1x2ma1	d1x2na1
d1x4ka1	d1x4la2	d1x58a1	d1x64a2	d1x6ea2	d1x9ba_	d1xawa1	d1xb2b1
d1xbta2	d1xc5a1	d1xcva2	d1xdca1	d1xeqa1	d1xf6a_	d1xf6b_	d1xf7a_
d1xg0b_	d1xg1a1	d1xilb1	d1xmot_	d1xmqt_	d1xnqt_	d1xnrr_	d1xnrt_
d1xpaa2	d1xpoc1	d1xprb1	d1xpua1	d1xrza_	d1xu1r_	d1xvha2	d1xx6a2
d1y01a_	d1y0ja1	d1y0pa1	d1y74a1	d1y74b1	d1y74d1	d1y76a1	d1y9ba1
d1yhqv1	d1yi2v1	d1yijv1	d1yiti1	d1yitv1	d1yj9v1	d1yjni1	d1yjnv1
d1ykea1	d1yked1	d1ykha1	d1ykhb1	d1yktb1	d1yl3d2	d1yl3i1	d1yl3j1
d1yl4w1	d1yrna_	d1ytfc_	d1ytfd2	d1ytzi1	d1yu8x1	d1yuja_	d1yxra1
d1z6jl3	d1z8ua1	d1zaac1	d1zaea1	d1zioa2	d1zjdb1	d1zk8b1	d1zkea1
d1zl8b1	d1zlab1	d1zpya1	d1zq3p1	d1zr2a1	d1zrpa_	d1zspa1	d1zteb1
d1zwaa_	d1zx3a1	d1zyrf1	d2a06h1	d2a06w1	d2a0fb2	d2a3da_	d2a68f1
d2a6ef1	d2a6hf1	d2ae9a1	d2aghb1	d2ahma1	d2akla2	d2alwa1	d2apob1
d2az2b1	d2b63d1	d2b64t1	d2b8kd1	d2b8ol3	d2b9mt1	d2b9ot1	d2b9za1
d2bccj_	d2be5f1	d2bkna1	d2bkoa1	d2bkpa1	d2bnka1	d2bocc1	d2bska1
d2bwea1	d2c2aa1	d2c5ra1	d2cbha_	d2cfxa1	d2cg4a1	d2cjja1	d2cnwd1
d2coba1	d2cora1	d2cosa1	d2cota1	d2cp9a1	d2cpba_	d2cpta1	d2cqqa1
d2craa1	d2crba1	d2crda_	d2crga1	d2ct1a1	d2cu7a1	d2cuea1	d2cufa1
d2cw0f1	d2cw0f2	d2cyya1	d2d3ea1	d2d8da1	d2d8ea1	d2daha1	d2dara2
d2di0a1	d2diia1	d2dj7a1	d2dk1a1	d2dkla1	d2dlka2	d2dloa2	d2dlqa1
d2dqnc1	d2drpa2	d2dsqc1	d2dtra2	d2dwdc1	d2dwec1	d2dyrh1	d2dyri1
d2dysh1	d2dysi1	d2dysv1	d2e1oa1	d2e2aa_	d2e5lt1	d2e7sc1	d2e7sh1
d2e7sr1	d2eboa_	d2ecca1	d2eiaa1	d2eijh1	d2eiji1	d2eijv1	d2eiki1
d2eili1	d2eilv1	d2eimi1	d2eimv1	d2einh1	d2eini1	d2einv1	d2erla_
d2ev5a1	d2ev6b1	d2f18a1	d2f1aa1	d2f1ba1	d2f4vt1	d2f5da1	d2f5ea1
d2f7pa1	d2f7qa1	d2f7ra1	d2f8nb1	d2fcwa1	d2fj7b1	d2ftua1	d2fyla1
d2fzta1	d2g42a1	d2gata_	d2gboa1	d2 dsc1	d2glia4	d2gm4a1	d2gw9a1
d2gyc31	d2gycw1	d2gzka1	d2gzwb1	d2h9ca1	d2h9da1	d2hdda_	d2hexa_
d2hgiv1	d2hgiw1	d2hgj81	d2hgpu1	d2hgpv1	d2hgq81	d2hgrv1	d2hgu81
d2hi3a1	d2hiod_	d2hipa_	d2hjfc1	d2hoaa_	d2hodg1	d2hp8a_	d2hpca1
d2hvjc1	d2hvkc1	d2hyfb1	d2i9aa2	d2ibzh1	d2ibzi1	d2ijoi1	d2io5c1
d2itdc1	d2iuoa2	d2iyld1	d2j00t1	d2j02r1	d2j02t1	d2j7pd1	d2j7pe1
d2jmqa1	d2knta_	d2lefa_	d2lfba_	d2nbta_	d2nvxi1	d2nvxj1	d2nzdb1
d2occh_	d2occi_	d2occu_	d2occv_	d2otlv1	d2p4ka1	d2rdva_	d2spza_
d2uu9t1	d2uuat1	d2uubt1	d2uuct1	d2uxct1	d3gata_	d3hdda_	d3lria_
d4ptia_	d4sgbi_	d4tpii_	d5gata_	d5znfa_	d6ptia_	d7amea_	d7rxna_
