# Supplementary material for: Automatic structure classification of small proteins using random forest
Source: BMC Bioinformatics. 2010 Jul 1;11:364. doi: 10.1186/1471-2105-11-364 (PMC2916923; doi:10.1186/1471-2105-11-364)
Supplement: Additional file 6 — Domains consisting of 4SSEs from SCOP version 1.69. This file lists the identifiers for the 4SSEs containing domains from SCOP version 1.69. [file 1471-2105-11-364-S6.PDF]

Additional File 6

Domains consisting of 4SSEs from SCOP version 1.69

Table 1: Domains consisting of 4SSEs from SCOP version 1.69

**Domain Identifiers**							
d1a04a1	d1a04b1	d1a0pa1	d1a15a_	d1a15b_	d1a1ia1	d1a1ja1	d1a1ja2
d1a2aa_	d1a2ac_	d1a2ag_	d1a2ia_	d1a32a_	d1a3pa_	d1a4pb_	d1a6ia1
d1a6ya_	d1a7ba_	d1a7bc_	d1a7da_	d1a7ea_	d1a8ca_	d1ab1a_	d1acaa_
d1acpa_	d1adta1	d1adta2	d1adua1	d1adua2	d1adub1	d1adva1	d1adva2
d1af7a1	d1afpa_	d1agta_	d1ahoa_	d1ak8a_	d1akhb_	d1an1i_	d1anva1
d1aoid_	d1aoie_	d1aoih_	d1aoja_	d1aojb_	d1ap0a_	d1ap5a1	d1ap6a1
d1apoa_	d1apqa_	d1ataa_	d1atba_	d1atda_	d1atea_	d1atxa_	d1au7a2
d1auea_	d1aueb_	d1autl1	d1autl2	d1auua_	d1auub_	d1avyc_	d1awwa_
d1ayia_	d1ayja_	d1azya1	d1azyb1	d1b1ba2	d1b22a_	d1b2ta_	d1b3aa_
d1b3ca_	d1b3ob3	d1b4ca_	d1b4cb_	d1b69a_	d1b72b_	d1b7da_	d1b7ta1
d1b8ta3	d1b9mb4	d1b9na4	d1b9nb4	d1b9wa1	d1b9wa2	d1bb8a_	d1bbga_
d1bc5a1	d1bccf_	d1bdha1	d1bdia1	d1be2a_	d1be3f_	d1beaa_	d1bf5a1
d1bfaa_	d1bg1a3	d1bg8a_	d1bg8b_	d1bg8c_	d1bgyf_	d1bgyr_	d1bh8b_
d1bhpa_	d1bi0a2	d1bi1a2	d1bi2a2	d1bi2b2	d1bi3a2	d1bi3b2	d1biba2
d1bj0a1	d1bjya1	d1bjyb1	d1bjza1	d1bk8a_	d1bl0a1	d1bl0a2	d1bl9a1
d1bmlc3	d1bmld3	d1bmra_	d1bmwa_	d1bnoa_	d1bnpa_	d1bo0a_	d1boda_
d1bpda2	d1bpxa3	d1bpya3	d1br4a1	d1br4c1	d1br4e1	d1br4g1	d1brci_
d1brwb1	d1brza_	d1bsha1	d1bt6a_	d1bv2a_	d1bvsa1	d1bvsb1	d1bvsc1
d1bvse1	d1bvsf1	d1bvsg1	d1bvsh1	d1bw6a_	d1bxia_	d1bxpa_	d1c0wa2
d1c0wb2	d1c17m_	d1c55a_	d1c5aa_	d1c75a_	d1c7ua_	d1c7ub_	d1c89a1
d1c8aa1	d1c8pa_	d1c9pb_	d1cbna_	d1ccda_	d1ccfa_	d1ccma_	d1ccna_
d1ceab_	d1ceia_	d1ceja1	d1cfaa_	d1cfma2	d1cfmb2	d1cfmc2	d1cgii_
d1chca_	d1choi_	d1ci3m2	d1cita_	d1cjga_	d1cjgb_	d1cm9a_	d1cm9b_
d1cmab_	d1cmfa_	d1cmga_	d1cn2a_	d1cnea1	d1cnpa_	d1cnra_	d1cnt1_
d1cnt3_	d1cnt4_	d1coai_	d1coka_	d1cowe1	d1cowg_	d1cpra_	d1cqea2
d1cqtb2	d1crcb_	d1crna_	d1csoi_	d1ct0i_	d1ct2i_	d1ct4i_	d1ctla1
d1ctxa_	d1cunc1	d1cvua2	d1cvub2	d1cx2a2	d1cx2b2	d1cx2c2	d1cx2d2
d1cxp.2	d1cyla_	d1d0xa1	d1d0za1	d1d1aa1	d1d1ba1	d1d1ca1	d1d1da1
d1d2v.2	d1d5l.1	d1d5l.2	d1d5ya1	d1d5ya2	d1d5yb2	d1d5yc2	d1d5yd2
d1d7w.2	d1danl1	d1danl2	d1dc8a_	d1deca_	d1dfka1	d1dfla1	d1dflb1
d1diid_	d1diqc_	d1diqd_	d1diya2	d1dj8a_	d1dj8b_	d1dj8c_	d1dj8d_
d1dj8f_	d1dk1a_	d1dk2a_	d1dk3a_	d1dkga2	d1dlia1	d1dlja1	d1dnu.1
d1dnw.1	d1dnw.2	d1dnya_	d1doka_	d1dokb_	d1dola_	d1doma_	d1domb_
d1donb_	d1doxa_	d1doya_	d1dq7a_	d1dq7b_	d1ds2i_	d1ds3i_	d1dsza_
d1dt0b1	d1dt0c1	d1du7a1	d1dval1	d1dval2	d1dvam1	d1dvam2	d1dwla_
d1dx5i1	d1dx5i3	d1dx5j1	d1dx5j3	d1dx5k1	d1dx5k3	d1dx5l1	d1dx5l3
d1e0ea_	d1e0eb_	d1e0ga_	d1e1rg_	d1e2va2	d1e2vb2	d1e2vc2	d1e2wa2
d1e2za2	d1e2zb2	d1e2zc2	d1e3oc2	d1e4ta_	d1e53a_	d1e5ga1	d1e6jp1
d1e7ja_	d1e8pa_	d1e8qa_	d1e91a_	d1eaic_	d1eaid_	d1eaka3	d1eaka4
d1eakc5	d1eakd3	d1eakd4	d1eakd5	d1eboa_	d1ebob_	d1ebod_	d1eboe_
d1ebva2	d1ed0a_	d1ed7a_	d1ee8a1	d1ee8b1	d1efaa1	d1efab1	d1efre1
d1eg3a3	d1eg4a3	d1egla_	d1egwa_	d1egwb_	d1egwc_	d1egwd_	d1eh2a_

Continued on Next Page...

Table 1 – Continued

Domain Identifiers							
d1ehda2	d1ehha1	d1ehha2	d1ehhb1	d1ehhb2	d1eiaa1	d1eiga_	d1eiha_
d1eisa2	d1ejab_	d1ejga_	d1ekya_	d1el0a_	d1em1a1	d1em1b1	d1emna1
d1en2a1	d1en2a2	d1en4c1	d1en5a1	d1en6d1	d1enia_	d1enja_	d1enma2
d1eoqa_	d1eota_	d1epja_	d1ept.1	d1eqga2	d1eqgb2	d1eqha2	d1eqhb2
d1eqtb_	d1eqzb_	d1eqzc_	d1eqzf_	d1eqzg_	d1esla2	d1esra_	d1ethb2
d1etka_	d1etkb_	d1etpa1	d1etpb1	d1etqc_	d1etva_	d1etvb_	d1etwa_
d1etxb_	d1etya_	d1etyb_	d1ewha2	d1ewhb2	d1ewhc2	d1exea_	d1exeb_
d1exta2	d1extb1	d1extb2	d1extb3	d1ezja_	d1ezzb2	d1f0rb_	d1f0sb_
d1f2ii1	d1f2ij1	d1f2la_	d1f2lb_	d1f2lc_	d1f2ld_	d1f4sp_	d1f5aa3
d1f66a_	d1f66d_	d1f66e_	d1f66h_	d1f6va_	d1f7ea_	d1f7ma_	d1f7ya_
d1f9pa_	d1f9qa_	d1f9qb_	d1f9qc_	d1f9qd_	d1f9ra_	d1f9rb_	d1f9rc_
d1f9sb_	d1f9sc_	d1f9sd_	d1fakl1	d1faqa_	d1fara_	d1faxl_	d1fbra2
d1fcdc1	d1fcdd1	d1fcdd2	d1fcla_	d1fd3a_	d1fd3b_	d1fd3c_	d1fd4b_
d1fd4d_	d1fd4f_	d1fd4h_	d1fd4j_	d1fd4k_	d1fd4l_	d1fd4o_	d1fd4p_
d1ff1a_	d1ffha1	d1fftb2	d1fftg2	d1ffua1	d1ffud1	d1ffva1	d1ffvd1
d1fi8.2	d1fi9a_	d1fiaa_	d1fiab_	d1fipa_	d1fipb_	d1fiqa1	d1fjgo_
d1fjsl_	d1fkao_	d1fl7d_	d1fmva1	d1fmwa1	d1fo4a1	d1fo4b1	d1fpoa1
d1fpoc1	d1fr2a_	d1fs1b1	d1fs1d1	d1fs2b1	d1fs2d1	d1fsea_	d1fseb_
d1fsed_	d1fsee_	d1ft4a1	d1ft4a2	d1ft4b1	d1ft4b2	d1ft4b3	d1ft8e_
d1fwza2	d1fwza3	d1fx7b2	d1fx7c2	d1fx7d2	d1fxka_	d1fxkb_	d1fyca_
d1g1qa2	d1g1qb2	d1g1qc2	d1g1qd2	d1g1ra2	d1g1rb2	d1g1rc2	d1g1rd2
d1g1sb2	d1g1ta2	d1g1xb_	d1g1xg_	d1g26a_	d1g2914	d1g2924	d1g2lb_
d1g2sa_	d1g2ta_	d1g3ta2	d1g3tb2	d1g3ya2	d1g44a1	d1g44b1	d1g44c1
d1g5va_	d1g6pa_	d1g6rc2	d1g6za_	d1g91a_	d1ga5a_	d1ga5b_	d1ga5e_
d1gb4a_	d1gbra_	d1gcca_	d1gdca_	d1gg3a1	d1gg3b1	d1gg3c1	d1gh1a_
d1gkga2	d1glua_	d1go5a_	d1gpsa_	d1gpta_	d1gqmb_	d1gqmd_	d1gqme_
d1gqmi_	d1gqmk_	d1gqml_	d1gt0c2	d1gv3a1	d1gv3b1	d1gxba1	d1gxbb1
d1gxbd1	d1gxdb1	d1gxga_	d1gxha_	d1gyja_	d1gyjb_	d1gyxa_	d1gyxb_
d1gyyb_	d1gyza_	d1h0ma1	d1h0mb1	d1h0mc1	d1h0md1	d1h1ob1	d1h31b_
d1h31f_	d1h31h_	d1h3la_	d1h3lb_	d1h59b_	d1h6gb1	d1h6w.2	d1h7ca_
d1h8pa1	d1h8pb1	d1h9ra2	d1h9rb2	d1h9sa2	d1h9sb2	d1ha6a_	d1hb6a_
d1hb8b_	d1hb8c_	d1hbka_	d1hbxa_	d1hcgb_	d1hcia2	d1hcib2	d1hcnb_
d1hcqa_	d1hcqb_	d1hcqe_	d1hcqf_	d1hcza2	d1hdja_	d1hf0a2	d1hf0b2
d1hfet_	d1hfga_	d1hfna_	d1hhva_	d1hiai_	d1hiaj_	d1hjai_	d1hjpa2
d1hlva1	d1hlza_	d1hlzb_	d1hmda_	d1hmdb_	d1hmdc_	d1hmdd_	d1hmoa_
d1hmoc_	d1hmod_	d1hnwo_	d1hnws_	d1hnxo_	d1hnxs_	d1hnzo_	d1hnzs_
d1hp2a_	d1hpia_	d1hpja_	d1hpta_	d1hq3b_	d1hq3c_	d1hq3f_	d1hq3g_
d1hr0o_	d1hr0s_	d1hrja_	d1hrjb_	d1hrra_	d1hrti_	d1hsna_	d1ht5a2
d1ht8a2	d1ht8b2	d1huma_	d1humb_	d1huna_	d1hunb_	d1huoa3	d1huob3
d1hx1b_	d1hx2a_	d1hx8a1	d1hx8b1	d1hy8a_	d1hywa_	d1hz8a1	d1hz8a2
d1i07a_	d1i07b_	d1i08a1	d1i0ha1	d1i0ua1	d1i0ua2	d1i2ta_	d1i2ua_
d1i3qf_	d1i3qj_	d1i49a_	d1i4da_	d1i4la_	d1i4ta_	d1i4ya_	d1i4yb_
d1i4yd_	d1i4ye_	d1i4yf_	d1i4yg_	d1i4yh_	d1i4za_	d1i4zb_	d1i4zc_
d1i4ze_	d1i4zf_	d1i4zg_	d1i4zh_	d1i50f_	d1i50i2	d1i50j_	d1i6hf_
d1i6ve_	d1i71a_	d1i72.1	d1i79.1	d1i7b.1	d1i7c.1	d1i7m.1	d1i7m.2
d1i94m_	d1i94n_	d1i94o_	d1i94s_	d1ibia1	d1ibko_	d1ibks_	d1iblo_
d1ibmo_	d1ibxa_	d1ibxb_	d1icwa_	d1icwb_	d1id3a_	d1id3c_	d1id3d_

Continued on Next Page…

Table 1 – Continued

Domain Identifiers							
d1id3h_	d1ifwa_	d1igza2	d1ijqa2	d1ijqb2	d1ijua_	d1ijub_	d1ijuc_
d1ijva_	d1ijvb_	d1ikla_	d1ikma_	d1il8a_	d1il8b_	d1ilpa_	d1ilpb_
d1ilqb_	d1impa_	d1imqa_	d1in0a1	d1in0a2	d1in0b1	d1in0b2	d1in1a_
d1iqbb2	d1iqfl_	d1iqhl_	d1iqjl_	d1iqkl_	d1iqnl_	d1irjb_	d1irjd_
d1irjf_	d1irjg_	d1irla_	d1isaa1	d1isab1	d1isba1	d1isbb1	d1isca1
d1iv5b_	d1ivoc_	d1ivod_	d1iw4a_	d1iw7e_	d1iw7o_	d1ixca1	d1iy5a_
d1iyra_	d1j0ta_	d1j1ef_	d1j2la_	d1j2ma_	d1j2na_	d1j5eo_	d1j5es_
d1j78b3	d1j7oa_	d1j7qa_	d1j7ra_	d1j8ia_	d1j8mf1	d1j8yf1	d1ja8a1
d1jada_	d1jadb_	d1je4a_	d1je8a_	d1je8b_	d1je8e_	d1je8f_	d1jen.3
d1jfsa1	d1jfta1	d1jh9a1	d1jhna3	d1ji7a_	d1ji7b_	d1ji7c_	d1jjua1
d1jkfa2	d1jkfb2	d1jkza_	d1jl9a_	d1jl9b_	d1jm4b_	d1jmab1	d1jmab2
d1jmsa1	d1jn3a1	d1jnia_	d1jpja1	d1jpna1	d1jpnb1	d1jrab_	d1jspb_
d1jt0a1	d1jt0b1	d1jt0c1	d1jt0d1	d1jt6a1	d1jt6d1	d1jt6e1	d1jtxa1
d1jtxe1	d1jtya1	d1jtyd1	d1jtye1	d1juma1	d1jumb1	d1jumd1	d1jume1
d1jupb1	d1jupd1	d1jupe1	d1jusa1	d1jusb1	d1jusd1	d1juse1	d1jvra_
d1jvsb1	d1jw2a_	d1jwla1	d1jwlb1	d1jwya1	d1jx2a1	d1jxta_	d1jxua_
d1jxxa_	d1jxya_	d1jy4a_	d1jy4b_	d1jzaa_	d1jzab_	d1jzba_	d1k1wa1
d1k1xb1	d1k1ya1	d1k1yb1	d1k25a1	d1k25a2	d1k25b2	d1k3wa1	d1k3xa1
d1k50b_	d1k50d_	d1k51a_	d1k5ha1	d1k5hb1	d1k5hc1	d1k6ob_	d1k7ua1
d1k82b1	d1k82c1	d1k82d1	d1k83f_	d1k83i2	d1k83j_	d1k9ba_	d1k9kb_
d1kd12_	d1kdha1	d1keja1	d1keja3	d1kfta_	d1kfva1	d1kfvb1	d1kgza1
d1khda1	d1khdb1	d1khdc1	d1khdd1	d1kigl_	d1kj6a_	d1kjka_	d1kk7a1
d1kkcb1	d1kkcx1	d1kloa2	d1klpa_	d1knaa_	d1knea_	d1kohc2	d1kooc2
d1kqsz_	d1krna_	d1ksnb_	d1ktkf1	d1ku7d_	d1kuqa_	d1kvd.2	d1kve.2
d1kx3a_	d1kx3d_	d1kx3e_	d1kx3h_	d1kx4a_	d1kx4d_	d1kx4e_	d1kx4h_
d1kx5d_	d1kx5e_	d1kx5h_	d1kyeb_	d1l0ha_	d1l0ia_	d1l0lf_	d1l0lh_
d1l0nf_	d1l1ca_	d1l1cb_	d1l1ma_	d1l1mb_	d1l1ta1	d1l1za1	d1l2ba1
d1l2da1	d1l2oa1	d1l2wi_	d1l2wj_	d1l2wk_	d1l2za_	d1l3eb_	d1l3la1
d1l3lc1	d1l3ld1	d1l4ad_	d1l6ka_	d1l6kb_	d1l6ke_	d1l6kf_	d1l6kj_
d1l6l2_	d1l6l3_	d1l6l7_	d1l6l8_	d1l6le_	d1l6lg_	d1l6lj_	d1l6ll_
d1l6ln_	d1l6lp_	d1l6lt_	d1l6lu_	d1l7cb1	d1l8ca_	d1l8da_	d1l8db_
d1l9la_	d1laca_	d1lata_	d1latb_	d1lb2b_	d1lb2e_	d1lcka1	d1ldkd1
d1le8b_	d1lfup_	d1lgla_	d1lkya_	d1lkyb_	d1lkyc_	d1lkyd_	d1lkye_
d1ll1a1	d1llaa1	d1lm3b_	d1lm3d_	d1lnqb2	d1lnqc2	d1lnqd2	d1lpba2
d1lpka_	d1lpza_	d1lqda_	d1lqha_	d1lqia_	d1lqqa_	d1lr7a2	d1lr8a2
d1ls1a1	d1luva1	d1luwa1	d1luwb1	d1lv9a_	d1lvka1	d1lw6i_	d1lwub2
d1lxea_	d1m12a_	d1m18a_	d1m18d_	d1m18e_	d1m18h_	d1m19a_	d1m19d_
d1m19h_	d1m1aa_	d1m1ac_	d1m1ad_	d1m1ae_	d1m1ah_	d1m1jb2	d1m2oa5
d1m6zb1	d1m6zc1	d1m6zd1	d1m70a1	d1m70b1	d1m70c1	d1m70d1	d1m71b2
d1m8aa_	d1m8ab_	d1m8ba_	d1m8ca_	d1m93.1	d1m9sa2	d1mabg_	d1mfza1
d1mfzc1	d1mfzd1	d1mgsa_	d1mgsb_	d1mhl.1	d1mhl.2	d1mhm.1	d1mhna_
d1mi2a_	d1mi2b_	d1mi7r_	d1mita_	d1mizb1	d1mj2b_	d1mj2c_	d1mj2d_
d1mjoa_	d1mjob_	d1mjoc_	d1mjod_	d1mjqa_	d1mjqb_	d1mjqc_	d1mjqg_
d1mjqi_	d1mjqj_	d1mk7b1	d1mk7d1	d1mk9b1	d1mk9f1	d1mk9h1	d1ml0d_
d1mmga1	d1mmna1	d1mnda1	d1mnea1	d1mnga1	d1mngb1	d1mnta_	d1moga_
d1mq1a_	d1mq1b_	d1mq3a2	d1mq5l_	d1mq6l_	d1mqva_	d1mr4a_	d1msga_
d1mshb_	d1mu5a1	d1muua1	d1muub1	d1muuc1	d1muud1	d1mv8a1	d1mv8b1

Continued on Next Page…

**Domain Identifiers**							
d1mv8d1	d1mx0a1	d1mx0b1	d1mx0c1	d1mx0d1	d1mx0e1	d1mx0f1	d1my6a1
d1myna_	d1myp.1	d1myp.2	d1mz8a_	d1mz8c_	d1n0jb1	d1n0na1	d1n0nb1
d1n1ia2	d1n1ib1	d1n1ib2	d1n1ic1	d1n1ic2	d1n1id1	d1n1id2	d1n1ja_
d1n33o_	d1n34i_	d1n34o_	d1n36c2	d1n36i_	d1n36j_	d1n36o_	d1n4ia_
d1n4ya_	d1n5wa1	d1n5wd1	d1n5xa1	d1n5xb1	d1n60a1	d1n60d1	d1n61a1
d1n62a1	d1n62d1	d1n63a1	d1n63d1	d1n69a_	d1n69b_	d1n69c_	d1n87a_
d1n9ja_	d1n9jb_	d1napa_	d1napb_	d1napc_	d1napd_	d1nbba_	d1ncfa1
d1ncfb1	d1ncfb2	d1ncfb3	d1ncsa_	d1ncva_	d1ncvb_	d1ne3a_	d1ne5a_
d1nekd_	d1nenc_	d1nend_	d1newa_	d1nf4b_	d1nf4c_	d1nf4h_	d1nf4i_
d1nf6d_	d1nf6e_	d1nf6g_	d1nf6i_	d1nf6k_	d1nf6p_	d1nfub_	d1nfvb_
d1nfve_	d1nfvg_	d1nfvh_	d1nfvj_	d1nfvk_	d1nfvp_	d1nfwb_	d1nfxb_
d1ng1a1	d1ngmf_	d1nikf_	d1nikj_	d1nji2_	d1nkla_	d1nl1a2	d1nl2a2
d1nlxa_	d1nlxb_	d1nlxc_	d1nlxd_	d1nlxe_	d1nlxf_	d1nlxg_	d1nlxh_
d1nlxj_	d1nlxk_	d1nlxl_	d1nlxm_	d1nlxn_	d1nmia_	d1nnja1	d1nola1
d1npia_	d1nppd2	d1nr2a_	d1nr2b_	d1nr4a_	d1nr4b_	d1nr4c_	d1nr4d_
d1nr4f_	d1nr4g_	d1nr4h_	d1nraa_	d1nrba_	d1nrka1	d1nrn.1	d1nrp.1
d1nsgb_	d1nt0a3	d1nt0g3	d1ntca_	d1ntcb_	d1ntia_	d1ntkf_	d1ntkh_
d1ntzf_	d1nu1c1	d1nuib2	d1nvla_	d1nwmx_	d1nwva1	d1ny2.1	d1ny4a_
d1nyra2	d1nz6b_	d1nzia2	d1nzib2	d1nzpa_	d1o17a1	d1o17b1	d1o17c1
d1o4xa2	d1o5dl1	d1o5dl2	d1o6wa2	d1o7la3	d1o7lb3	d1o7lc3	d1o7ld3
d1o7yc_	d1o7yd_	d1o7za_	d1o80a_	d1o80b_	d1o87a1	d1o87b1	d1ob1c1
d1ob1f1	d1ob1f2	d1ocda_	d1octc2	d1odba_	d1odbb_	d1odbc_	d1odbd_
d1oe9a1	d1oeda_	d1oedb_	d1oedc_	d1oedd_	d1ofcx3	d1oipa1	d1ok9b4
d1okhb_	d1okka1	d1omta_	d1omua_	d1on1a2	d1on1b2	d1on2a2	d1on2b2
d1onnb1	d1onoa1	d1onob1	d1onpa1	d1onpb1	d1oqdm_	d1oqdo_	d1oqdp_
d1oqdr_	d1oqya3	d1or7a1	d1or7b1	d1or7b2	d1or7c_	d1or7f_	d1orja_
d1orjc_	d1orjd_	d1orka1	d1orla_	d1orsc_	d1orya_	d1otpa1	d1ovoa_
d1ovoc_	d1ovod_	d1owaa_	d1oxja1	d1oxya1	d1oz4a1	d1oz4b1	d1oz4c1
d1ozza_	d1p00a_	d1p0aa_	d1p0sl1	d1p34a_	d1p34d_	d1p34e_	d1p34h_
d1p3ad_	d1p3ae_	d1p3ah_	d1p3ba_	d1p3bd_	d1p3be_	d1p3bh_	d1p3fa_
d1p3fe_	d1p3fh_	d1p3ga_	d1p3gd_	d1p3ge_	d1p3gh_	d1p3ia_	d1p3id_
d1p3ih_	d1p3ka_	d1p3kd_	d1p3ke_	d1p3kh_	d1p3la_	d1p3ld_	d1p3le_
d1p3ma_	d1p3md_	d1p3me_	d1p3mh_	d1p3oa_	d1p3od_	d1p3oe_	d1p3oh_
d1p3pd_	d1p3pe_	d1p3ph_	d1p4qb_	d1p65a_	d1p65b_	d1p7ba2	d1p7bb2
d1p92a2	d1p9ga_	d1p9ja_	d1pb6c1	d1pbya1	d1pcea_	d1pd0a5	d1pdqa_
d1pfba_	d1pfma_	d1pfmb_	d1pfmc_	d1pfmd_	d1pfna_	d1pfnb_	d1pfnc_
d1pfxl1	d1pgea2	d1pgeb2	d1pgfa2	d1pgfb2	d1pgga2	d1pggb2	d1piha_
d1pjia1	d1pjja1	d1pjua2	d1pjub2	d1pjuc2	d1pjud2	d1pk4a_	d1pl4a1
d1plfa_	d1plfb_	d1plfc_	d1plfd_	d1pm5a1	d1pm9a1	d1pm9b1	d1pmra_
d1pnso_	d1pnss_	d1pnxo_	d1pnxs_	d1poua_	d1pp9f_	d1pp9s_	d1ppfi_
d1ppjs_	d1ppqa_	d1prch2	d1prha2	d1prhb2	d1pspa1	d1pspb1	d1ptha2
d1ptqa_	d1ptra_	d1ptxa_	d1pu1a_	d1pufb_	d1pvea_	d1pvza_	d1px9a_
d1pxxb2	d1pxxc2	d1pxxd2	d1pyya1	d1pyya2	d1pzna1	d1pzwa_	d1q08a_
d1q09a_	d1q0aa_	d1q0ab_	d1q0ha1	d1q0la1	d1q0qa1	d1q0qb1	d1q38a_
d1q3ba1	d1q3ca1	d1q3la_	d1q4ga2	d1q4gb2	d1q78a1	d1q7ia_	d1q822_
d1q95g2	d1q95i2	d1q95j2	d1q95l1	d1qbha_	d1qe6a_	d1qe6b_	d1qe6c_
d1qfkl1	d1qfwb_	d1qg7a_	d1qgba1	d1qjda1	d1qk9a_	d1qkfa_	d1qlia1

Continued on Next Page. . .

Table 1 – Continued

Domain Identifiers							
d1qlkb_	d1qlya_	d1qmea1	d1qmea2	d1qmfa1	d1qmfa2	d1qnmb1	d1qnna1
d1qnnc1	d1qnnd1	d1qo6a1	d1qp0a1	d1qp4a1	d1qp7a1	d1qpia1	d1qpua_
d1qq3a_	d1qqaa1	d1qqba1	d1qqva_	d1qrjb1	d1qu1c_	d1quua1	d1quua2
d1qvia1	d1qvta1	d1qvtb1	d1qvte1	d1qvua1	d1qvud1	d1qvue1	d1qypa_
d1qzwa1	d1qzwc1	d1qzwe1	d1qzwg1	d1r0bg2	d1r0bl2	d1r0ka1	d1r0kb1
d1r0kd1	d1r0la1	d1r0lb1	d1r0lc1	d1r0ld1	d1r0na_	d1r0nb_	d1r0ri_
d1r2za1	d1r4ae_	d1r4af_	d1r4ia_	d1r4oa_	d1r4ob_	d1r4ra_	d1r4rb_
d1r5la1	d1r5oa2	d1r5qa_	d1r5uf_	d1r5uj_	d1r6ra_	d1r6rb_	d1r79a_
d1r8jb1	d1r8ub_	d1r9sf_	d1r9sj_	d1r9tf_	d1r9tj_	d1rcpa_	d1rfnb_
d1rfyb_	d1rg6a_	d1rgbl1	d1rgva_	d1rhpa_	d1rhpb_	d1rhpd_	d1ri9a_
d1rida4	d1ridb3	d1ridb4	d1rioh_	d1rj9a1	d1rjpa2	d1rjqa2	d1rjra2
d1rk6a2	d1rkca2	d1rkea1	d1rkea2	d1rkta1	d1rktb1	d1rkwa1	d1rkwd1
d1rl2a2	d1rl2b2	d1rm6c1	d1rm6f1	d1rnla1	d1roda_	d1rodb_	d1rp3a2
d1rp3c2	d1rp3d_	d1rp3e2	d1rp3f_	d1rp3g2	d1rp3g3	d1rp3h_	d1rp5a1
d1rp5b1	d1rp5b2	d1rpla1	d1rpwb1	d1rpwc1	d1rpwd1	d1rtna_	d1rtnb_
d1rtob_	d1rutx2	d1rutx3	d1rwtb_	d1rwtc_	d1rwte_	d1rwth_	d1rwti_
d1ryka_	d1ryua_	d1rzra1	d1rzrc1	d1rzrd1	d1rzrg1	d1rzsa_	d1rzta1
d1rzti1	d1rztm1	d1s1hs_	d1s1ih_	d1s1in_	d1s1io_	d1s1it_	d1s1ix_
d1s32d_	d1s32e_	d1s32h_	d1s4za_	d1s5ga1	d1s5qb_	d1s5rb_	d1s78a4
d1s7ba_	d1s7bb_	d1s7bc_	d1s7bd_	d1s7be_	d1s7bf_	d1s7bg_	d1s7bh_
d1sb3c1	d1sb3f1	d1sbja_	d1sc5a2	d1sc5a3	d1sc5b_	d1sc7a1	d1sced_
d1scva_	d1sdfa_	d1sera1	d1serb1	d1sesa1	d1sesb1	d1seta1	d1setb1
d1sfkc_	d1sfkd_	d1sfkf_	d1sfkg_	d1sfof_	d1sfoj_	d1sfwa_	d1sgdi_
d1sgga_	d1sgma1	d1sgmb1	d1sgni_	d1sgpi_	d1sgqi_	d1sgri_	d1sgyi_
d1sibi_	d1sisa_	d1sj8a2	d1sknp_	d1skub2	d1skza2	d1smye_	d1smyo_
d1sozc1	d1sqbf_	d1sqqf1	d1sqxf1	d1sr6a1	d1srla_	d1srya1	d1sryb1
d1st6a4	d1suya_	d1suyb_	d1sv0a_	d1sv0b_	d1sv1a_	d1sv1b_	d1sv4a_
d1sxea_	d1syqa1	d1syqa2	d1szba2	d1szbb2	d1szpa1	d1szpb1	d1szpc1
d1szpe1	d1szpf1	d1szxa1	d1szxb1	d1t01a1	d1t01a2	d1t1ra1	d1t1rb1
d1t1sb1	d1t33b1	d1t38a2	d1t3ua_	d1t3ub_	d1t3uc_	d1t3ud_	d1t4ga1
d1t7aa_	d1t8ka_	d1t92a_	d1t92b_	d1tbqr1	d1tbqr2	d1tbqs1	d1tbqs2
d1tbrr2	d1tbrs1	d1tbrs2	d1tcpa_	d1tdza1	d1teci_	d1tfba2	d1tfia_
d1tfob1	d1tgsi_	d1tina_	d1tk7a2	d1tkna_	d1tlea_	d1tlva2	d1tm1i_
d1tm4i_	d1tm5i_	d1tmgi_	d1tmu.1	d1tn9a_	d1tnrr1	d1tnrr2	d1tnrr3
d1to2i_	d1tpka_	d1tqga_	d1tska_	d1tu0d2	d1tura_	d1tusa_	d1tvaa2
d1tvxa_	d1tvxb_	d1tvxc_	d1tvxd_	d1twaf_	d1twai2	d1twaj_	d1twcf_
d1twcj_	d1twff_	d1twfj_	d1twgf_	d1twgi2	d1twgj_	d1twhf_	d1twhi2
d1tzyb_	d1tzyc_	d1tzyf_	d1tzyg_	d1u00a1	d1u35a1	d1u35d1	d1u35e1
d1u4la_	d1u4lb_	d1u4ma_	d1u4mb_	d1u4pa_	d1u4pb_	d1u4qa1	d1u4qb3
d1u4rb_	d1u4rc_	d1u4rd_	d1u5sb1	d1u5td1	d1u84a_	d1u8cb4	d1u8ra2
d1u8rg2	d1u9na1	d1u9oa1	d1uera1	d1uerb1	d1uerc1	d1uerd1	d1uesa1
d1uesc1	d1uesd1	d1ugoa_	d1ui5a1	d1ui5b1	d1ui6b1	d1ujza_	d1ul1z1
d1un6b1	d1unfx1	d1unka_	d1unkb_	d1unkc_	d1unkd_	d1uoua1	d1upga_
d1urka2	d1urua_	d1us6a_	d1utga_	d1utra_	d1utrb_	d1uuca_	d1uujb_
d1uujd_	d1uwoa_	d1uwob_	d1uzja1	d1uzjb1	d1uzjc1	d1uzka1	d1uzpa1
d1v3xb_	d1v43a1	d1v43a2	d1v4ya2	d1v51a2	d1v5ka_	d1v63a_	d1v64a_
d1v74b_	d1v7ba1	d1v7bb1	d1v8ga1	d1v8gb1	d1v97a1	d1v97b1	d1v9va1

Continued on Next Page…

Table 1 – Continued

Domain Identifiers							
d1varb1	d1vcia1	d1vcia2	d1vcsa1	d1vdja1	d1vdla_	d1vdva1	d1vdvb1
d1vfia1	d1vh6a_	d1vhkb1	d1vi0a1	d1vi0b1	d1vkua_	d1vlia1	d1vmca1
d1vmpa_	d1vnaa_	d1vnba_	d1voqq_	d1vorg_	d1vosm_	d1vosq_	d1voug_
d1vovq_	d1vowg_	d1voxq_	d1voyg_	d1vozm_	d1vozq_	d1vp0g_	d1vpua_
d1vpza_	d1vpzb_	d1vq411	d1vq511	d1vq711	d1vq811	d1vq911	d1vqk11
d1vqm11	d1vqn11	d1vqo11	d1vqp11	d1vr9a1	d1vr9b1	d1vsau1	d1vvca2
d1w2ea_	d1w2eb_	d1w2kl2	d1w53a_	d1w5ce_	d1w5ck_	d1w7ia1	d1w7ja1
d1wb3d1	d1wcmf_	d1wcmj_	d1weea_	d1wepa_	d1weta1	d1wewa_	d1wfea_
d1wfya_	d1wgfa_	d1wgna_	d1wgsa_	d1wgwa_	d1wiea_	d1wiia_	d1wj2a_
d1wjca_	d1wjcb_	d1wjda_	d1wjdb_	d1wjea_	d1wjeb_	d1wjza_	d1wlpb1
d1wrts_	d1wssl2	d1wtgl2	d1wu1b1	d1wudb1	d1wunl2	d1wuwa_	d1wuwb_
d1wvec1	d1wved1	d1wz3a1	d1wz3b1	d1x3wb1	d1x3zb1	d1x40a1	d1x57a1
d1x79a_	d1x7al1	d1x86g2	d1x9xa1	d1x9xb1	d1xb4c2	d1xc8a1	d1xcbf1
d1xdoa1	d1xdob1	d1xdpa1	d1xdpb1	d1xdtr_	d1xfea1	d1xfxo1	d1xfxp1
d1xfxr1	d1xfxs1	d1xfxt1	d1xi7a_	d1xila1	d1xj1a_	d1xk4c1	d1xkal1
d1xkba1	d1xkba2	d1xkbb1	d1xkbb2	d1xl3c1	d1xl3d1	d1xl4a2	d1xl4b2
d1xl6b2	d1xmoo_	d1xmos_	d1xmqo_	d1xnqo_	d1xnro_	d1xp4a1	d1xp4b1
d1xrsb2	d1xsli1	d1xslm1	d1xsna1	d1xsna2	d1xspa1	d1xspa2	d1xu1s_
d1xu6a_	d1xwra1	d1xyda1	d1xydb1	d1xyka_	d1y15a_	d1y19b1	d1y19d1
d1y19h1	d1y19j1	d1y19l1	d1y1vf_	d1y1vj_	d1y1vs_	d1y1wf_	d1y1wj_
d1y67b1	d1y67c1	d1y67d1	d1y77f_	d1y77j_	d1y7ma2	d1y7ya1	d1y7yb1
d1y8ea3	d1y8ea4	d1y8eb1	d1y8eb4	d1y9bb1	d1yfca_	d1yfhc2	d1ygcl1
d1yij11	d1yit11	d1yj911	d1yjw11	d1ykeb1	d1yl3w1	d1yl4r1	d1ymma2
d1ynrb1	d1ynrc1	d1ynrd1	d1ypai_	d1ypci_	d1yrnb_	d1yt3a1	d1yt3a2
d1yu6c1	d1yu6d1	d1yuaa1	d1yuaa2	d1yuia_	d1yura1	d1yurb1	d1yusa1
d1yuta1	d1yutb1	d1yuub1	d1z0xa1	d1z0xb1	d1z1ba1	d1z1bb1	d1z1ga1
d1z1gc1	d1z1gd1	d1z1va1	d1z59a1	d1z5aa1	d1z5ab1	d1z5ba1	d1z5bb1
d1z5cb1	d1z6eb1	d1z6jl2	d1z77a1	d1z92b1	d1za5a1	d1za5b1	d1zaya1
d1zfla1	d1zg1a1	d1zg1b1	d1zg1e1	d1zg1f1	d1zg5a1	d1zg5b1	d1zg5e1
d1zgha1	d1zgla2	d1zgld2	d1zglg2	d1zglj2	d1zhva1	d1zk8a1	d1zkga1
d1zkra2	d1zlaa1	d1zlad1	d1zlae1	d1zlah1	d1zm2c4	d1zm3c4	d1znva1
d1zpqb1	d1zpqc1	d1zq1c1	d1zq1d1	d1zqha3	d1zqua1	d1zqva1	d1zqwa1
d1zqya1	d1zqza1	d1zr9a1	d1zs4b1	d1zs4d1	d1zspb1	d1ztea1	d1ztra1
d1zuqb1	d1zvva1	d1zvvb1	d1zvvg1	d1zxia1	d1zxid1	d1zxya1	d1zxyb1
d1zxyd1	d1zyka1	d1zykb1	d1zykc1	d1zykd1	d1zyre1	d1zyrf2	d1zyro1
d1zz6a1	d1zz6b1	d1zz7a1	d1zz7b1	d1zz8c1	d1zz9c1	d1zzba1	d1zzbb1
d1zzcb1	d2a06f1	d2a06s1	d2a2ql2	d2a68e1	d2a68f2	d2a68o1	d2a68p2
d2a69f2	d2a69o1	d2a69p2	d2a6ca1	d2a6cb1	d2a6ee1	d2a6ef2	d2a6eo1
d2a6he1	d2a6hf2	d2a6ho1	d2a6hp2	d2a7ta1	d2a7tb1	d2abda_	d2aczc1
d2adpa1	d2adqb1	d2aeil2	d2aerl2	d2af8a_	d2ahmb1	d2ai5a1	d2alca_
d2aqfa1	d2arob1	d2aroc1	d2arof1	d2arog1	d2auwa1	d2auwb1	d2avpa1
d2aw2b2	d2aw2y2	d2aw9a1	d2aw9b1	d2awbz1	d2axds1	d2ayla2	d2aylb2
d2az0b1	d2az2a1	d2b0ha1	d2b21a1	d2b3ca_	d2b5dx1	d2b5id1	d2b63f1
d2b64s1	d2b66h1	d2b66y1	d2b76n2	d2b8kf1	d2b8kj1	d2b8ol2	d2b9ms1
d2b9ny1	d2b9os1	d2b9ph1	d2b9py1	d2baya1	d2bayb1	d2bayc1	d2bayd1
d2bayf1	d2bb8a_	d2bbga_	d2bccf_	d2bcqa1	d2bcqa2	d2bcra1	d2bcra2
d2bcsa2	d2bcua1	d2bcua2	d2bcva1	d2bcva2	d2bdna1	d2bdoa_	d2bdsa_

Continued on Next Page...

Domain Identifiers

d2be5f2	d2be5o1	d2be5p2	d2bful2	d2bgxa1	d2bh7a1	d2bjca1	d2bjcb1
d2bkbb1	d2bkbc1	d2bkbd1	d2bm1a4	d2bmga1	d2bmta_	d2bnma1	d2bnmb1
d2bnoa1	d2bnob1	d2boha1	d2bokl1	d2bpga3	d2bpgb3	d2bpna1	d2bq6a1
d2bqwa1	d2brza_	d2buoa1	d2busa_	d2bw3a1	d2bw3b1	d2bykb1	d2bykd1
d2bymd1	d2bz6l1	d2c03a1	d2c03b1	d2c04a1	d2c04b1	d2c2vu1	d2c4fl2
d2cc7a1	d2cc8a1	d2cc9a1	d2ccba1	d2ccca1	d2ccyb_	d2ch7b1	d2ci2i_
d2cifa1	d2cjca1	d2cjib1	d2ck3e1	d2cnwa1	d2cnwb1	d2cnwc1	d2cqna1
d2cs3a1	d2cs7a1	d2cs7b1	d2cs7c1	d2ct5a1	d2ctda1	d2cuja1	d2cv5a1
d2cv5e1	d2cv5h1	d2cw0e1	d2cw0p2	d2d0nc1	d2d1jb1	d2d3eb1	d2d3ed1
d2d6fd1	d2d6ya1	d2d6yb1	d2d8ca1	d2dana1	d2diga1	d2dj8a1	d2dnaa1
d2dnva1	d2drpa1	d2drpd1	d2dvha_	d2e2ac_	d2e2hf1	d2e2hj1	d2e2if1
d2e2jf1	d2e2jj1	d2e5le2	d2e5lo1	d2e5ls1	d2e7sb1	d2e7si1	d2e7so1
d2egha1	d2eghb1	d2eiab1	d2ejna2	d2enda_	d2eota_	d2etna1	d2eula1
d2eulc1	d2euld1	d2ev0a2	d2ev0b2	d2ev5a2	d2ev5b2	d2ev6a2	d2ev6b2
d2ey4f1	d2eyaa1	d2eyba1	d2eyca1	d2eyda1	d2eywa1	d2eyya1	d2ezha_
d2f05a1	d2f1ia1	d2f1ja1	d2f23a1	d2f23b1	d2f3na1	d2f3nb1	d2f3nc1
d2f44a1	d2f44b1	d2f44c1	d2f4mb1	d2f4ob1	d2f4vc1	d2f4vo1	d2f5ca2
d2f5db2	d2f5ea2	d2f5eb2	d2f5fa2	d2f5fb2	d2f5qa1	d2f5sa1	d2f66b1
d2f6mb1	d2f76x1	d2f8na1	d2f8nd1	d2f8ne1	d2f8nh1	d2faca1	d2facb1
d2fadb1	d2faea1	d2faeb1	d2fapb_	d2fbqa1	d2fcca1	d2fccb1	d2ffha1
d2ffhc1	d2fj1a1	d2fj7a1	d2fj7d1	d2fj7e1	d2fj7h1	d2fmlb1	d2fmqa2
d2fq3a1	d2fq4a1	d2frca_	d2fzfb1	d2g00l1	d2g0ua1	d2g3ba1	d2g3bb1
d2g3kb1	d2g3kc1	d2g3kd1	d2g3ke1	d2g3kf1	d2g3kg1	d2g7ga1	d2g7la1
d2g98b2	d2g9ha2	d2gcca_	d2gd4a1	d2gd4l1	d2gdaa_	d2gdsa1	d2gdsb1
d2gdwa1	d2gdya1	d2gena1	d2gf4a1	d2gf7a1	d2gf7b1	d2gf7c1	d2gf7d1
d2gfaa2	d2gfab1	d2gfab2	d2gfna1	d2gfnb1	d2gkri1	d2gtaa1	d2gtab1
d2gtad1	d2gtga1	d2gtvx1	d2gvqa1	d2gvqb1	d2gvqc1	d2gvqd1	d2gwsa1
d2gwse2	d2gwsi1	d2gwsm1	d2gya31	d2gya32	d2h27a1	d2h27d1	d2h3eb2
d2h61b1	d2h61c1	d2h61d1	d2h61e1	d2h61f1	d2h61g1	d2h61h1	d2h9el1
d2hcca_	d2hcia1	d2hcib1	d2hevr1	d2hevr3	d2heyr1	d2heyr3	d2heyt1
d2hfia1	d2hgir1	d2hgpr1	d2hgrr1	d2hhhe2	d2hhho1	d2hhhs1	d2hiob_
d2hkja1	d2hmqa_	d2hmqb_	d2hmqc_	d2hmqd_	d2hmza_	d2hmzb_	d2hmzc_
d2hpcg1	d2hsga1	d2hspa_	d2htnb1	d2huja1	d2hyfa2	d2hyfb2	d2hyfc2
d2hygd2	d2i10a2	d2i1qa1	d2i2ty1	d2i2vy1	d2i9aa1	d2i9ab1	d2i9ac1
d2i9ba1	d2i9bb1	d2id3a1	d2id3b1	d2il8a_	d2il8b_	d2im8a1	d2im8b1
d2isya2	d2isyb2	d2isza2	d2iszc2	d2iszd2	d2it0a2	d2it0b2	d2it0c2
d2iw5b1	d2iy5a1	d2j00m1	d2j00o1	d2j00r1	d2j00s1	d2j01i1	d2j02m1
d2j02s1	d2j03i1	d2j28z1	d2j2ub1	d2j34b1	d2j38b1	d2j45a1	d2j45b1
d2j46b1	d2j4ib1	d2j7pa1	d2j7pb1	d2j7za1	d2j7zb1	d2j88h1	d2j94b1
d2j9ua1	d2j9uc1	d2j9va1	d2ja5f1	d2ja5i2	d2ja5j1	d2ja6f1	d2ja6j1
d2ja7i2	d2ja7j1	d2ja7r1	d2ja7u2	d2ja7v1	d2ja8f1	d2ja8j1	d2jaza1
d2jb0a1	d2jbga1	d2jbgc1	d2mhra_	d2newa_	d2ng1a1	d2nlla_	d2np5a1
d2np5c1	d2nu3i1	d2nu4i1	d2nvqf1	d2nvqj1	d2nvtf1	d2nvtj1	d2nvxf1
d2nvyj1	d2nvzf1	d2nvzj1	d2nyba1	d2nybb1	d2nybc1	d2nybd1	d2nzda1
d2o16a1	d2o16b2	d2o7oa1	d2o8ra1	d2o8rb1	d2ocya1	d2oiea1	d2oieb1
d2oied1	d2oiga1	d2oigb1	d2oigc1	d2oigd1	d2otl11	d2ovoa_	d2ow8d2
d2ow8p1	d2p06a1	d2p06b1	d2paca_	d2pf2a2	d2pfna1	d2pfoa1	d2pfoa2

Continued on Next Page...

Table 1 – Continued

**Domain Identifiers**							
d2pfqa2	d2phga1	d2phga2	d2plha_	d2proa2	d2proc1	d2proc2	d2pspa1
d2puaa1	d2puba1	d2puca1	d2puda1	d2puea1	d2pufa1	d2puga1	d2sdfa_
d2sgei_	d2sgfi_	d2sgpi_	d2sgqi_	d2sh1a_	d2sn3a_	d2snii_	d2soba_
d2tcta1	d2tdxa2	d2teci_	d2tgfa_	d2tpta1	d2trta1	d2utga_	d2utgb_
d2uu9s1	d2uuao1	d2uubo1	d2uubs1	d2uuco1	d2uucs1	d2uwlb1	d2uwob1
d2uxco1	d2uxcs1	d2uxnb1	d2yu9f1	d2yu9j1	d351ca_	d3alca_	d3bccf_
d3fisa_	d3fisb_	d3gcca_	d3hdhc1	d3icba_	d3il8a_	d3kiva_	d3mdsa1
d3ng1a1	d3ng1b1	d3nlaa_	d3ovoa_	d3pgha2	d3pghb2	d3pghc2	d3pghd2
d3rdna_	d3sgbi_	d3sgqi_	d3teci_	d3tgfa_	d451ca_	d4akea2	d4at1b2
d4coxb2	d4coxc2	d4coxd2	d4fapb_	d4fisa_	d4fisb_	d4hira_	d4mon.3
d4ovoa_	d4prch2	d4tgfa_	d5at1b2	d5coxa2	d5coxb2	d5coxc2	d5coxd2
d6coxb2	d7ceia_	d7icja3	d7icma3	d7icoa3	d7icsa3	d7prch2	d8icea3
