# Supplementary material for: Automatic structure classification of small proteins using random forest
Source: BMC Bioinformatics. 2010 Jul 1;11:364. doi: 10.1186/1471-2105-11-364 (PMC2916923; doi:10.1186/1471-2105-11-364)
Supplement: Additional file 7 — Domains consisting of 5SSEs from SCOP version 1.69. This file lists the identifiers for the 5SSEs containing domains from SCOP version 1.69. [file 1471-2105-11-364-S7.PDF]

Additional File 7

Domains consisting of 5SSEs from SCOP version 1.69

Table 1: Domains consisting of 5SSEs from SCOP version 1.69

Domain Identifiers							
d10gsa1	d10gsb1	d11gsa1	d11gsb1	d12gsa1	d12gsb1	d13gsa1	d13gsb1
d14gsa2	d14gsb1	d14gsb2	d155ca_	d16gsa1	d16gsb1	d17gsa1	d17gsb1
d18gsb1	d1914a2	d19gsa1	d19gsb1	d1a00a_	d1a03b_	d1a09b_	d1a0ba_
d1a0fb1	d1a0ob_	d1a0od_	d1a0of_	d1a0oh_	d1a1aa_	d1a1ba_	d1a1bb_
d1a1ea_	d1a1wa_	d1a26a1	d1a2ab_	d1a2ae_	d1a2ah_	d1a2va2	d1a2vb2
d1a2vd2	d1a2ve2	d1a2vf2	d1a3fa_	d1a3fb_	d1a3fc_	d1a57a_	d1a5la_
d1a5na_	d1a5ta1	d1a62a2	d1a64a_	d1a64b_	d1a67a_	d1a6aa2	d1a6ia2
d1a6pb_	d1a76a1	d1a77a1	d1a7bb_	d1a7bd_	d1a7ge_	d1a7ma_	d1a81g1
d1a8va2	d1a8vb2	d1a90a_	d1a9va_	d1aa0a_	d1aa1c_	d1aa1f_	d1aa1i_
d1aara_	d1aarb_	d1ab7a_	d1abva_	d1adna_	d1ae7a_	d1aewa_	d1afia_
d1ah5a2	d1ahka_	d1aipa2	d1aipb2	d1aipe2	d1aipf2	d1aisa1	d1am7b_
d1aoaa1	d1aoaa2	d1aofb1	d1aomb1	d1aono_	d1aonp_	d1aonq_	d1aons_
d1aonu_	d1aoqb1	d1aouf_	d1apja_	d1aqva1	d1aqvb1	d1aqwa1	d1aqwb1
d1aqwd1	d1aqxa2	d1aqxb1	d1aqxc1	d1aqxd2	d1arka_	d1at1b2	d1at1d2
d1au1b_	d1auss_	d1aust_	d1ausu_	d1ausv_	d1av1a_	d1av1c_	d1av4a2
d1avka2	d1avla2	d1avva_	d1aw0a_	d1aw9a2	d1awh.1	d1awh.2	d1axdb2
d1ay7a_	d1aypa_	d1aypc_	d1aypd_	d1aype_	d1aypf_	d1azpa_	d1azqa_
d1b1ga_	d1b1ua_	d1b23p2	d1b24a2	d1b27e_	d1b2ue_	d1b33f_	d1b34b_
d1b48a2	d1b48b2	d1b4aa1	d1b4ab1	d1b4ac1	d1b4ad1	d1b4ae1	d1b4af1
d1b4wb_	d1b4wc_	d1b4wd_	d1b59a1	d1b5la_	d1b64a_	d1b6aa1	d1b6ra2
d1b6sb2	d1b6sc2	d1b6sd2	d1b70b1	d1b70b2	d1b7fa2	d1b7fb2	d1b7yb1
d1b8aa1	d1b8ab1	d1b8ca_	d1b8ka_	d1b8la_	d1b8xa1	d1b8xa2	d1b8za_
d1b98a_	d1b9ma3	d1b9mb3	d1b9na3	d1b9nb3	d1baya1	d1baya2	d1bayb1
d1bbba_	d1bbbc_	d1bbr.3	d1bbxc_	d1bbxd_	d1bc6a_	d1bcga_	d1bcpd_
d1bcpj_	d1bcpl_	d1bd2d2	d1bd6a_	d1bdfa1	d1bdfb1	d1bdfc1	d1bdfd1
d1beyh2	d1bf4a_	d1bg0a1	d1bgca_	d1bgyc2	d1bgyd2	d1bgyo2	d1bgyp2
d1bhdb_	d1bhhb_	d1bhta1	d1bhtb1	d1bhua_	d1bija_	d1binb_	d1bipa_
d1bjjb_	d1bjjf_	d1bjpa_	d1bjpb_	d1bjpe_	d1bjya2	d1bjyb2	d1bjza2
d1bkba2	d1blua_	d1bm9a_	d1bm9b_	d1bmfa1	d1bmfa2	d1bmfb1	d1bmfb2
d1bmfd2	d1bmfe1	d1bmfe2	d1bmff1	d1bmff2	d1bn5a1	d1bnza_	d1boaa1
d1bosc_	d1bosd_	d1bose_	d1bosf_	d1bosi_	d1bosj_	d1bosk_	d1bosl_
d1boso_	d1bosp_	d1bosq_	d1bosr_	d1boss_	d1bp3b2	d1bqxa_	d1bs2a1
d1bu2a1	d1buwc_	d1bvnt_	d1bwea_	d1bwoa_	d1bx2a2	d1bx9a2	d1bxea_
d1by4d_	d1by9a_	d1byea2	d1byeb2	d1c02a_	d1c02b_	d1c03a_	d1c03b_
d1c03d_	d1c07a_	d1c0aa1	d1c1ja_	d1c1jb_	d1c1jc_	d1c1jd_	d1c1yb_
d1c2aa2	d1c3ga1	d1c3ta_	d1c3ya_	d1c48e_	d1c5o.1	d1c74a_	d1c7va_
d1c7ya2	d1c7ya3	d1c8aa2	d1c8ca_	d1c9ba2	d1c9bb1	d1c9bb2	d1c9be2
d1c9bf2	d1c9bi2	d1c9bj1	d1c9bm2	d1c9bn1	d1c9bn2	d1c9bq2	d1c9br1
d1c9th_	d1c9ti_	d1c9tj_	d1c9tk_	d1c9tl_	d1ca5a_	d1ca6a_	d1cb1a_
d1cc8a_	d1cd9a_	d1cd9c_	d1cdca_	d1cdcb_	d1cdna_	d1cdqa_	d1cdra_
d1cdya2	d1cf4a_	d1cf7a_	d1cg5a_	d1cg7a_	d1cg8a_	d1cgna_	d1cgoa_
d1ckla2	d1cklb2	d1ckld2	d1cklf2	d1cksa_	d1cksb_	d1cksc_	d1cl5a_

Continued on Next Page...

Table 1 – Continued

**Domain Identifiers**							
d1clba_	d1cmcb_	d1cmia_	d1cmib_	d1cmxb_	d1cnpb_	d1co0a_	d1coma_
d1comc_	d1comd_	d1come_	d1comf_	d1comg_	d1comh_	d1comi_	d1comj_
d1coml_	d1copd_	d1cope_	d1cowa1	d1cowa2	d1cowb1	d1cowb2	d1cowc2
d1cowd2	d1cowe2	d1cowf2	d1cpza_	d1cqma_	d1cqmb_	d1cqna_	d1cqnb_
d1cqua_	d1cqxa1	d1cqxb1	d1crkb1	d1crkc1	d1csqa_	d1csya_	d1csza_
d1cuka3	d1cx8a1	d1cx8b1	d1cx8c1	d1cx8d1	d1cx8e1	d1cx8f1	d1cx8g1
d1cyua_	d1czgd_	d1czwa_	d1czwc_	d1czwe_	d1czwf_	d1czwg_	d1czwh_
d1d09b2	d1d09d2	d1d1la_	d1d1ma_	d1d1mb_	d1d1oa_	d1d1ra_	d1d2za_
d1d3ba_	d1d3bc_	d1d3bd_	d1d3be_	d1d3bg_	d1d3bi_	d1d3bj_	d1d3bk_
d1d3ub1	d1d3wa_	d1d3za_	d1d4ua1	d1d5ma2	d1d5va_	d1d5xa2	d1d5za2
d1d6ua3	d1d6ub2	d1d6ya2	d1d6ya3	d1d6yb2	d1d6yb3	d1d6za2	d1d6za3
d1d7qa_	d1d8ja_	d1d8lb2	d1d9aa_	d1dara3	d1daxa_	d1db4a_	d1db5a_
d1dcja_	d1dcya_	d1dd3a2	d1dd3b2	d1dd4a2	d1dd4b2	d1ddfa_	d1de4c1
d1de4i1	d1de7.1	d1dfda_	d1dg1g2	d1dgja1	d1dgna_	d1dgsa2	d1dgsb2
d1dhmb_	d1djta_	d1djtb_	d1dkga1	d1dkgb1	d1dkta_	d1dktb_	d1dlhd2
d1dlpc1	d1dm0d_	d1dm0e_	d1dm0f_	d1dm0j_	d1dowa_	d1dp7p_	d1dpua_
d1dt4a_	d1dt7a_	d1dt7b_	d1dtdb_	d1dtja_	d1dtjc_	d1dtjd_	d1dtqa1
d1du7a2	d1dvka_	d1dvkb_	d1dwb.1	d1dwc.1	d1dwd.1	d1dwe.1	d1dx5.2
d1dyua2	d1dyua3	d1dyub2	d1dyub3	d1dzfa2	d1e0f.1	d1e1pa_	d1e1qa1
d1e1qb1	d1e1qb2	d1e1qc2	d1e1qd1	d1e1qd2	d1e1qe2	d1e1qf2	d1e1ra1
d1e1rb1	d1e1rb2	d1e1rc2	d1e1rd2	d1e1re1	d1e1re2	d1e1rf2	d1e1wa_
d1e2xa1	d1e3ha6	d1e3pa7	d1e3ya_	d1e41a_	d1e44a_	d1e44b_	d1e5wa3
d1e79a1	d1e79a2	d1e79b1	d1e79c2	d1e79d1	d1e79d2	d1e79e1	d1e79e2
d1e79f2	d1e7da2	d1e83a_	d1e84a_	d1e85a_	d1e86a_	d1e88a2	d1e8aa_
d1e8oa_	d1e9hb1	d1e9hd1	d1e9hd2	d1e9ya2	d1e9za2	d1ea9d2	d1eara2
d1eayd_	d1eb0a2	d1ec6a_	d1ec6b_	d1eema2	d1ef1a1	d1ef1a3	d1ef1b1
d1ef2c_	d1efca2	d1efra1	d1efra2	d1efrb1	d1efrb2	d1efrc2	d1efrd2
d1efrf1	d1efrf2	d1efta2	d1efub4	d1efud4	d1efya1	d1egcb1	d1egcc1
d1eh6a1	d1eh7a1	d1eh8a1	d1eifa1	d1eiya1	d1eiyb1	d1ej7s_	d1ejra_
d1ejta_	d1ejua_	d1ejva_	d1ejwa_	d1ejxa_	d1ekma2	d1ekmb2	d1ekmc2
d1ekub1	d1ekub2	d1elva2	d1emwa_	d1eoga1	d1eogb1	d1eoha1	d1eohb1
d1eohd1	d1eohe1	d1eohf1	d1eohg1	d1eohh1	d1eq1a_	d1eqfa1	d1eqfa2
d1eqra1	d1eqrc1	d1es7b_	d1es7d_	d1etea_	d1eteb_	d1etec_	d1eted_
d1etob_	d1etqa_	d1etqb_	d1etqd_	d1etwb_	d1euic_	d1euid_	d1euvb_
d1ev9c1	d1ewra1	d1exka_	d1exma2	d1exza_	d1exzb_	d1exzc_	d1exzd_
d1eyfa_	d1ezga_	d1ezgb_	d1ezvc2	d1f04a_	d1f1bd2	d1f2ea1	d1f2eb1
d1f2rc_	d1f36a_	d1f36b_	d1f3cb_	d1f3ja2	d1f3jd2	d1f4ka_	d1f4kb_
d1f54a_	d1f55a_	d1f5ba_	d1f5ca_	d1f60b_	d1f6fa_	d1f70a_	d1f71a_
d1f7va1	d1f94a_	d1f95a_	d1f95b_	d1f96a_	d1f96b_	d1f9fa_	d1f9fb_
d1f9fd_	d1f9ja_	d1f9jb_	d1f9na1	d1f9nb1	d1f9nc1	d1f9nd1	d1f9ne1
d1fa0a3	d1fa3a_	d1faih2	d1fawa_	d1fawc_	d1fb2a_	d1fb2b_	d1fbiy_
d1fbza_	d1fc5a1	d1fc5b1	d1fcba2	d1fe0a_	d1fe0b_	d1fe4a_	d1fe4b_
d1feeb_	d1ff2a_	d1ffgb_	d1ffgd_	d1ffsb_	d1ffsd_	d1ffua2	d1ffud2
d1ffvd2	d1ffwb_	d1ffwd_	d1fg9a_	d1fg9b_	d1fgpa_	d1fhaa_	d1fhja_
d1fi5a_	d1fi6a_	d1finb1	d1find1	d1fioa_	d1fiqa2	d1fjgc1	d1fjgc2
d1fjgq_	d1fkaf_	d1fkag_	d1fkqa_	d1fl7b_	d1fmms_	d1fnka_	d1fnma3
d1fo4a2	d1fo4b2	d1foha4	d1foka3	d1fowa_	d1fph.1	d1fqvb2	d1fqvd2

Continued on Next Page…

Table 1 – Continued

Domain Identifiers

d1fqvh2	d1fqvj2	d1fqvl2	d1fqvn2	d1fqvp2	d1fs1d2	d1fs2b2	d1fs2d2
d1fv0b_	d1fvvb1	d1fvvd1	d1fw4a_	d1fx0a1	d1fx0a2	d1fx0b2	d1fxkc_
d1fyha1	d1fyha2	d1fyhd1	d1fyhd2	d1fyra_	d1fyta2	d1fytd2	d1fzpb_
d1fzva_	d1fzvb_	d1g0aa_	d1g18a2	d1g19a2	d1g1xf_	d1g2ea2	d1g2ra_
d1g30.1	d1g32.1	d1g3oa_	d1g3pa1	d1g40a1	d1g40a2	d1g40a3	d1g40a4
d1g40b2	d1g40b3	d1g40b4	d1g44a3	d1g44a4	d1g44b3	d1g44c4	d1g4fa_
d1g4wr1	d1g51a1	d1g51b1	d1g6ba_	d1g6ja_	d1g6rd1	d1g7cb_	d1g8ea_
d1g8lb1	d1g8ra1	d1g8rb1	d1g96a_	d1ga3a_	d1gaka_	d1gaob_	d1gaod_
d1gc6a3	d1gc7a1	d1gc7a3	d1gd4a_	d1gdia_	d1gdja_	d1gdka_	d1gdla_
d1gg3b3	d1gg3c3	d1gh4a_	d1ghha_	d1ghka_	d1ghta_	d1ghv.1	d1gjqa1
d1gjua1	d1gjwa1	d1gjxa_	d1gk8i_	d1gk8k_	d1gk8m_	d1gk8o_	d1gkga1
d1glpa1	d1glpb1	d1glqa1	d1glqb1	d1gm4a_	d1gmba_	d1gmna1	d1gmoe1
d1gmpa_	d1gmqa_	d1gmua2	d1gmub2	d1gmuc2	d1gmud2	d1gmva2	d1gmvb2
d1gmwb2	d1gmwc2	d1gmwd2	d1gmza_	d1gmzb_	d1gnka_	d1gnkb_	d1gnwa2
d1go3f_	d1go3n_	d1gp9a1	d1gp9b1	d1gp9c1	d1gp9d1	d1gpma3	d1gpmb3
d1gpza2	d1gpza3	d1gpzb3	d1gq1a1	d1gq1b1	d1gqma_	d1gqmc_	d1gqmg_
d1gqmj_	d1grja2	d1gruo_	d1grup_	d1gruq_	d1grus_	d1grut_	d1gruu_
d1gs0b1	d1gsda1	d1gsdb1	d1gsdc1	d1gsdd1	d1gsqa1	d1gssa1	d1gssb1
d1gtaa1	d1gtba1	d1gtia1	d1gtib1	d1gtib2	d1gtic1	d1gtid1	d1gtie1
d1gtka2	d1guab_	d1gvha1	d1gvib2	d1gwga_	d1gy3d1	d1h03p1	d1h03p2
d1h03q2	d1h04p1	d1h04p2	d1h0xa_	d1h0xb_	d1h0ya_	d1h15a2	d1h15d2
d1h1pd1	d1h1qb1	d1h1qd1	d1h1rb1	d1h1sb1	d1h1sd1	d1h24b1	d1h24d1
d1h25d1	d1h25d2	d1h26b1	d1h26d1	d1h27b1	d1h27d1	d1h28b2	d1h28d1
d1h2qp2	d1h3na1	d1h4ld_	d1h4le_	d1h4qa3	d1h4qb3	d1h4ra1	d1h4ra3
d1h4rb3	d1h4sa3	d1h4ta3	d1h4tb3	d1h4tc3	d1h4td3	d1h641_	d1h642_
d1h64b_	d1h64c_	d1h64d_	d1h64e_	d1h64f_	d1h64g_	d1h64h_	d1h64i_
d1h64k_	d1h64l_	d1h64m_	d1h64n_	d1h64o_	d1h64p_	d1h64q_	d1h64r_
d1h64t_	d1h64u_	d1h64v_	d1h64w_	d1h64x_	d1h64y_	d1h64z_	d1h67a_
d1h8ea2	d1h8eb1	d1h8eb2	d1h8ec2	d1h8ed1	d1h8ed2	d1h8ee2	d1h8ef1
d1h8ha1	d1h8ha2	d1h8hb1	d1h8hb2	d1h8hc2	d1h8hd2	d1h8he2	d1h8hf1
d1h96a_	d1h97a_	d1h97b_	d1h9ga1	d1h9ra1	d1h9rb1	d1h9sa1	d1h9sb1
d1ha3b2	d1haba_	d1habc_	d1haea_	d1hafa_	d1hah.1	d1hbxh_	d1hc1a1
d1hc3a1	d1hc4a1	d1hc5a1	d1hc6a1	d1hc7a3	d1hc7b3	d1hc7c3	d1hc7d3
d1hc9b_	d1hcfb_	d1hcob_	d1hcya1	d1hcyb1	d1hcyc1	d1hcyd1	d1hcye1
d1hd0a_	d1hd1a_	d1hehc_	d1hf8a2	d1hfaa2	d1hfha1	d1hfia_	d1hfoc_
d1hfyb_	d1hg2a2	d1hg5a2	d1hgbc_	d1hgua_	d1hh2p3	d1hh5a_	d1hi7a_
d1hia.2	d1hica_	d1hj5a1	d1hj5b1	d1hjpa3	d1hk9a_	d1hk9b_	d1hk9c_
d1hk9e_	d1hk9f_	d1hkia2	d1hkya_	d1hlma_	d1hlt.2	d1hnaa1	d1hnbb1
d1hncb1	d1hncc1	d1hncd1	d1hnwc1	d1hnwc2	d1hnwf_	d1hnwl_	d1hnwm_
d1hnwq_	d1hnxc1	d1hnxc2	d1hnxj_	d1hnxp_	d1hnxq_	d1hnzc2	d1hnzf_
d1hnzm_	d1hnzp_	d1hnzq_	d1hoea_	d1hq6.1	d1hq6.2	d1hr0c2	d1hr0m_
d1hr0w_	d1hrpb_	d1hrsa_	d1hsta_	d1hstb_	d1huua_	d1huub_	d1huuc_
d1hv4c_	d1hv4e_	d1hv4g_	d1hw1a1	d1hw1b1	d1hw2a1	d1hw2b1	d1hw5a1
d1hwuc_	d1hwud_	d1hwue_	d1hwuf_	d1hx5a_	d1hx5b_	d1hx5c_	d1hx5d_
d1hx5f_	d1hx5g_	d1hx7a_	d1hx8a2	d1hx8b2	d1hxda1	d1hxdb1	d1hxf.1
d1hz6a_	d1hz6b_	d1hz6c_	d1hzia_	d1i0ea1	d1i0eb1	d1i0ec1	d1i0ed1
d1i17a_	d1i1ga2	d1i1gb2	d1i27a_	d1i3ja_	d1i3qe2	d1i3qk_	d1i42a_

Continued on Next Page…

Domain Identifiers							
d1i4k1_	d1i4k2_	d1i4ka_	d1i4kb_	d1i4kc_	d1i4kd_	d1i4ke_	d1i4kf_
d1i4ki_	d1i4kj_	d1i4kk_	d1i4kl_	d1i4km_	d1i4kn_	d1i4kp_	d1i4kq_
d1i4ks_	d1i4kt_	d1i4kv_	d1i4kw_	d1i4kx_	d1i4kz_	d1i4lb_	d1i4ma_
d1i50c1	d1i50e2	d1i50k_	d1i56a_	d1i5la_	d1i5lb_	d1i5lc_	d1i5ld_
d1i5lf_	d1i5lg_	d1i5lh_	d1i5li_	d1i5lk_	d1i5ll_	d1i5lm_	d1i5ln_
d1i6he2	d1i6hk_	d1i6va1	d1i6va2	d1i77a_	d1i81a_	d1i81b_	d1i81c_
d1i81e_	d1i81f_	d1i87a_	d1i8fa_	d1i8fb_	d1i8fc_	d1i8fd_	d1i8fe_
d1i8fg_	d1i8va_	d1i94c1	d1i94c2	d1i94e1	d1i94f_	d1i94g_	d1i94j_
d1iaka2	d1iaoa2	d1iara_	d1ib8a1	d1ibkm_	d1ibkp_	d1ibkq_	d1iblf_
d1iblq_	d1ibml_	d1ibmm_	d1ic8a2	d1icha_	d1ie7a_	d1iera_	d1iesa_
d1iesc_	d1iese_	d1if1a_	d1if1b_	d1ig5a_	d1ig6a_	d1igva_	d1ih0a_
d1ijeb_	d1ijfb_	d1ijra_	d1ijxa_	d1ijxc_	d1ijxd_	d1ijya_	d1ijyb_
d1ik0a_	d1il2a1	d1il2b1	d1il6a_	d1imua_	d1io5a_	d1ioka3	d1iokb3
d1iokd3	d1ioke3	d1iokf3	d1iokg3	d1iova1	d1iowa1	d1ip9a_	d1ipga_
d1iqcb1	d1iqcc1	d1iqcd1	d1iqoa_	d1iqsa_	d1iqta_	d1iqxa2	d1iqxb2
d1iqyb2	d1iqza_	d1ir0a_	d1ir1s_	d1ir1t_	d1ir1u_	d1ir1v_	d1ir21_
d1ir23_	d1ir24_	d1ir25_	d1ir26_	d1ir27_	d1ir28_	d1ir2i_	d1ir2j_
d1ir2l_	d1ir2m_	d1ir2n_	d1ir2o_	d1ir2p_	d1irba_	d1irga_	d1is2a1
d1isna3	d1it2a_	d1it2b_	d1it3a_	d1it3b_	d1it3d_	d1itfa_	d1itia_
d1itma_	d1itpa_	d1iu7a2	d1iu7b2	d1iuya_	d1iv7a_	d1iv7b_	d1iv9a_
d1ivua2	d1ivub2	d1ivva2	d1ivvb2	d1ivwa2	d1ivwb2	d1ivxa2	d1ivxb2
d1iw7b1	d1iw7f2	d1iw7k1	d1iw7l1	d1iw7p2	d1ixcb1	d1ixra2	d1ixrb3
d1iyhb1	d1iyhc1	d1iyhd1	d1iyia1	d1iyib1	d1iyic1	d1iyid1	d1iyya_
d1iz1b1	d1iz1p1	d1iz1q1	d1iz6a1	d1iz6a2	d1iz6b1	d1iz6b2	d1iz6c1
d1j0ga_	d1j0ra_	d1j0rb_	d1j19a1	d1j19a3	d1j1va_	d1j27a_	d1j2xa_
d1j4wa2	d1j55a_	d1j5ec2	d1j5em_	d1j5eq_	d1j5ka_	d1j72a3	d1j75a_
d1j8ca_	d1j8ha2	d1j9ib_	d1jb0c_	d1jbma_	d1jbmb_	d1jbmc_	d1jbmd_
d1jbmf_	d1jbmg_	d1jc2a_	d1jchb_	d1jchd_	d1jdqa_	d1je3a_	d1jebc_
d1jgka_	d1jgna_	d1jhga_	d1jhwa3	d1jiaa_	d1jiab_	d1jica_	d1jiha1
d1jj2w_	d1jj2x_	d1jj4a_	d1jj4b_	d1jjcb1	d1jjcb2	d1jjcb4	d1jjga_
d1jjhb_	d1jjhc_	d1jk9b2	d1jk9d2	d1jkwa1	d1jl4a2	d1jlva1	d1jlvb1
d1jlvd1	d1jlve1	d1jlvf1	d1jlwa1	d1jlwb1	d1jnda2	d1jnea2	d1josa_
d1jq8a_	d1jq8b_	d1jq9a_	d1jq9b_	d1jqjc1	d1jqjd1	d1jr3a1	d1jr3b1
d1jr3e1	d1jr5a_	d1jr5b_	d1jrhl2	d1jrib_	d1jric_	d1jrid_	d1jrie_
d1jrig_	d1jrih_	d1jrii_	d1jrij_	d1jrik_	d1jril_	d1jrim_	d1jrin_
d1jroc2	d1jroe2	d1jrog2	d1jrpa2	d1jrpc2	d1jrpe2	d1jrpg2	d1jrqa2
d1jrqb2	d1jrqb3	d1jstd1	d1jsub1	d1jt0d2	d1jtyb2	d1jumb2	d1jupb2
d1jwea_	d1jwua2	d1jwwa_	d1jx4a1	d1jxla1	d1jxma1	d1jzda2	d1k0na1
d1k0oa1	d1k0ob1	d1k0ra1	d1k0ra3	d1k0rb1	d1k0rb3	d1k0rb4	d1k0va_
d1k25b1	d1k25c2	d1k25d1	d1k2da2	d1k3oa1	d1k3ob1	d1k50a_	d1k73y_
d1k83c1	d1k83e2	d1k83k_	d1k8ar_	d1k8ay_	d1k8az_	d1k8ba_	d1k8ga2
d1k8ua_	d1k98a1	d1k9ab2	d1k9ac2	d1k9af2	d1k9ka_	d1k9mr_	d1k9my_
d1k9pa_	d1ka8e_	d1ka8f_	d1kb4b_	d1kb9c1	d1kbaa_	d1kbna1	d1kbnb1
d1kc8z_	d1kcya_	d1kd1r_	d1kd1y_	d1kd1z_	d1kd2a_	d1kdua_	d1kf9a_
d1kfha_	d1kg0a2	d1kgcd2	d1khia2	d1khma_	d1kirc_	d1kiui2	d1kiuk2
d1kiuo2	d1kjza2	d1kk0a2	d1kk1a2	d1kk2a2	d1kk3a2	d1kkeb1	d1kkec1
d1klga2	d1kloa1	d1klua2	d1kmha1	d1kmha2	d1kmhb2	d1kn6a_	d1knya1

Continued on Next Page…

Domain Identifiers

d1kpma_	d1kpmb_	d1kq0a1	d1kq1a_	d1kq1b_	d1kq1h_	d1kq1i_	d1kq1k_
d1kq1n_	d1kq1r_	d1kq1s_	d1kq1t_	d1kq1w_	d1kq1y_	d1kq2a_	d1kq2b_
d1kq2i_	d1kq2k_	d1kq2m_	d1kq9a1	d1kqsp_	d1kqsw_	d1kqsx_	d1kqua_
d1krba_	d1krqa_	d1ks2a2	d1ks2b2	d1ks9a1	d1ksia2	d1ksib2	d1ksib3
d1ksla3	d1ksma_	d1ksoa_	d1ksob_	d1ksqa_	d1ksva3	d1kt0a2	d1kt1a2
d1kv0b_	d1kvia_	d1kvja_	d1kvna_	d1kvoa_	d1kvob_	d1kvoc_	d1kvod_
d1kvwa_	d1kvya_	d1kwga1	d1kwja_	d1kwka1	d1kx8a_	d1kx9a_	d1kx9b_
d1kxua2	d1ky9a1	d1kyoc2	d1kyog_	d1kyon2	d1kyor_	d1l0ba2	d1l0ld1
d1l0wa1	d1l0wb1	d1l2fa1	d1l2fa3	d1l3wa5	d1l4sa_	d1l5gb3	d1l6na1
d1l8qa1	d1la2d2	d1laba_	d1lb3a_	d1lbka1	d1lbkb1	d1lcja_	d1ldda_
d1ldja1	d1ldjb_	d1lflr_	d1lfqa_	d1lfta_	d1lfza_	d1lh1a_	d1lh2a_
d1lh5a_	d1lh6a_	d1lh7a_	d1ljoa_	d1lk5a2	d1lk5b2	d1lk5c2	d1lk5d2
d1lk7b2	d1lk7c2	d1lk7d2	d1lkka_	d1lkla_	d1lkza2	d1lkzb2	d1lm0a_
d1lmsa_	d1ln0a_	d1ln0b_	d1lnlc2	d1lnqg2	d1lnqh2	d1lnua2	d1lnuc2
d1lnug2	d1lnxa_	d1lnxb_	d1lnxc_	d1lnxd_	d1lnxe_	d1lnxf_	d1lnxg_
d1loja_	d1lojb_	d1lojc_	d1lojd_	d1loje_	d1lojf_	d1lojg_	d1lojh_
d1lojj_	d1lojk_	d1lojl_	d1lojm_	d1lojn_	d1loua_	d1lp9e2	d1lq1a_
d1lq1c_	d1lq1d_	d1lqba_	d1lqgc_	d1lqgd_	d1ltxa3	d1luma_	d1luza_
d1lvaa1	d1lvaa2	d1lvna2	d1lvna3	d1lvnb2	d1lvnb3	d1lwha1	d1lwhb1
d1lwjb1	d1lwsa2	d1lx8a_	d1lyw.1	d1lyw.2	d1lyw.3	d1lyw.4	d1lzwa_
d1m0sb2	d1m0ua1	d1m0ub1	d1m15a1	d1m1je2	d1m1jf2	d1m1kr_	d1m1ky_
d1m2vb5	d1m47a_	d1m48a_	d1m48b_	d1m49a_	d1m49b_	d1m4aa_	d1m4ba_
d1m4cb_	d1m4rb_	d1m5hd2	d1m5hf2	d1m5sa2	d1m6ba4	d1m6na2	d1m6ya1
d1m74a2	d1m80a1	d1m80b1	d1m8va_	d1m8vb_	d1m8vc_	d1m8vd_	d1m8ve_
d1m8vg_	d1m8vh_	d1m8vi_	d1m8vj_	d1m8vk_	d1m8vl_	d1m8vm_	d1m8vn_
d1m90y_	d1m90z_	d1maba1	d1mabb1	d1mb8a2	d1mbuc_	d1mbud_	d1mbvb_
d1mbxd_	d1mc7a_	d1mc8b1	d1md3a1	d1md3b1	d1md4a1	d1md4b1	d1md7a2
d1mdab_	d1mf4a_	d1mfqb_	d1mfra_	d1mfrb_	d1mfrc_	d1mfrd_	d1mfre_
d1mfrg_	d1mfrh_	d1mfri_	d1mfrj_	d1mfrk_	d1mfrl_	d1mfrm_	d1mfrn_
d1mfrp_	d1mfrq_	d1mfrr_	d1mfrs_	d1mfrt_	d1mfru_	d1mfrv_	d1mfrw_
d1mg2d_	d1mg2h_	d1mg2l_	d1mg2p_	d1mg3d_	d1mg3h_	d1mg3l_	d1mg3p_
d1mgqa_	d1mgqb_	d1mgqc_	d1mgqd_	d1mgqe_	d1mgqf_	d1mgqg_	d1mgra_
d1mgwa_	d1mh2a_	d1mh2b_	d1mi5d2	d1mifc_	d1mija_	d1mjda_	d1mjea4
d1mkma1	d1mkmb1	d1mkoc_	d1mkta_	d1mlaa2	d1mlce_	d1mmfg_	d1mmfm_
d1mo1a_	d1mo1b_	d1mo1c_	d1mo1d_	d1mo3a2	d1mo4a2	d1mo5a2	d1mo6a2
d1molb_	d1mp9a1	d1mp9b1	d1mqlb_	d1mu4a_	d1mu4b_	d1mula_	d1muza_
d1mv3a1	d1mw4a_	d1mwaa2	d1mwad2	d1mwna_	d1mwnb_	d1mwya_	d1n0qa_
d1n0ua4	d1n0vc4	d1n0vd4	d1n0ya_	d1n0yb_	d1n15a1	d1n28a_	d1n28b_
d1n2ab1	d1n2ab2	d1n2xa1	d1n2xb1	d1n32c1	d1n32c2	d1n32f_	d1n32j_
d1n32m_	d1n32q_	d1n33c2	d1n33m_	d1n34e1	d1n34f_	d1n34m_	d1n36m_
d1n3ka_	d1n48a1	d1n50a1	d1n50b1	d1n56a1	d1n56b1	d1n5ox2	d1n5wa2
d1n5xa2	d1n5xb2	d1n60a2	d1n60d2	d1n61a2	d1n61d2	d1n62a2	d1n62d2
d1n63d2	d1n6ua2	d1n6va2	d1n6za_	d1n8rr_	d1n8ry_	d1n8rz_	d1n8ua_
d1n8vb_	d1n90a1	d1n90b1	d1n9da_	d1n9ra_	d1n9rb_	d1n9rc_	d1n9rd_
d1n9rf_	d1n9rg_	d1n9sa_	d1n9sb_	d1n9sc_	d1n9sd_	d1n9se_	d1n9sf_
d1n9sh_	d1n9si_	d1n9sj_	d1n9sk_	d1n9sl_	d1n9sm_	d1n9sn_	d1naml_
d1nbfd_	d1nbma1	d1nbma2	d1nbmb2	d1nbmc2	d1nbmd2	d1nbme1	d1nbme2

Continued on Next Page…

Domain Identifiers							
d1nbmf2	d1nbpa_	d1ndda_	d1nddb_	d1nddc_	d1nddd_	d1neea1	d1nexa2
d1nfdc2	d1nfha_	d1nfhb_	d1nfja_	d1ngmm1	d1ngmm2	d1ngra_	d1nh7a2
d1nh9a_	d1nhoa_	d1ni2a1	d1ni2a3	d1ni2b1	d1ni2b3	d1niki_	d1nikk_
d1nirb1	d1nj1a2	d1nj2a2	d1nj5a2	d1nj6a2	d1nj8a2	d1nj8b2	d1nj8c2
d1njiy_	d1njiz_	d1nk1a1	d1nk1b1	d1nkta2	d1nktb2	d1nl3a2	d1nl3b2
d1nm2a2	d1nnva_	d1nnxa_	d1npeb1	d1npqa_	d1nqlb_	d1nrr.1	d1ntna_
d1nwva2	d1nxia_	d1nyqb2	d1nzna_	d1nzq.1	d1o0d.1	d1o3ta1	d1o3wa_
d1o50a1	d1o57b1	d1o57c1	d1o5ha_	d1o5hb_	d1o6ab_	d1o6sa1	d1o6ta1
d1o6vb1	d1o75a2	d1o78a_	d1o7la2	d1o7lb2	d1o7lc2	d1o7ld2	d1o82a_
d1o82c_	d1o82d_	d1o83a_	d1o83b_	d1o83c_	d1o83d_	d1o8ba2	d1o8bb2
d1o90a1	d1o90a3	d1o90b1	d1o90b3	d1o92a3	d1o92b1	d1o92b3	d1o93a3
d1o93b3	d1o9aa2	d1o9sb1	d1o9ta3	d1o9tb1	d1o9tb3	d1oaca2	d1oaca3
d1oacb3	d1ob2a2	d1ob5a2	d1ob5c2	d1ob5e2	d1obca1	d1occe_	d1occr_
d1odbe_	d1oe7a1	d1oe7b1	d1oe8a1	d1oe8b1	d1ofcx2	d1ogub1	d1ogud1
d1ohha2	d1ohhb1	d1ohhb2	d1ohhc2	d1ohhd2	d1ohhe1	d1ohhe2	d1ohhf2
d1oi9d1	d1oiub1	d1oiud1	d1oiyb1	d1oiyd1	d1oiyd2	d1oj6d_	d1ojva3
d1ojvb1	d1ojvb3	d1ojvb4	d1ojwa1	d1ojwa3	d1ojwa4	d1ojwb1	d1ojwb3
d1ojya3	d1ojya4	d1ojyb3	d1ojyb4	d1ojyc3	d1ojyc4	d1ojyd3	d1ojyd4
d1ok1a3	d1ok1a4	d1ok1b1	d1ok1b3	d1ok1b4	d1ok2a3	d1ok2a4	d1ok2b1
d1ok2b4	d1ok3a3	d1ok3a4	d1ok3b1	d1ok3b3	d1ok3b4	d1ok9a3	d1ok9a4
d1ok9b3	d1okga3	d1okta1	d1oktb1	d1okvb1	d1okvd1	d1okwd1	d1ol1b1
d1ol2b1	d1ol2d1	d1olza1	d1omsa_	d1omsb_	d1omsc_	d1omya_	d1oofa_
d1ooga_	d1oogb_	d1ooha_	d1oohb_	d1ooix_	d1ook.1	d1op5k2	d1opza_
d1oq6a_	d1oqpa_	d1oqya4	d1orga_	d1orgb_	d1orka2	d1osda_	d1osdb_
d1ovzb2	d1ow0c2	d1ow4b_	d1owsa_	d1owta_	d1oxba_	d1oxka_	d1oxkc_
d1oxkg_	d1oxki_	d1oxkk_	d1oxla_	d1oxlb_	d1oy8a3	d1oyda4	d1oyfa_
d1oypa2	d1oypb2	d1oypc2	d1oypd2	d1oype2	d1oypf2	d1oyra2	d1oyrb2
d1oyrd2	d1oyre2	d1oyrf2	d1oysa2	d1oyvi_	d1oz6a_	d1ozoa_	d1ozsa_
d1p1aa_	d1p1fb2	d1p1ib2	d1p27b_	d1p27d_	d1p28a_	d1p28b_	d1p3he_
d1p3hi_	d1p3hl_	d1p3qu_	d1p3qv_	d1p4aa1	d1p4ab1	d1p4ac1	d1p4ad1
d1p52a1	d1p53b1	d1p5eb1	d1p5ed1	d1p5qa2	d1p5qb2	d1p68a_	d1p6ra_
d1p6zn_	d1p6zr_	d1p7oa_	d1p7ob_	d1p7oc_	d1p7od_	d1p7oe_	d1p7of_
d1p84g_	d1p8ca_	d1p8cb_	d1p8cc_	d1p8cd_	d1p8ce_	d1p8cf_	d1p8ga_
d1p8xa3	d1p98a_	d1p9du_	d1p9ka_	d1p9mb_	d1p9mc1	d1p9qc3	d1p9ya_
d1pa0a_	d1pa3a1	d1pa3b1	d1paxa1	d1pbia_	d1pbja1	d1pbja2	d1pc0a_
d1pc4a_	d1pc5a_	d1pc6b_	d1pc9a_	d1pc9b_	d1pcfb_	d1pcfc_	d1pcfd_
d1pcff_	d1pcfg_	d1pcfh_	d1pcxa5	d1pcza1	d1pczb2	d1pd1a5	d1pd211
d1pdaa2	d1pdgc_	d1pf9o_	d1pf9p_	d1pf9q_	d1pf9r_	d1pf9s_	d1pf9t_
d1pfsa_	d1pfsb_	d1pgrd2	d1pgrh2	d1pgta1	d1pgtb1	d1phza1	d1pisa_
d1pj7a1	d1pjwa_	d1pkdb1	d1pkdd1	d1pkna1	d1pkpa1	d1pksa_	d1pkta_
d1pkzb1	d1pm3a_	d1pm3b_	d1pmta1	d1pn0a3	d1pn0b3	d1pn0c3	d1pn0d3
d1pn9b1	d1pnsm_	d1pnsq_	d1pnxm_	d1pnxq_	d1po8a_	d1poaa_	d1poba_
d1poda_	d1poea_	d1pp2l_	d1pp2r_	d1pp9p1	d1ppaa_	d1ppjc1	d1ppjp1
d1pqxa_	d1prtd_	d1prte_	d1prtf_	d1prtj_	d1prtk_	d1prtl_	d1psba_
d1psda3	d1psdb3	d1ptof_	d1ptol_	d1puga_	d1pv4a2	d1pv4b1	d1pv4c2
d1pv4e2	d1pv4f2	d1pvab_	d1pvba_	d1pvma2	d1pvmb2	d1pvoa2	d1pvod2
d1pvof2	d1pw6a_	d1pw6b_	d1pwja_	d1pwka_	d1px6a1	d1px6b1	d1px7a1

Continued on Next Page…

Table 1 – Continued

Domain Identifiers

d1py2a_	d1py2b_	d1py2d_	d1py3b_	d1pysb1	d1pysb2	d1pysb4	d1pywa2
d1q2za_	d1q3sa3	d1q3sc3	d1q3sd3	d1q3se3	d1q3sf3	d1q3sg3	d1q3sh3
d1q3xb2	d1q4ja1	d1q4jb1	d1q5vd2	d1q5ya_	d1q5yb_	d1q5yc_	d1q5yd_
d1q6va_	d1q7aa_	d1q7ja_	d1q7yy_	d1q7yz_	d1q81y_	d1q81z_	d1q82r_
d1q86r_	d1q86y_	d1q86z_	d1q8la_	d1q8rb_	d1q95h2	d1qafa2	d1qafa3
d1qafb3	d1qaga1	d1qaga2	d1qagb1	d1qagb2	d1qaka2	d1qaka3	d1qakb2
d1qala2	d1qala3	d1qalb2	d1qalb3	d1qb2b_	d1qbjc_	d1qbv.1	d1qfwa_
d1qh4b1	d1qh4c1	d1qj1.1	d1qj6.1	d1qjha_	d1qk1a1	d1qk1b1	d1qk1c1
d1qk1f1	d1qk1g1	d1qk1h1	d1qksa1	d1qksb1	d1qlla_	d1qllb_	d1qm4a1
d1qm4b1	d1qm4b3	d1qm9a2	d1qmha1	d1qmhb1	d1qmia1	d1qmib1	d1qmic1
d1qmzb1	d1qmzd1	d1qn2b_	d1qn7b2	d1qnta1	d1qnua_	d1qnub_	d1qnuc_
d1qnue_	d1qpia2	d1qpwa_	d1qrj.1	d1qrnd2	d1qsed2	d1qsfd2	d1qspa_
d1qu1d_	d1qu7a_	d1quba2	d1quni2	d1qunk2	d1qunm2	d1quno2	d1qupa2
d1qvea_	d1qveb_	d1qvfw_	d1qvfx_	d1qvgp_	d1qvgw_	d1qvgx_	d1qvh.1
d1qvub2	d1qx2a_	d1qx2b_	d1qx7m_	d1qxfa_	d1qxpb1	d1qzea4	d1qzwa2
d1qzwe2	d1qzwg2	d1qzya1	d1r03a_	d1r0bj1	d1r0bk2	d1r0da_	d1r0db_
d1r0de_	d1r0df_	d1r0dg_	d1r0dh_	d1r0di_	d1r11a3	d1r11b3	d1r4ca_
d1r4cc_	d1r4cd_	d1r4ce_	d1r4cf_	d1r4cg_	d1r4ch_	d1r4mk_	d1r4ni_
d1r4qd_	d1r4qe_	d1r4qg_	d1r4qh_	d1r58a1	d1r5aa1	d1r5ga1	d1r5ha1
d1r5ie2	d1r5pa_	d1r5pb_	d1r5ra_	d1r5ui_	d1r5uk_	d1r6la2	d1r6ma2
d1r6od1	d1r6qc1	d1r6qd1	d1r7ja_	d1r83a_	d1r8ha_	d1r8hb_	d1r8hc_
d1r8he_	d1r8hf_	d1r8pa_	d1r8pb_	d1r9si_	d1r9sk_	d1r9tk_	d1rbli_
d1rblk_	d1rbll_	d1rblm_	d1rbln_	d1rblo_	d1rblp_	d1rboc_	d1rbof_
d1rbos_	d1rcba_	d1rcca_	d1rcda_	d1rcea_	d1rcga_	d1rcia_	d1rcoc_
d1rcoi_	d1rcom_	d1rcop_	d1rcos_	d1rcot_	d1rcow_	d1rcsa_	d1rcxc_
d1rcxi_	d1rcxm_	d1rcxp_	d1rcxs_	d1rcxt_	d1rcxw_	d1rd3.1	d1rewc_
d1rfaa_	d1rg0a_	d1rg0b_	d1rgbk1	d1rgea_	d1rgga_	d1rgha_	d1rgja_
d1rhya1	d1rhyb1	d1ri7a2	d1risa_	d1riya_	d1rjoa2	d1rk4a1	d1rk4b1
d1rkja1	d1rkja2	d1rkta2	d1rktb2	d1rl9a1	d1rlcs_	d1rlds_	d1rldt_
d1rlvb3	d1rm6b1	d1rm6c2	d1rm6e1	d1rm6f2	d1rmra_	d1rmva_	d1rn7a_
d1rqsa_	d1rqua2	d1rqub2	d1rqva2	d1rqvb2	d1rsci_	d1rscj_	d1rsck_
d1rscm_	d1rscn_	d1rsco_	d1rscp_	d1ruoa1	d1rvwa_	d1rw2a_	d1rw6a_
d1rxoc_	d1rxof_	d1rxoi_	d1rxos_	d1rxtd1	d1rxvb1	d1rxwa1	d1ry1b_
d1ry7b2	d1ryja_	d1ryqa_	d1ryra1	d1rysa1	d1rysb1	d1rzla_	d1s05a_
d1s0mb1	d1s0na1	d1s0oa1	d1s0ob1	d1s0ua2	d1s10a1	d1s1hj_	d1s1hl_
d1s1ig_	d1s1im_	d1s1qb_	d1s1qd_	d1s3ta_	d1s4ya_	d1s4zb_	d1s5yd_
d1s6oa_	d1s6ua_	d1s72x_	d1s72y_	d1s7oa_	d1s7ob_	d1s7oc_	d1s8ga_
d1s8ia_	d1s97a1	d1s97b1	d1s97c1	d1s97d1	d1s9fa1	d1s9fb1	d1s9fc1
d1s9va2	d1s9vd2	d1sapa_	d1sb3b1	d1sb3c2	d1sb3e1	d1sb3f2	d1sc6a3
d1sc6c3	d1sc6d3	d1scea_	d1scec_	d1scfa_	d1scfb_	d1scfc_	d1scfd_
d1sd0a1	d1sdda2	d1sdka_	d1sdkc_	d1sdla_	d1se9a_	d1seda_	d1sedb_
d1sega_	d1sf4a_	d1sf6a_	d1sfba_	d1sfea1	d1sfea2	d1sfga_	d1sfoi_
d1sfua_	d1sfub_	d1sg2c_	d1sg8.1	d1sgfy_	d1sgha1	d1sgha3	d1sgma2
d1sh5a2	d1sh5b2	d1sh6a2	d1si4a_	d1sifa_	d1siha2	d1siia2	d1siza_
d1sj1a_	d1sj1b_	d1sj8a1	d1sjea2	d1sjha2	d1sjpa3	d1sjpb3	d1skga_
d1skyb1	d1skyb2	d1skye2	d1slja_	d1smxb_	d1smya1	d1smyb1	d1smyk1
d1sn1a_	d1sn4a_	d1sn8b_	d1sofb1	d1soff1	d1spbp_	d1spga_	d1spua2

Continued on Next Page…

Table 1 – Continued

Domain Identifiers

d1spub2	d1spub3	d1sq3a1	d1sq3f1	d1sq3j1	d1sq3k1	d1sqga1	d1sqza_
d1ss6a_	d1ssfa2	d1ssoa_	d1st6a5	d1stza1	d1stzb1	d1stzc1	d1sv3a_
d1svdm1	d1sw8a_	d1sx4o1	d1sx4p1	d1sx4q1	d1sx4r1	d1sx4s1	d1sx4t1
d1sxka_	d1syma_	d1symb_	d1szha_	d1szhb_	d1t0ha_	d1t0ja_	d1t0qc_
d1t0ya_	d1t11a2	d1t11b2	d1t11b3	d1t14a1	d1t14b1	d1t15a2	d1t29a2
d1t2ua2	d1t2vb2	d1t2vc2	d1t37a_	d1t38a1	d1t39a1	d1t39b1	d1t39b2
d1t3la1	d1t3na1	d1t3qa2	d1t3qd2	d1t3sa1	d1t3wa_	d1t3wb_	d1t4lb_
d1t4oa_	d1t4ob_	d1t6sa1	d1t6sb1	d1t72b_	d1t72d_	d1t72e_	d1t72f_
d1t7ea_	d1t7sa_	d1t7sb_	d1t89c1	d1t8ba_	d1t8bb_	d1t94a1	d1t94b1
d1t98a2	d1t98b2	d1tbea_	d1tbeb_	d1tbpa1	d1tbpb1	d1tbxa_	d1tbxb_
d1tcra2	d1td7a_	d1tdha1	d1tdja3	d1tdpa_	d1tdva_	d1te4a_	d1teja_
d1tf2a2	d1tf5a1	d1tf5a2	d1tfva2	d1tg1a_	d1tg4a_	d1tgha2	d1tgma_
d1th6a_	d1th7a1	d1th7b1	d1th7c1	d1th7d1	d1th7e1	d1th7f1	d1th7g1
d1th7i1	d1th7j1	d1th7k1	d1th7l1	d1th7m1	d1th7n1	d1tifa_	d1tiga_
d1tijb1	d1tiua_	d1tiza_	d1tj1a1	d1tj2a1	d1tj9a_	d1tjfa1	d1tjfb1
d1tjqa_	d1tk4a_	d1tkva_	d1tkvb_	d1tl4a_	d1tl5a_	d1tl6a1	d1tlva1
d1tmt.1	d1tola1	d1tola2	d1tom.1	d1tp2a_	d1trfa_	d1troa_	d1troc_
d1trog_	d1trra_	d1trrb_	d1trrd_	d1trre_	d1trrg_	d1trrh_	d1trrj_
d1tttb2	d1tttc2	d1tu2a1	d1tu7a1	d1tu7a2	d1tu7b1	d1tu8a1	d1tu8b1
d1tu8d2	d1tuia2	d1tuib2	d1tuic2	d1tw9a1	d1tw9d1	d1tw9e1	d1tw9f1
d1twac1	d1twae2	d1twak_	d1twce2	d1twck_	d1twfe2	d1twfk_	d1twge2
d1twhe2	d1twhk_	d1twjc_	d1txya_	d1ty3b3	d1ty4a_	d1ty5b3	d1tygb_
d1tz0a_	d1tz0c_	d1tz1a1	d1tzua_	d1tzxa_	d1u1sa1	d1u1sb1	d1u1sc1
d1u1se1	d1u1sf1	d1u1ta1	d1u1tb1	d1u1tc1	d1u1td1	d1u1te1	d1u1tf1
d1u2vg_	d1u2wd1	d1u3em2	d1u42a_	d1u5tb1	d1u5tc2	d1u61a_	d1u6ga1
d1u6rb1	d1u73a_	d1u73b_	d1u8cb3	d1u8sa1	d1u8sa2	d1u8sb1	d1u8sb2
d1u98a2	d1u99a2	d1uawa_	d1ub4c_	d1ubca2	d1ubea2	d1ubia_	d1ubpa_
d1ucia_	d1ucja_	d1ucka_	d1ucla_	d1ucpa_	d1ucy.1	d1ud7a_	d1udva_
d1ue6a_	d1ue7c_	d1ueba1	d1uebb1	d1uela_	d1uewa_	d1uf0a_	d1uflb_
d1ufma_	d1ug1a_	d1uhfa_	d1uhma_	d1uhza_	d1ui5a2	d1ui5b2	d1ui6a2
d1ui7a2	d1ui7b2	d1ui8a2	d1ui8b2	d1uila_	d1ujoa_	d1ujua_	d1ujxa_
d1uk0a1	d1uk1a1	d1uk1b1	d1ul1y1	d1ul5a_	d1ul7a_	d1um2b2	d1um7a_
d1ungd_	d1unhd_	d1unhe_	d1unld_	d1unle_	d1unnc_	d1uotp1	d1uotp2
d1up5b1	d1up9a_	d1upda_	d1upgb_	d1upne1	d1upne2	d1urcd1	d1us6b_
d1usoa_	d1usob_	d1uv7a_	d1uv7b_	d1uvaa_	d1uvca_	d1uw9c1	d1uw9f1
d1uw9j1	d1uw9m1	d1uw9p1	d1uw9t1	d1uw9w1	d1uwac1	d1uwaf1	d1uwai1
d1uwam1	d1uwap1	d1uwat1	d1uwaw1	d1uwda_	d1ux8a_	d1uz5a1	d1uzja3
d1uzka3	d1uzpa3	d1uzqa3	d1uzxb_	d1v2aa1	d1v2ab1	d1v2ad1	d1v32a_
d1v3rb_	d1v3sb_	d1v3sc_	d1v40a1	d1v40b1	d1v40c1	d1v40d1	d1v4aa1
d1v5va1	d1v5vb1	d1v6ea_	d1v75a_	d1v80a1	d1v81a1	d1v97a2	d1v97b2
d1v9pa2	d1vaza_	d1vbva1	d1vcra_	d1vd0a1	d1vd4a_	d1vdva2	d1vdvb2
d1vg0a2	d1vhba_	d1vhbb_	d1vi7a2	d1viga_	d1viha_	d1vina1	d1vixa2
d1vjka_	d1vjqa_	d1vjqb_	d1vjwa_	d1vkaa_	d1vkab_	d1vkea_	d1vkee_
d1vkza1	d1vkzb1	d1vl9a_	d1vlta_	d1vm6a2	d1vm6b2	d1vm6c2	d1vm6d2
d1voqa_	d1voqf_	d1voqi_	d1voqm_	d1vosa_	d1vosf_	d1vosi_	d1vova_
d1vovi_	d1voxa_	d1voxf_	d1voxi_	d1voza_	d1vozf_	d1vozi_	d1vpda1
d1vq4x1	d1vq4y1	d1vq5q1	d1vq5x1	d1vq5y1	d1vq6x1	d1vq6y1	d1vq7x1

Continued on Next Page…

Table 1 – Continued

**Domain Identifiers**							
d1vq8q1	d1vq8x1	d1vq8y1	d1vq8z1	d1vq9q1	d1vq9x1	d1vqkq1	d1vqkx1
d1vqkz1	d1vqlq1	d1vqlx1	d1vqly1	d1vqmq1	d1vqmx1	d1vqnq1	d1vqnx1
d1vqoq1	d1vqox1	d1vqoy1	d1vqpq1	d1vqpx1	d1vqpy1	d1vqpz1	d1vqza1
d1vrpa1	d1vrpb1	d1vs6m1	d1vvca1	d1vvda1	d1vywb1	d1vywd1	d1vz0a2
d1vz0c2	d1vz0d2	d1vz0e2	d1vz0f2	d1vz0g2	d1vz0h2	d1w0ja1	d1w0ja2
d1w0jb2	d1w0jc2	d1w0jd1	d1w0jd2	d1w0je1	d1w0je2	d1w0jf1	d1w0jf2
d1w0ka2	d1w0kb1	d1w0kb2	d1w0kc2	d1w0kd1	d1w0kd2	d1w0ke1	d1w0ke2
d1w0kf2	d1w1we_	d1w1wf_	d1w1wg_	d1w1wh_	d1w26b2	d1w2bp_	d1w2bw_
d1w2za2	d1w2za3	d1w2zb2	d1w2zc2	d1w2zc3	d1w2zd2	d1w4na2	d1w4nb2
d1w5za2	d1w6ca2	d1w6ga2	d1w6va1	d1w7pc2	d1w96c1	d1wasa_	d1wb1c2
d1wb3c2	d1wcmk_	d1wd0a_	d1wd1a_	d1wdds_	d1wddw_	d1wdta3	d1wdta5
d1wdzb1	d1we3a3	d1we3b3	d1we3c3	d1we3d3	d1we3e3	d1we3f3	d1we3g3
d1we3i3	d1we3j3	d1we3k3	d1we3l3	d1we3m3	d1we3n3	d1we6a_	d1we8a_
d1weza_	d1wf4a3	d1wf4b3	d1wf4c3	d1wf4d3	d1wf4e3	d1wf4f3	d1wf4g3
d1wf4i3	d1wf4j3	d1wf4k3	d1wf4l3	d1wf4m3	d1wf4n3	d1wf4q_	d1wf4t_
d1wg5a_	d1wgga_	d1wgha_	d1wh2a_	d1wh4a_	d1wh8a_	d1wh9a_	d1whda_
d1whra_	d1whya_	d1whza_	d1wi5a_	d1wi9a_	d1wiaa_	d1wifa_	d1wija_
d1wipb2	d1wiqb2	d1wj4a_	d1wj5a_	d1wjja_	d1wjla_	d1wjna_	d1wjqa_
d1wk2a_	d1wm2a_	d1wm3a_	d1wmga_	d1wmgb_	d1wmgc_	d1wmgd_	d1wmge_
d1wmna2	d1wmnb2	d1wmoa2	d1wmob2	d1wmpa2	d1wmpb2	d1wn0c1	d1wn0d1
d1wokb1	d1wokc1	d1wooa1	d1wopa1	d1wora1	d1wosa1	d1woya1	d1wq2a1
d1wr1a1	d1wr6e1	d1wr6f1	d1wr6g1	d1wr6h1	d1wrdb1	d1wrpr_	d1wsud1
d1wtfb1	d1wtfc1	d1wtfd1	d1wtua_	d1wtub_	d1wtya_	d1wtyb_	d1wtyd_
d1wvla1	d1wvlb1	d1wvna1	d1wxma1	d1wxqa2	d1wxsa1	d1wy5b2	d1x04a1
d1x4ea1	d1x4fa1	d1x4ma1	d1x4na1	d1x5oa1	d1x5sa1	d1x5ua1	d1x6oa1
d1x8za_	d1x8zc_	d1x9fa_	d1x9fb_	d1x9fd_	d1x9fe_	d1x9ff_	d1x9fh_
d1x9fj_	d1x9fl_	d1xb4d1	d1xbwb_	d1xbwc_	d1xc2a1	d1xcba1	d1xcbb1
d1xcbd1	d1xcbe1	d1xd3b_	d1xd3d_	d1xdsb1	d1xeja_	d1xfna1	d1xfqa1
d1xfup1	d1xfuq1	d1xfur1	d1xfus1	d1xfut1	d1xfvp1	d1xfvs1	d1xfwo1
d1xfwq1	d1xfwr1	d1xfws1	d1xfwt1	d1xfyo1	d1xfyp1	d1xfyq1	d1xfyr1
d1xfyt1	d1xfzo1	d1xfzp1	d1xfzq1	d1xfzr1	d1xfzs1	d1xfzt1	d1xgsb1
d1xi8a2	d1xi8b2	d1xjva1	d1xjwb1	d1xjwb2	d1xk4g1	d1xk4h1	d1xkfa1
d1xkfb1	d1xkfb2	d1xmba2	d1xmol_	d1xmom_	d1xmqc1	d1xmqc2	d1xmql_
d1xmqq_	d1xmsa2	d1xmva2	d1xn8a_	d1xn9a_	d1xnia2	d1xnib2	d1xnic2
d1xnie2	d1xnif2	d1xnig2	d1xnih2	d1xnii2	d1xnij2	d1xnqc2	d1xnqm_
d1xnrc2	d1xnrj_	d1xnrm_	d1xo2a1	d1xoxa1	d1xoxb1	d1xp4c1	d1xp8a2
d1xpod2	d1xppa_	d1xppb_	d1xppc_	d1xppd_	d1xprb2	d1xprd2	d1xpre2
d1xpub2	d1xpuc2	d1xpud2	d1xpue2	d1xpxa1	d1xsva_	d1xsvb_	d1xsxa1
d1xt9b_	d1xtda1	d1xtda2	d1xwdd1	d1xwea_	d1xwga1	d1xx3a1	d1xxaa_
d1xxac_	d1xxad_	d1xxae_	d1xxaf_	d1xxbb_	d1xxbc_	d1xxbd_	d1xxbe_
d1xxcb_	d1xxcd_	d1xxce_	d1xxcf_	d1xxhb1	d1xxhc1	d1xxhd1	d1xxhe1
d1xxhh1	d1xxhi1	d1xxib1	d1xxic1	d1xxid1	d1xxie1	d1xxig1	d1xxih1
d1xxij1	d1xxwa1	d1xxwb1	d1xz1a1	d1xz3a1	d1y01b_	d1y0ta_	d1y0ua_
d1y14d2	d1y1vk_	d1y1wk_	d1y2ia_	d1y2ib_	d1y2ic_	d1y2id_	d1y2ie_
d1y2ob1	d1y2sa_	d1y2za_	d1y38a1	d1y4ra_	d1y5ha1	d1y5ha2	d1y5hb1
d1y77k_	d1y7pa2	d1y7pb2	d1y7pc2	d1y8ma1	d1y8rf1	d1y8wc_	d1y98a2
d1yava1	d1yava2	d1yavb1	d1yavb2	d1ybaa3	d1ybab3	d1ybac3	d1ybad3

Continued on Next Page…

**Domain Identifiers**							
d1ycya1	d1ycyb1	d1ycyc1	d1ycyd1	d1ydka1	d1ydkb1	d1yeaa_	d1yenc_
d1yeuc_	d1yevc_	d1yeza1	d1yfha1	d1yfhb1	d1yfhb2	d1yfhc1	d1yg5c_
d1ygoa1	d1ygya3	d1ygyb3	d1yh5a1	d1yh9c1	d1yhqx1	d1yhqy1	d1yhqz1
d1yi2x1	d1yi2y1	d1yi5f1	d1yi5g1	d1yi5h1	d1yi5i1	d1yi5j1	d1yijx1
d1yitx1	d1yity1	d1yiwa1	d1yiwb1	d1yiwc1	d1yj1a1	d1yj1b1	d1yj1c1
d1yj9q1	d1yj9x1	d1yj9y1	d1yj9z1	d1yjnq1	d1yjnx1	d1yjny1	d1yjqa1
d1yjwx1	d1yjwy1	d1yjwz1	d1ykbb1	d1ykbc1	d1ykbe1	d1yl3d1	d1yl3i2
d1yl3l2	d1yl3s1	d1yl3t1	d1yl4f1	d1yl4f2	d1yl4p1	d1yl4t1	d1yl6b2
d1ynjk1	d1ynnb1	d1yona1	d1yoza1	d1yozb1	d1ypna2	d1yqaa1	d1ysja2
d1yuhb2	d1yuhh2	d1yuua1	d1yv0c1	d1yvca1	d1yvha3	d1yvia1	d1yvib1
d1yw7a1	d1yw8a1	d1yw9a1	d1yx5b1	d1yx6b1	d1yyoa2	d1z00b1	d1z05a1
d1z2be1	d1z2ma2	d1z6oa1	d1z6ob1	d1z6oc1	d1z6od1	d1z6oe1	d1z6of1
d1z6oh1	d1z6oi1	d1z6oj1	d1z6ok1	d1z6ol1	d1z6om1	d1z6on1	d1z6oo1
d1z6oq1	d1z6or1	d1z6os1	d1z6ot1	d1z6ou1	d1z6ov1	d1z6ow1	d1z6ox1
d1z8la1	d1z8lb1	d1z8lc1	d1z8ld1	d1z8ma1	d1z8ub1	d1z8ud1	d1zeta1
d1zfsb1	d1zglh1	d1zglt2	d1zgna1	d1zgnb1	d1zgub1	d1zjka3	d1zk8b2
d1zl7a1	d1zlba1	d1zm2a4	d1zm2e4	d1zm3a4	d1zm4a4	d1zm6a1	d1zm9a4
d1zpvb1	d1zpvc1	d1zpwx1	d1zq1b1	d1zrcb1	d1zs3a1	d1zs3b1	d1zs3c1
d1zs3e1	d1zs3f1	d1zs3g1	d1zs3h1	d1zs3i1	d1zs3j1	d1zs3k1	d1zs3l1
d1zt9b1	d1zt9d1	d1zt9e1	d1zud21	d1zud41	d1zujc1	d1zujd1	d1zunb2
d1zxia2	d1zxid2	d1zyba1	d1zyrb2	d1zyrk1	d1zyxa1	d1zzfa1	d1zzfb1
d1zzib1	d1zzja1	d1zzjb1	d1zzjc1	d1zzna1	d20gsa1	d20gsb1	d21gsa1
d22gsa1	d22gsb1	d2a07f1	d2a07g1	d2a07h1	d2a07i1	d2a07j1	d2a07k1
d2a0fb1	d2a2ra1	d2a2rb1	d2a2sa1	d2a2sb1	d2a2ya1	d2a2yb1	d2a68a1
d2a68k1	d2a68l1	d2a69a1	d2a69b1	d2a69k1	d2a69l1	d2a6ab2	d2a6ea1
d2a6ek1	d2a6el1	d2a6ha1	d2a6hb1	d2a6hk1	d2a6hl1	d2a6qd1	d2a6ta1
d2a7ya1	d2a8va2	d2a8vb2	d2a8vc2	d2a9ua1	d2a9ub1	d2aawa1	d2aawc1
d2ad9a1	d2adca2	d2adua1	d2ae8a1	d2ae8b1	d2ae8c1	d2ae8d1	d2ae8e1
d2agoa1	d2agpa1	d2agpb1	d2agqa1	d2ahoa2	d2ahob1	d2ahob3	d2ahra1
d2ahrc1	d2ahrd1	d2ahre1	d2airb1	d2airh1	d2aita_	d2ajqi1	d2ak7a1
d2akwb1	d2akwb2	d2akwb5	d2alyb1	d2alyb2	d2amcb1	d2amcb2	d2amfa1
d2amfc1	d2amfd1	d2amfe1	d2ao9h1	d2aobd1	d2aopa1	d2arma1	d2arwa1
d2as5g1	d2asba1	d2asba3	d2asca1	d2asda1	d2asdb1	d2asja1	d2asjb1
d2aslb1	d2asqa1	d2assc1	d2asyb1	d2at1b2	d2at1d2	d2atla1	d2atlb1
d2atwa3	d2atwc1	d2atwc3	d2au0a1	d2au0b1	d2au5a1	d2aw0a_	d2aw2b1
d2aw4m1	d2awfa1	d2axya1	d2axyb1	d2axyc1	d2axyd1	d2ayba1	d2aybb1
d2ayeb1	d2ayec1	d2ayed1	d2ayee1	d2ayef1	d2ayob1	d2azya1	d2azza1
d2b01a1	d2b03a1	d2b04a1	d2b17a1	d2b26a1	d2b26b1	d2b2ta1	d2b2tb1
d2b2ua2	d2b2ub2	d2b2uc1	d2b2va2	d2b2vb1	d2b2vb2	d2b2vc1	d2b2wa2
d2b2wc1	d2b2ya2	d2b2yb2	d2b2yc1	d2b5ia1	d2b5ub1	d2b5ud1	d2b63k1
d2b64e2	d2b64q1	d2b6631	d2b66w1	d2b76n1	d2b78a1	d2b8kk1	d2b8ta2
d2b8td2	d2b9mc2	d2b9me2	d2b9mq1	d2b9n31	d2b9nw1	d2b9oc2	d2b9oe2
d2b9p31	d2b9pk2	d2b9pw1	d2bcaa_	d2bcba_	d2be5a1	d2be5b1	d2be5k1
d2bf8b1	d2bgfa1	d2bgfb1	d2bh2a1	d2bh2b1	d2bj1a2	d2bj1b2	d2bj3a2
d2bj3c2	d2bj3d2	d2bj7a2	d2bj7b2	d2bj8a2	d2bj8b2	d2bj9a2	d2bj9b2
d2bkya1	d2bkyb1	d2bkyx1	d2bkyy1	d2bkzb1	d2bkzd1	d2bm0a3	d2bm1a3
d2bpmb1	d2bpmd1	d2bppa_	d2bq3a1	d2bqra1	d2bqua1	d2br0a1	d2bt3a2

Continued on Next Page…

Table 1 – Continued

Domain Identifiers							
d2buna1	d2bv3a3	d2bwes1	d2bwet1	d2bweu1	d2bwfa1	d2bwfb1	d2bz2a1
d2c0ua1	d2c0ub1	d2c0uc1	d2c0ud1	d2c12a1	d2c12d1	d2c12f1	d2c22a1
d2c2ea1	d2c2ra1	d2c35a1	d2c35c1	d2c35e1	d2c35f2	d2c35g1	d2c35h2
d2c4gd1	d2c5ce1	d2c5cf1	d2c5ld1	d2c5od1	d2c5pb1	d2c5pd1	d2c5vb1
d2c5xb1	d2c5xd1	d2c62a1	d2c62b1	d2c6pa1	d2c6tb1	d2c6td1	d2c78a2
d2c7cp1	d2c7cq1	d2c7cr1	d2c7cs1	d2c7ct1	d2c7cu1	d2c7do1	d2c7dp1
d2c7dr1	d2c7ds1	d2c7dt1	d2c7du1	d2c7mb1	d2c7nb1	d2c7nd1	d2c7nf1
d2c7nj1	d2c7nl1	d2c80a1	d2c80b1	d2c8sa1	d2c8ua1	d2c8ub1	d2ca1b1
d2caia1	d2caia2	d2caib1	d2caqa1	d2cbla3	d2cchb1	d2cchd1	d2ccib1
d2cdqa2	d2cdqb2	d2ceia1	d2cfda2	d2cfdb2	d2cfga2	d2cfgb2	d2cfka2
d2cfwa2	d2cfxa2	d2cfxb2	d2cfxc2	d2cfxd2	d2cfxe2	d2cfxf2	d2cfxg2
d2cg0a2	d2cg1a2	d2cg4a2	d2cg4b2	d2cgto1	d2cgtp1	d2cgtq1	d2cgtr1
d2cgtt1	d2cgtu1	d2ch7a1	d2chsb_	d2chsc_	d2chsd_	d2chse_	d2chsf_
d2chsh_	d2chsi_	d2chsj_	d2chsk_	d2chsl_	d2chta_	d2chtb_	d2chtd_
d2chtg_	d2chti_	d2chtj_	d2chtk_	d2chtl_	d2cjmb1	d2cjna_	d2cjoa_
d2ck3d2	d2ck3e2	d2ck3f2	d2ckbl_	d2ckbm_	d2ckhb1	d2cmea1	d2cmeb1
d2cmee1	d2cmeg1	d2cnpa_	d2cnpb_	d2coma1	d2cp2a1	d2cpha1	d2cpja1
d2cpra1	d2cqaa1	d2cr5a1	d2crka1	d2crsa_	d2cs0a1	d2cs5a1	d2csha1
d2ctja1	d2ctka1	d2ctma1	d2cvda1	d2cvdb1	d2cvdc1	d2cvdd1	d2cvea2
d2cw0b1	d2cw0k1	d2cw0l1	d2cwta2	d2cwtb2	d2cwua2	d2cwub2	d2cwva2
d2cyka_	d2cyya2	d2cz4a1	d2cz4b1	d2cz4c1	d2d07b1	d2d10a1	d2d10a3
d2d10b3	d2d10c1	d2d10c3	d2d10d1	d2d10d3	d2d11a1	d2d11a3	d2d11b1
d2d11c1	d2d11c3	d2d11d1	d2d11d3	d2d1wa2	d2d1wb2	d2d2co1	d2d2qa1
d2d2qb1	d2d3ga1	d2d3gb1	d2d4zb1	d2d54a1	d2d5ba1	d2d6fa1	d2d6fb1
d2d6yb2	d2ddha1	d2denb1	d2dgxa1	d2disa1	d2djja1	d2dlna1	d2dmca1
d2dpdb1	d2dpza1	d2dt5a1	d2dt5b1	d2dv8a1	d2dx5b1	d2dy7a1	d2e1ea1
d2e1ra4	d2e2he2	d2e2hk1	d2e2ie2	d2e2ik1	d2e2jk1	d2e5lq1	d2efga3
d2eime1	d2eimr1	d2ekec1	d2eked1	d2erjd1	d2erjh1	d2esha1	d2esna1
d2etnb1	d2etnb2	d2eula2	d2eulb2	d2eulc2	d2euld2	d2evza2	d2exta1
d2extc1	d2ey4c1	d2ey4d1	d2eyqa1	d2eyya2	d2f06a2	d2f06b2	d2f1da1
d2f1dc1	d2f1dd1	d2f1de1	d2f1df1	d2f1dg1	d2f1dh1	d2f1di1	d2f1dj1
d2f1dl1	d2f1dm1	d2f1dn1	d2f1do1	d2f1dp1	d2f1fa1	d2f1fb1	d2f23a2
d2f43b2	d2f4vc2	d2f4vp1	d2f5ka1	d2f5kb1	d2f5kc1	d2f5kd1	d2f5ke1
d2f8fa1	d2f8fb1	d2faza1	d2fbqa2	d2fcea1	d2fcma1	d2fcmb1	d2fcna1
d2fcqa1	d2fcqb1	d2fcsa1	d2fcsb1	d2fd5a2	d2ffhb2	d2fhaa_	d2fj1a2
d2fl0a1	d2fl0e1	d2fnaa1	d2fnab1	d2fnxa1	d2foka3	d2fokb3	d2fq4a2
d2fzma1	d2fzna1	d2g1da1	d2g38b1	d2g38d1	d2g3pa1	d2g3pb1	d2g3qb1
d2g45e1	d2g4ha1	d2g58a1	d2g5cc1	d2g5cd1	d2g7ga2	d2g83b1	d2g9xb1
d2ga2a1	d2gaua1	d2gc4d1	d2gc4h1	d2gc4l1	d2gc4p1	d2gc7d1	d2gc7h1
d2gc7p1	d2gdma_	d2ge8a1	d2ge8b1	d2ge8i1	d2ge8j1	d2gf5a1	d2gf5a2
d2gfnb2	d2ggpa1	d2ggpb1	d2ghfa1	d2ghoa2	d2ghob1	d2gibb1	d2gjwa3
d2gjwc3	d2gjwd3	d2glra1	d2glrb1	d2gmhb2	d2gmib1	d2gmic1	d2gnka_
d2goob1	d2gooe1	d2gr7a1	d2gr7b1	d2gr7c1	d2gr7d1	d2gr7e1	d2gr7f1
d2gsrb1	d2gssa1	d2gssb1	d2gsta1	d2gtea1	d2gteb1	d2gtla1	d2gtlb1
d2gtld1	d2gtle1	d2gtlf1	d2gtlg1	d2gtlh1	d2gtli1	d2gtlj1	d2gtlk1
d2gvha2	d2gvhb2	d2gvhc2	d2gya52	d2gyda1	d2gzwd1	d2h46e1	d2h5fa1
d2h5ka1	d2h62c1	d2h64b1	d2h7za1	d2h7zb1	d2hbea_	d2hbfa_	d2hbja1

Continued on Next Page…

Table 1 – Continued

Domain Identifiers

d2hbla1	d2hbma1	d2hcbb1	d2hcbd1	d2hd5b1	d2hgif1	d2hgif2	d2hgip1
d2hgpf1	d2hgpf2	d2hgpp1	d2hgpt1	d2hgrf1	d2hgrf2	d2hgrm1	d2hgro1
d2hhea_	d2hhec_	d2hhhe1	d2hhhj1	d2hhhm1	d2hhhq1	d2hmfa3	d2hmfb3
d2hmfd3	d2hnt.1	d2hqia_	d2hqxa1	d2hqxb1	d2ht0a1	d2htha1	d2huwb1
d2hyih1	d2hymb1	d2hzaa2	d2hzab2	d2hzva2	d2hzvd2	d2hzve2	d2hzvg2
d2i09a2	d2i09b2	d2i2tr1	d2i2vr1	d2i40b1	d2i40d1	d2ibba1	d2ibga1
d2ibgd1	d2ibib1	d2iboc1	d2ibod1	d2ibzc1	d2imwp1	d2incc1	d2inta_
d2io1b1	d2io1d1	d2io1f1	d2isda1	d2iw8b1	d2iw8d1	d2iw9b1	d2iw9d1
d2iy5b1	d2iy5b2	d2iydb1	d2j00c2	d2j00f1	d2j00p1	d2j0111	d2j02c2
d2j02p1	d2j0qd1	d2j0qg1	d2j0sd1	d2j0wa2	d2j0xa2	d2j0xb2	d2j6sa1
d2j6ua1	d2j7qb1	d2j7qd1	d2j88l2	d2ja6g2	d2ja6k1	d2ja7w1	d2ja8k1
d2jdid2	d2jdie2	d2jdif2	d2jefa1	d2jega1	d2jeia1	d2jeja1	d2jn7a1
d2lh1a_	d2lh2a_	d2lh3a_	d2lh5a_	d2lh6a_	d2lh7a_	d2lynb_	d2mcg21
d2mpah2	d2mssa_	d2msta_	d2mtac_	d2mtal_	d2nefa_	d2nllb_	d2nota_
d2npfa4	d2npfb4	d2nqka1	d2nqkb1	d2nqma2	d2nqna1	d2nqnb1	d2nqqa1
d2nqqc1	d2nqqd1	d2nqra1	d2nqrb1	d2nqsa1	d2nqsb1	d2nqua1	d2nqub1
d2nqvb1	d2nr2a1	d2nroa1	d2nrob1	d2nrpb1	d2nrsa1	d2nrsb1	d2nsua1
d2nvqe2	d2nvqk1	d2nvte2	d2nvtk1	d2nvui1	d2nvxe2	d2nvxk1	d2nvye2
d2nvzc1	d2nvze2	d2nvzk1	d2o16b1	d2o1na1	d2o1pa1	d2o39c2	d2o39d2
d2o6vc1	d2o6ve1	d2o6vg1	d2o7oa2	d2o97a1	d2oa7a1	d2oa7b1	d2oaca1
d2oada1	d2oadb1	d2oara1	d2oarb1	d2oarc1	d2oare1	d2oaza1	d2occe_
d2odka1	d2odkb1	d2odkc1	d2odkd1	d2ofpa1	d2ofpb1	d2olia1	d2omva1
d2oobb1	d2oova2	d2oovb2	d2oovc2	d2oovd2	d2oove2	d2oovf2	d2opzd1
d2oqeb2	d2oqec2	d2oqed2	d2oqee2	d2oqef2	d2otfa1	d2otha1	d2otjx1
d2otlx1	d2otly1	d2otlz1	d2ouba1	d2ow8f2	d2ow8i1	d2ow8j1	d2oyfa1
d2p8wt4	d2p8wt5	d2p8xt4	d2p8xt5	d2p8yt4	d2p8yt5	d2p8zt4	d2p8zt5
d2p9cb3	d2p9ea3	d2p9eb3	d2p9ec3	d2p9ed3	d2p9ga3	d2p9gb3	d2p9ng1
d2p9ug1	d2pa3a3	d2pala_	d2pasa_	d2pawa1	d2paxa1	d2pb8a1	d2pc6a2
d2pc6c2	d2pc6d1	d2pc6d2	d2pe6b1	d2pg1a1	d2pg1b1	d2pg1c1	d2pg1d1
d2pghc_	d2pgta1	d2pgtb1	d2phma1	d2pjfa1	d2pmja1	d2pmta1	d2pmtb1
d2pmtd1	d2pqua1	d2pqub1	d2puc1	d2pqud1	d2prob2	d2pvba_	d2pwsa1
d2py9b1	d2py9c1	d2py9d1	d2pyca1	d2q1pa1	d2q43a2	d2q4fa1	d2q4fb1
d2sxla_	d2tcta2	d2trta2	d2u1aa_	d2u2fa_	d2ubpa_	d2uu9c2	d2uu9l1
d2uu9q1	d2uuac2	d2uuam1	d2uuaq1	d2uubc2	d2uubl1	d2uubm1	d2uubq1
d2uucj1	d2uucm1	d2uucq1	d2uueb1	d2uued1	d2uvra1	d2uvua1	d2uvva1
d2uwma1	d2uwmb1	d2uxcc2	d2uxcf1	d2uxcj1	d2uxcm1	d2uxcq1	d2uz3a2
d2uz3c2	d2uz3d2	d2vhba_	d2vhbb_	d2visb2	d2ygsa_	d2ypna2	d2yu9e2
d2yvca1	d2yvca3	d2yvcb1	d2yvcb3	d2yvcc1	d2yvcc3	d3aita_	d3aopa1
d3bp2a_	d3crda_	d3ctna_	d3cyra_	d3eipb_	d3fyga1	d3fygb1	d3geoa1
d3gssb1	d3gsta1	d3gstb1	d3gtub1	d3gtud1	d3hsfa_	d3inkd_	d3lynb_
d3mefa_	d3orca_	d3pala_	d3paxa1	d3pgta1	d3pgtb1	d3prod1	d3rubs_
d3sxla2	d3sxlb2	d3sxlc2	d3ubpa_	d3vhba_	d3vhbb_	d3wrpa_	d3ygsc_
d4bp2a_	d4cpva_	d4gssa1	d4gssb1	d4gtuf1	d4gtuh1	d4htci_	d4pala_
d4pgta1	d4pgtb1	d4prod1	d4rubs_	d4rubt_	d4rubu_	d4rubv_	d4ubpa_
d4vhbb_	d5aopa1	d5croc_	d5gepa1	d5gssa1	d5gssb1	d5gstb1	d6croa_
d6gssa1	d6gssb1	d6gsta1	d6gstb1	d6gsua1	d6gsub1	d6gswa1	d6gsya1
d7at1b2	d7ceib_	d7gssa1	d7gssb1	d7kme.1	d8at1b2	d8at1d2	d8atcb2

Continued on Next Page...

Table 1 – Continued

Domain Identifiers							
d8gssa1	d8gssb1	d8gssc1	d8ruci_	d8rucj_	d8ruck_	d8rucl_	d9gssa1
