# Supplementary material for: Automatic structure classification of small proteins using random forest
Source: BMC Bioinformatics. 2010 Jul 1;11:364. doi: 10.1186/1471-2105-11-364 (PMC2916923; doi:10.1186/1471-2105-11-364)
Supplement: Additional file 8 — Domains consisting of 6SSEs from SCOP version 1.69. This file lists the identifiers for the 6SSEs containing domains from SCOP version 1.69. [file 1471-2105-11-364-S8.PDF]

Additional File 8

Domains consisting of 6SSEs from SCOP version 1.69

Table 1: Domains consisting of 6SSEs from SCOP version 1.69

Domain Identifiers							
d1914a1	d1a1za_	d1a2c.1	d1a2sa_	d1a3b.1	d1a43a_	d1a4pa_	d1a4w.1
d1a5ka_	d1a5oa_	d1a5ra_	d1a61.1	d1a7va_	d1a7vb_	d1a8oa_	d1aa2a_
d1abj.1	d1aboa_	d1abob_	d1abqa_	d1abta_	d1acbi_	d1acia_	d1acmd2
d1ad5b1	d1adra_	d1adta3	d1adua3	d1adub3	d1adva3	d1advb3	d1aela_
d1aeya_	d1afha_	d1ag2a_	d1aisb1	d1aisb2	d1aiwa_	d1akka_	d1alua_
d1amya1	d1aofa1	d1aoic_	d1aoig_	d1aoka_	d1aokb_	d1aopa1	d1aoqa1
d1ap4a_	d1aqda2	d1aqdd2	d1aqdg2	d1aqdj2	d1auaa1	d1auma_	d1avaa1
d1avza_	d1avzb_	d1avzc_	d1awoa_	d1ax8a_	d1azea_	d1azgb_	d1b07a_
d1b0xa_	d1b0ya_	d1b10a_	d1b1ba1	d1b33n_	d1b33o_	d1b41b_	d1b43a1
d1b47a2	d1b47b2	d1b47c2	d1b4fa_	d1b4fb_	d1b4fc_	d1b4fd_	d1b4fe_
d1b4fg_	d1b4fh_	d1b4pa1	d1b5g.1	d1b68a_	d1b6ra1	d1b6sa1	d1b6sb1
d1b6sd1	d1b71a1	d1b7ka_	d1b7va_	d1b9ma4	d1ba8.1	d1baja_	d1bbhb_
d1bbza_	d1bbzc_	d1bbze_	d1bbzg_	d1bccc2	d1bcfa_	d1bcfb_	d1bcfc_
d1bcfe_	d1bcff_	d1bcfg_	d1bcfh_	d1bcfi_	d1bcfj_	d1bcfk_	d1bcfl_
d1be3c2	d1be3d2	d1beyl2	d1bfra_	d1bfrb_	d1bfrc_	d1bfrd_	d1bfre_
d1bfrg_	d1bfrh_	d1bfri_	d1bfrj_	d1bfrk_	d1bfrl_	d1bfrm_	d1bfrn_
d1bfrp_	d1bfrq_	d1bfrr_	d1bfrs_	d1bfrt_	d1bfru_	d1bfrv_	d1bfrw_
d1bg1a1	d1bg5a1	d1bg7a_	d1bg9a1	d1bgda_	d1bgea_	d1bgeb_	d1bgxt2
d1bi1a1	d1bi2a1	d1bi2b1	d1bi3a1	d1bi3b1	d1biaa1	d1biaa2	d1biba1
d1bjpc_	d1bjpd_	d1bk2a_	d1bkra_	d1bl9b1	d1blba1	d1blbb2	d1blbd1
d1blqa_	d1bmoa3	d1bmob3	d1bmta1	d1bmtb1	d1bo9a_	d1bpda1	d1bpea2
d1bpya1	d1bpza1	d1bpza3	d1bqva_	d1bqza_	d1br1a1	d1br1c1	d1br1e1
d1br2a1	d1br2b1	d1br2c1	d1br2d1	d1br2e1	d1br2f1	d1bt6b_	d1bu1a_
d1bu1c_	d1bu1d_	d1bu1e_	d1bu1f_	d1bu2a2	d1buya_	d1bvsa2	d1bvsb2
d1bvsd2	d1bvse2	d1bvsf2	d1bvsg2	d1bvsh2	d1bwob_	d1bx2d2	d1bxya_
d1bz4a_	d1c0ma1	d1c0mb1	d1c0mc1	d1c0md1	d1c0wa1	d1c0wb1	d1c0wc1
d1c1aa1	d1c1ab1	d1c1za5	d1c2aa1	d1c2na_	d1c3za_	d1c4v.1	d1c5mf_
d1c6ob_	d1c6ra_	d1c6vx_	d1c72a1	d1c72b1	d1c72c1	d1c72d1	d1c7ma_
d1c9be1	d1c9bi1	d1c9bm1	d1c9bq1	d1c9fa_	d1c9oa_	d1c9ob_	d1cb9a_
d1ccha_	d1ccqa_	d1ccra_	d1cdba_	d1cdta_	d1cdtb_	d1ceda_	d1cf7b_
d1cfpb_	d1cfwa_	d1cfwb_	d1cgme_	d1chha_	d1chia_	d1chja_	d1ci4a_
d1ciea_	d1cifa_	d1ciga_	d1ciha_	d1ck7a3	d1ck7a4	d1ck7a5	d1ckaa_
d1ckla1	d1cklb1	d1cklc1	d1ckld1	d1ckle1	d1cklf1	d1ckua_	d1ckub_
d1cmca_	d1cnoa_	d1cnob_	d1cnoc_	d1cnod_	d1cnoe_	d1cnof_	d1cnog_
d1co0b_	d1co6a_	d1coda_	d1coea_	d1cooa_	d1cora_	d1coua_	d1cowf1
d1cpqa_	d1crca_	d1crfa_	d1crga_	d1crha_	d1cria_	d1crja_	d1crka1
d1crxa1	d1crxb1	d1crya_	d1csei_	d1cska_	d1cskb_	d1cskc_	d1cskd_
d1csua_	d1csva_	d1cswa_	d1csxa_	d1ctja_	d1ctla2	d1ctya_	d1ctza_
d1cvjh2	d1cvoa_	d1cvwl_	d1cxna_	d1cxoa_	d1cxwa_	d1cyia_	d1cyja_
d1d0ya1	d1d2ka2	d1d3bb_	d1d3bf_	d1d3bh_	d1d3ub2	d1d5na1	d1d5nb1
d1d5nd1	d1d6ea2	d1d6ri_	d1d8ba_	d1d8la1	d1d8la2	d1d8lb1	d1d9na_
d1dcda_	d1dcdb_	d1ddna1	d1ddnb1	d1ddnc1	d1ddnd1	d1dfxa2	d1dhga_

Continued on Next Page...

Table 1 – Continued

**Domain Identifiers**							
d1di2a_	d1di2b_	d1diva2	d1dj7a_	d1dksa_	d1dkxa1	d1dkza1	d1dlha2
d1dova_	d1dpra1	d1dprb2	d1dpsa_	d1dpsb_	d1dpsc_	d1dpsd_	d1dpse_
d1dpsg_	d1dpsh_	d1dpsi_	d1dpsj_	d1dpsk_	d1dpsl_	d1dpya_	d1dqca_
d1drsa_	d1dtjb_	d1duga1	d1dugb1	d1dula_	d1dura_	d1dvba1	d1dvha_
d1dvva_	d1dw9a1	d1dw9b1	d1dw9c1	d1dw9d1	d1dw9e1	d1dw9f1	d1dw9g1
d1dw9i1	d1dw9j1	d1dwka1	d1dwkb1	d1dwkc1	d1dwkd1	d1dwke1	d1dwkf1
d1dwkh1	d1dwki1	d1dwkj1	d1dwya_	d1dwza_	d1dx0a_	d1dx1a_	d1dx5i2
d1dx5k2	d1dx5l2	d1dxga_	d1dxgb_	d1dxsa_	d1dxxa1	d1dxxb1	d1dxxc1
d1dy7b1	d1dz1a_	d1dz1b_	d1e0ba_	d1e0bb_	d1e0fj_	d1e0fk_	d1e1ga_
d1e1qe1	d1e1qf1	d1e1rd1	d1e1rf1	d1e1sa_	d1e1ua_	d1e2ra1	d1e5wa1
d1e6ga_	d1e6ha_	d1e6ia_	d1e7db2	d1e7la2	d1e7lb2	d1e7oa_	d1e88a1
d1e8ba1	d1e8ba3	d1e8ea_	d1e8la_	d1e8ob_	d1e8oc_	d1e8od_	d1e8ra_
d1eaka5	d1eakb3	d1eakb4	d1eakb5	d1eakc4	d1eboc_	d1eeja2	d1eejb2
d1ef1c_	d1ef1d_	d1ef5a_	d1efna_	d1efnb_	d1efnc_	d1efnd_	d1eg3a2
d1egca1	d1egcd1	d1eh6a2	d1eh7a2	d1eh8a2	d1ehja_	d1eifa2	d1eika_
d1eiyb2	d1eiyb4	d1ej5a_	d1ekla_	d1ekza_	d1em7a_	d1emra_	d1en4a1
d1en4d1	d1en5b1	d1en5c1	d1en5d1	d1en6a1	d1en6b1	d1en6c1	d1en7a2
d1enma1	d1enwa_	d1eqza_	d1eqze_	d1eraa_	d1erga_	d1es0a2	d1etpa2
d1euma_	d1eumb_	d1eumc_	d1eumd_	d1eume_	d1eumf_	d1ex4a1	d1ex4b1
d1eyta_	d1ezaa1	d1ezba1	d1ezca1	d1ezda1	d1ezqb_	d1ezvf_	d1f02t_
d1f0ya1	d1f0yb1	d1f12a1	d1f12b1	d1f14a1	d1f14b1	d1f17a1	d1f17b1
d1f22a_	d1f2ri_	d1f30a_	d1f30b_	d1f30c_	d1f30d_	d1f30e_	d1f30f_
d1f30h_	d1f30i_	d1f30j_	d1f30k_	d1f30l_	d1f33a_	d1f33b_	d1f33c_
d1f33e_	d1f33f_	d1f33g_	d1f33h_	d1f33i_	d1f33j_	d1f33k_	d1f33l_
d1f3mb_	d1f44a1	d1f45b_	d1f5ta1	d1f5tb1	d1f5tc1	d1f66c_	d1f66g_
d1f80d_	d1f80e_	d1f81a_	d1f8ub_	d1f9rd_	d1fada_	d1fakl2	d1fapb_
d1faza_	d1fbva2	d1fc3b_	d1fcaa_	d1fccc_	d1fccd_	d1fcda3	d1fcdc2
d1fd4a_	d1fd4e_	d1fd4g_	d1fd4i_	d1fd4m_	d1fd4n_	d1fe5a_	d1ff4a_
d1fhua2	d1fi3a_	d1fi7a_	d1fj0a_	d1fj0b_	d1fj0c_	d1fj0d_	d1fk0a_
d1fk2a_	d1fk3a_	d1fk4a_	d1fk5a_	d1fk6a_	d1fk7a_	d1flcb_	d1flcd_
d1flia_	d1fmka1	d1fn4b2	d1fnea2	d1fnec2	d1fnga2	d1fngc2	d1foxa_
d1fqvb1	d1fqvd1	d1fqvf1	d1fqvh1	d1fqvj1	d1fqvl1	d1fqvn1	d1fqvp1
d1fr3a_	d1fr3b_	d1fr3c_	d1fr3d_	d1fr3e_	d1fr3f_	d1fr3g_	d1fr3h_
d1fr3j_	d1fr3k_	d1fr3l_	d1fs1b2	d1fv1a2	d1fv1d2	d1fw1a1	d1fwza1
d1fx7b1	d1fx7c1	d1fx7d1	d1fyna_	d1g0ta2	d1g0tb2	d1g0za_	d1g0zb_
d1g2913	d1g2923	d1g2ba_	d1g2xa_	d1g2xc_	d1g33a_	d1g3nc2	d1g3ng2
d1g3ta1	d1g3tb1	d1g3wa1	d1g3ya1	d1g4da_	d1g6ma_	d1g6ra2	d1g7da_
d1g83a1	d1g83b1	d1g8eb_	d1g8qa_	d1g8qb_	d1g9la_	d1gbqa_	d1gcpa_
d1gcpc_	d1gcpd_	d1gcqa_	d1gcqb_	d1gcqc_	d1gcya1	d1gdva_	d1gh7a4
d1gh9a_	d1ghqb2	d1ghua_	d1giwa_	d1gkna1	d1gksa_	d1gl4a2	d1gl5a_
d1gm5a1	d1gnca_	d1gnea1	d1go3e2	d1go3m2	d1gqaa_	d1gqad_	d1gria1
d1grib1	d1grib2	d1grja1	d1gs9a_	d1gsua1	d1gsub1	d1gsyb1	d1gtda_
d1gtua1	d1gtub1	d1gtuc1	d1gtud1	d1guga_	d1gugb_	d1gugc_	d1gugd_
d1gugf_	d1guna_	d1gunb_	d1gunc_	d1gund_	d1gune_	d1gunf_	d1guoa_
d1guoc_	d1guod_	d1guoe_	d1guof_	d1gusa_	d1gusb_	d1gusc_	d1gusd_
d1gusf_	d1guta_	d1gutb_	d1gutc_	d1gutd_	d1gute_	d1gutf_	d1guwa_
d1gxda5	d1gxda6	d1gxdb4	d1gxdb5	d1gxdb6	d1gxie_	d1gyfa_	d1gyoa_

Continued on Next Page…

Table 1 – Continued

**Domain Identifiers**							
d1h0ja_	d1h0jb_	d1h0jc_	d1h0la_	d1h1oa1	d1h1oa2	d1h1ob2	d1h2qp1
d1h33b_	d1h34a_	d1h3da2	d1h4sb3	d1h6ga1	d1h6ga2	d1h6gb2	d1h7ia_
d1h8ee1	d1h8hd1	d1h8he1	d1h8ka_	d1h8pa2	d1h8pb2	d1h92a_	d1h95a_
d1h99a2	d1h9ja1	d1h9ja2	d1h9ka1	d1h9ka2	d1h9ma1	d1h9ma2	d1h9mb1
d1h9xa1	d1h9xb1	d1h9ya1	d1h9yb1	d1haaa_	d1haja_	d1hap.1	d1hbxb_
d1hbxe_	d1hc8a_	d1hc8b_	d1hcma1	d1hcmb1	d1hd3a_	d1hdma2	d1he8a1
d1hekb_	d1heze_	d1hfel2	d1hfem2	d1hh2p1	d1hh7a_	d1hhna_	d1hipa_
d1hiwb_	d1hiwc_	d1hiwq_	d1hiwr_	d1hiws_	d1hj3a1	d1hj3b1	d1hj4a1
d1hjma_	d1hjna_	d1hk0x1	d1hkja2	d1hksa_	d1hkta_	d1hnba1	d1hnxm_
d1hq1a_	d1hq3a_	d1hq3e_	d1hqra2	d1hrca_	d1hroa_	d1hrob_	d1hrqa_
d1ht6a1	d1ht9a_	d1ht9b_	d1htmb_	d1htmd_	d1htmf_	d1hueb_	d1hula_
d1huoa1	d1huob1	d1huza1	d1huza3	d1huzb1	d1huzb3	d1hxda2	d1hxdb2
d1hxia_	d1hxya2	d1hyia_	d1hyja_	d1hz5a_	d1hz5b_	d1hz9a_	d1hz9b_
d1hzab_	d1hzba_	d1hzbb_	d1hzca_	d1hzcb_	d1hzva1	d1i02a_	d1i08b1
d1i08d1	d1i0ca_	d1i0cb_	d1i0hb1	d1i1rb_	d1i36a1	d1i36b1	d1i3ra2
d1i3re2	d1i3rg2	d1i49b_	d1i4db_	d1i54a_	d1i54b_	d1i55a_	d1i55b_
d1i5fb_	d1i5na_	d1i5nb_	d1i5nc_	d1i5nd_	d1i5ob2	d1i5od2	d1i5ta_
d1i6ea_	d1i6fa_	d1i6ga_	d1i8oa_	d1i8pa_	d1i8pb_	d1i8pc_	d1i8pd_
d1i8tb2	d1ib8a2	d1ic8b2	d1id3g_	d1ieaa2	d1ieac2	d1ieba2	d1iebc2
d1iesf_	d1ig4a_	d1igca_	d1igda_	d1igna1	d1igna2	d1ignb1	d1ignb2
d1igqb_	d1igqc_	d1igqd_	d1igua_	d1igub_	d1igyb3	d1igyd3	d1ihva_
d1ihwa_	d1ihwb_	d1il0a1	d1il0b1	d1imoa_	d1io3a_	d1ioel_	d1ioxa_
d1iq9a_	d1iqba1	d1iqbb1	d1iqel_	d1iqgl_	d1iqil_	d1iqll_	d1iqml_
d1iqpb1	d1iqpc1	d1iqpd1	d1iqpe1	d1iqpf1	d1irja_	d1irjc_	d1irjh_
d1irwa_	d1is2b1	d1it4a_	d1it5a_	d1iuaa_	d1iv5a_	d1ix9a1	d1ix9b1
d1ixbb1	d1ixra1	d1ixrb2	d1iy4a_	d1j26a_	d1j30a_	d1j30b_	d1j3sa_
d1j59b1	d1j5ya1	d1j7ma_	d1j8ba_	d1j8hd2	d1j9cl2	d1j9ia_	d1j9oa_
d1ja4a_	d1ja6a_	d1jafa_	d1jafb_	d1jb0e_	d1jbul_	d1jdaa1	d1jdca1
d1jdla_	d1je9a_	d1jega_	d1jexa_	d1jfib_	d1jgca_	d1jgcb_	d1jgcc_
d1jhfa1	d1jhha1	d1ji5a_	d1ji5b_	d1ji5c_	d1ji5d_	d1jida_	d1jiga_
d1jigc_	d1jigd_	d1jj2a2	d1jj2p_	d1jj2y_	d1jjua2	d1jlia_	d1jlta_
d1jmwa_	d1jmxa1	d1jmxa2	d1jmza1	d1jmza2	d1jo8a_	d1joca1	d1jocb1
d1jou.3	d1jowa2	d1jqia1	d1jqqa_	d1jqqb_	d1jqqc_	d1jqqd_	d1jr1a3
d1jr8b_	d1jr9a1	d1jraa_	d1jrac_	d1jrad_	d1jrea_	d1jreb_	d1jrec_
d1jree_	d1jref_	d1jreg_	d1jreh_	d1jrei_	d1jrej_	d1jrek_	d1jrel_
d1jroa1	d1jroc1	d1jroe1	d1jrog1	d1jrpa1	d1jrpc1	d1jrpe1	d1jrpg1
d1jrxa1	d1jrxb1	d1jrya1	d1jryb1	d1js2a_	d1js2b_	d1js2c_	d1js2d_
d1jt0b2	d1jt0c2	d1jt6a2	d1jt6b2	d1jt6d2	d1jt6e2	d1jtba_	d1jtsa_
d1jtsc_	d1jtsd_	d1jtse_	d1jtsf_	d1jtsg_	d1jtsh_	d1jtsi_	d1jtsj_
d1jtsl_	d1jtsm_	d1jtsn_	d1jtso_	d1jtsp_	d1jtsq_	d1jtsr_	d1jtss_
d1jtsu_	d1jtsv_	d1jtsw_	d1jtsx_	d1jtxa2	d1jtxd2	d1jtxe2	d1jtya2
d1jtyd2	d1jtye2	d1ju5c_	d1juma2	d1jumd2	d1jume2	d1jupa2	d1jupd2
d1jusa2	d1jusd2	d1juse2	d1jwma2	d1jwsa2	d1jyba1	d1jzdb2	d1jzoa2
d1k04a_	d1k05a_	d1k05b_	d1k05c_	d1k0ha_	d1k1za_	d1k25d2	d1k28a2
d1k2hb_	d1k3ga_	d1k3ha_	d1k3ra1	d1k3rb1	d1k40a_	d1k50c_	d1k52a_
d1k53a_	d1k53b_	d1k5oa_	d1k6oc_	d1k731_	d1k73c2	d1k73r_	d1k76a_
d1k8a1_	d1k8ac2	d1k8wa3	d1k96a_	d1k9aa1	d1k9ab1	d1k9ac1	d1k9ad1

Continued on Next Page...

Table 1 – Continued

Domain Identifiers

d1k9af1	d1k9m1_	d1k9mc2	d1k9ua_	d1k9ub_	d1kb0a1	d1kb2a_	d1kb2b_
d1kb6a_	d1kb6b_	d1kb9g_	d1kbab_	d1kbua1	d1kbub1	d1kc81_	d1kc8c2
d1kd11_	d1kd1c2	d1kdha3	d1kf6a1	d1kf6c_	d1kf6d_	d1kf6m1	d1kf6p_
d1kfyc_	d1kfyd_	d1kfym1	d1kfyp_	d1kfza_	d1kg1a_	d1kh0a_	d1kh0b_
d1kibb_	d1kibc_	d1kibd_	d1kibe_	d1kibf_	d1kibg_	d1kibh_	d1kika_
d1kkxa_	d1kl8a_	d1kl9a1	d1kl9a2	d1klaa_	d1klab_	d1klil_	d1kljl_
d1kn5a_	d1kp4a_	d1kq8a_	d1kqsa2	d1kqsy_	d1kqva_	d1kr7a_	d1krca_
d1kswa1	d1kt2a2	d1kt2c2	d1ktda2	d1ktdc2	d1ktma_	d1kts.1	d1ku6b_
d1kv9a1	d1kvva_	d1kw4a_	d1kwia_	d1kx3c_	d1kx3g_	d1kx4c_	d1kx4g_
d1kx5g_	d1kxia_	d1kxib_	d1ky9b2	d1kyow_	d1l0lc1	d1l0nc1	d1l0va1
d1l0vd_	d1l0vp_	d1l1pa_	d1l2na_	d1l3pa_	d1l4wa_	d1l6ja3	d1l6ja4
d1l6ki_	d1l6l5_	d1l6l6_	d1l6lb_	d1l6lq_	d1l6ly_	d1l7ca1	d1l7ca2
d1l7cc1	d1l7cc2	d1l8ha_	d1l8hb_	d1l8hc_	d1l8hd_	d1l8he_	d1l8hf_
d1l8hh_	d1l8hi_	d1l8hj_	d1l8hk_	d1l8hl_	d1l8ia_	d1l8ib_	d1l8ic_
d1l8ie_	d1l8if_	d1l8ig_	d1l8ih_	d1l8ii_	d1l8ij_	d1l8ik_	d1l8il_
d1lc2a_	d1ld9a1	d1ld9d1	d1ldkb1	d1le2a_	d1le4a_	d1le6a_	d1le6b_
d1le7a_	d1le7b_	d1leaa_	d1leba_	d1lfma_	d1lfmb_	d1lhd.1	d1lhe.1
d1li5a1	d1li5b1	d1li7a1	d1li7b1	d1liha_	d1lipa_	d1lisa_	d1lkma1
d1lkpa1	d1ll4a2	d1ll4b2	d1ll4c2	d1ll4d2	d1ll6a2	d1ll6b2	d1ll6c2
d1ll7a2	d1ll7b2	d1llia_	d1llib_	d1lmb3_	d1lmb4_	d1lnga_	d1lnqa2
d1lnqf2	d1lo5a2	d1lp9l2	d1lpaa2	d1lpea_	d1lpqa1	d1ls4a_	d1ls9a_
d1lsjb1	d1lsoa1	d1lsob1	d1lvaa3	d1lvaa4	d1lwba_	d1lxfc_	d1lyna_
d1m18c_	d1m18g_	d1m19c_	d1m19g_	d1m1ag_	d1m1ga2	d1m1gb2	d1m1gc2
d1m1k1_	d1m1kc2	d1m1xb3	d1m27c_	d1m2oa1	d1m2oc5	d1m2va5	d1m2vb1
d1m3aa_	d1m3ba_	d1m3ca_	d1m60a_	d1m6ta_	d1m6za2	d1m6zb2	d1m6zc2
d1m70a2	d1m70b2	d1m70c2	d1m70d2	d1m75a1	d1m75b1	d1m76a1	d1m76b1
d1m90c2	d1m99a1	d1m9aa1	d1m9ba1	d1ma7a1	d1ma7b1	d1maba2	d1mahf_
d1mc8a1	d1mdaa_	d1mdvb_	d1meei_	d1mfwa_	d1mg4a_	d1mg8a_	d1mgta2
d1mhhe_	d1mhhf_	d1mhxa_	d1mi0a_	d1mi0b_	d1mida_	d1mixa1	d1mj2a_
d1mjea1	d1mjka_	d1mjla_	d1mjma_	d1mjpa_	d1mjpb_	d1mk9d1	d1mkya3
d1mmmb1	d1mmsa1	d1mmsa2	d1mmsb_	d1mn8a_	d1mn8b_	d1mn8c_	d1mn8d_
d1mnmb_	d1mnmc_	d1mnmd_	d1moja_	d1mojb_	d1mojc_	d1mojd_	d1moxc_
d1mq2a1	d1mq2a2	d1mq3a1	d1mqvb_	d1msza_	d1mtca1	d1mtcb1	d1mvfd_
d1mwza_	d1mxaa2	d1mxba2	d1mxca2	d1mxlc_	d1mzla_	d1mzma_	d1n15b1
d1n1qb_	d1n1qc_	d1n1qd_	d1n29a_	d1n34e2	d1n34j_	d1n36l_	d1n36p_
d1n5ha_	d1n5pa_	d1n5za_	d1n5zb_	d1n6ja_	d1n6jb_	d1n72a_	d1n89a_
d1n8rc2	d1n9ca_	d1n9wa1	d1n9wb1	d1na3a_	d1na3b_	d1nafa_	d1nb5l_
d1nbeb2	d1nbed2	d1nbmd1	d1nd9a_	d1neaa_	d1neba_	d1nega_	d1neha_
d1nera_	d1nexa1	d1nexc1	d1nf4a_	d1nf4d_	d1nf4e_	d1nf4f_	d1nf4g_
d1nf4l_	d1nf4m_	d1nf4n_	d1nf4o_	d1nf4p_	d1nf6a_	d1nf6b_	d1nf6c_
d1nf6h_	d1nf6j_	d1nf6l_	d1nf6m_	d1nf6n_	d1nf6o_	d1nfna_	d1nfoa_
d1nfvd_	d1nfvf_	d1nfvi_	d1nfvl_	d1nfvm_	d1nfvn_	d1nfvo_	d1nh5a_
d1nji1_	d1njic2	d1njir_	d1nloc_	d1nlpc_	d1nm7a_	d1nmra_	d1nnoa1
d1nnqa1	d1nnqb1	d1noea_	d1noga_	d1nora_	d1nppa2	d1nppb2	d1nppc2
d1nq4a_	d1nsha_	d1nshb_	d1ntkc1	d1ntmc1	d1ntxa_	d1ntzc1	d1nu1f_
d1nu9c2	d1nu9f2	d1nuba3	d1nubb3	d1nuia2	d1nxba_	d1ny9a_	d1nyfa_
d1nyrb2	d1nyub_	d1nz6a_	d1nz9a_	d1nzba1	d1nzbb1	d1nzbe1	d1nzbf1

Continued on Next Page...

Domain Identifiers

d1o0wa2	d1o0wb2	d1o3tb1	d1o3ua_	d1o50a2	d1o57a1	d1o57d1	d1o5dt2
d1o7lb1	d1o84a_	d1o84b_	d1o8ca2	d1o8cb2	d1o8cc2	d1o8cd2	d1o90a2
d1o92a1	d1o92a2	d1o92b2	d1o93a1	d1o93a2	d1o93b2	d1o9ra_	d1o9rb_
d1o9rd_	d1o9re_	d1o9rf_	d1o9ta1	d1o9ta2	d1o9tb2	d1o9ya_	d1o9yb_
d1o9yd_	d1oaym_	d1oayo_	d1ocod_	d1ocoe_	d1ocoq_	d1ocor_	d1ocre_
d1ocze_	d1oczr_	d1oeba_	d1oebb_	d1oede_	d1of9a_	d1ogad2	d1ohhd1
d1oizb1	d1ojva2	d1ojvb2	d1ojwa2	d1ojwb2	d1ojya2	d1ojyb2	d1ojyc2
d1ok1a2	d1ok1b2	d1ok2a2	d1ok2b2	d1ok3a2	d1ok3b2	d1ok9a2	d1ok9b2
d1on2a1	d1on2b1	d1onja_	d1onva_	d1oo4a_	d1oota_	d1opka1	d1opla1
d1oqcb_	d1oqcc_	d1oqcd_	d1oqdk_	d1oqsa_	d1oqsc_	d1oqse_	d1oqsg_
d1or3a_	d1or5a_	d1or7a2	d1otfa_	d1otfb_	d1otfc_	d1otfd_	d1otfe_
d1ouqa1	d1ouqb1	d1ouqe1	d1ouqf1	d1ov3a2	d1ov3b2	d1ovna1	d1ovnb1
d1ow5a_	d1ow6a_	d1ow6b_	d1ow6c_	d1ow7a_	d1ow7b_	d1ow7c_	d1ow8a_
d1ow8c_	d1ox3a_	d1oxja2	d1oxza_	d1oy6a4	d1oy9a4	d1oyfb_	d1oz4a4
d1oz4c4	d1p0ra_	d1p1hb2	d1p1hd2	d1p22b1	d1p22b2	d1p2pa_	d1p34c_
d1p3ac_	d1p3ag_	d1p3bc_	d1p3bg_	d1p3fc_	d1p3fg_	d1p3gc_	d1p3gg_
d1p3ig_	d1p3kc_	d1p3kg_	d1p3lc_	d1p3lg_	d1p3mc_	d1p3mg_	d1p3oc_
d1p3pc_	d1p3pg_	d1p4wa_	d1p6wa1	d1p7ea_	d1p7fa_	d1p7la2	d1p7lb2
d1p7ld2	d1p92a1	d1pa7a_	d1pava_	d1pb6a1	d1pb6b1	d1pb6d1	d1pbaa_
d1pbya2	d1pcfa_	d1pcxa1	d1pd0a1	d1pd1a1	d1pd7a_	d1perl_	d1perr_
d1pfxl2	d1pg5b2	d1pgaa_	d1pgba_	d1pgra_	d1pgrc_	d1pgre_	d1pgrg_
d1pi2a_	d1pk1a1	d1pk1b1	d1pk1c1	d1pk1d1	d1pk3a1	d1pk3b1	d1pk3c1
d1pkyc1	d1pn5a1	d1pnja_	d1pp9c1	d1praa_	d1prlc_	d1prmc_	d1psea_
d1psra_	d1psrb_	d1psya_	d1pugb_	d1pugc_	d1pugd_	d1pula1	d1puoa1
d1puza_	d1pv3a_	d1pvhb_	d1pvhd_	d1pvma1	d1pvmb1	d1pvpa1	d1pvpb1
d1pvqb1	d1pvra1	d1pvrb1	d1pwta_	d1py2c_	d1py4d_	d1q0da_	d1q0db_
d1q0dd_	d1q0de_	d1q0df_	d1q0dg_	d1q0dh_	d1q0di_	d1q0dj_	d1q0dk_
d1q0fa_	d1q0fb_	d1q0fc_	d1q0fd_	d1q0fe_	d1q0ff_	d1q0fg_	d1q0fh_
d1q0fj_	d1q0fk_	d1q0fl_	d1q0ga_	d1q0gb_	d1q0gc_	d1q0gd_	d1q0ge_
d1q0gg_	d1q0gh_	d1q0gi_	d1q0gj_	d1q0gk_	d1q0gl_	d1q0ka_	d1q0kb_
d1q0kd_	d1q0ke_	d1q0kf_	d1q0kg_	d1q0kh_	d1q0ki_	d1q0kj_	d1q0kk_
d1q0ma_	d1q0mb_	d1q0mc_	d1q0md_	d1q0me_	d1q0mf_	d1q10a_	d1q10b_
d1q3ua1	d1q3ub1	d1q3ue1	d1q3uf1	d1q3va1	d1q3vb1	d1q3ve1	d1q3vf1
d1q46a2	d1q5ta_	d1q5tb_	d1q5va2	d1q5vb2	d1q5vc2	d1q5wb_	d1q6aa_
d1q6ba_	d1q6bb_	d1q79a1	d1q7y1_	d1q7yc2	d1q7yr_	d1q811_	d1q81c2
d1q821_	d1q82c2	d1q861_	d1q86c2	d1q8ca_	d1q8da_	d1q8ka3	d1q8ka4
d1q95k2	d1q95l2	d1qasa1	d1qbja_	d1qbjb_	d1qc7a_	d1qc7b_	d1qcfa1
d1qckb_	d1qdma1	d1qdmb1	d1qdmc1	d1qg7b_	d1qgha_	d1qghb_	d1qghc_
d1qghe_	d1qghf_	d1qghg_	d1qghh_	d1qghi_	d1qghj_	d1qghk_	d1qghl_
d1qgtb_	d1qgtd_	d1qhka_	d1qi3a1	d1qi4a1	d1qi5a1	d1qjta_	d1qk1e1
d1qkdb_	d1qkea_	d1qkla_	d1qkwa_	d1qkxa_	d1qkza_	d1ql3a_	d1ql3b_
d1ql3d_	d1ql4a_	d1ql4b_	d1ql4c_	d1ql4d_	d1qlda_	d1qlsa_	d1qlxa_
d1qm0a_	d1qm1a_	d1qm2a_	d1qm3a_	d1qm4a2	d1qm4b2	d1qm7a_	d1qmca_
d1qn1a_	d1qn2a_	d1qn2c_	d1qnta2	d1qp2a_	d1qp3a_	d1qpka1	d1qpma_
d1qu6a1	d1qu6a2	d1qu7b_	d1quba5	d1qvfa2	d1qvfp_	d1qvfy_	d1qvga2
d1qvh.2	d1qvta2	d1qvtd1	d1qvtd2	d1qvte2	d1qvua2	d1qvud2	d1qvue2
d1qwea_	d1qwfa_	d1qxpa1	d1qyba1	d1qzxa1	d1qzxb1	d1r0bh2	d1r0cb2

Continued on Next Page…

Domain Identifiers

d1r0ob_	d1r2ma_	d1r2mb_	d1r3fa1	d1r4mi_	d1r4mj_	d1r4ml_	d1r4nj_
d1r5na2	d1r5va2	d1r5vc2	d1r5wa2	d1r5wc2	d1r63a_	d1r69a_	d1r6wa2
d1r7ab1	d1r9ti_	d1raab2	d1raad2	d1rabb2	d1rabd2	d1racb2	d1racd2
d1radd2	d1raeb2	d1raed2	d1rafb2	d1rafd2	d1ragb2	d1ragd2	d1rahb2
d1raib2	d1raid2	d1rapa_	d1raqa_	d1rc7a2	d1rc9a2	d1rcpb_	d1rcsb_
d1rg9b2	d1rg9c2	d1rg9d2	d1rgba1	d1rhga_	d1rhgb_	d1rhgc_	d1rhpc_
d1rida1	d1ridb1	d1rioa_	d1riob_	d1rk7a_	d1rk8c_	d1rkwa2	d1rkwb2
d1rkwe2	d1rlpc_	d1rlqc_	d1rmda2	d1rmja_	d1rp8a1	d1rp9a1	d1rpel_
d1rpka1	d1rpwa2	d1rpwb2	d1rpwc2	d1rpwd2	d1rpya_	d1rpyb_	d1rqqc_
d1rr7a_	d1rtya_	d1ruob1	d1rw5a1	d1rwja_	d1rwta_	d1rwtd_	d1rwtf_
d1rwtj_	d1ry1c_	d1ry1d_	d1ryta1	d1rzta2	d1rzte2	d1rzti2	d1rztm2
d1s0yc_	d1s0ye_	d1s0yg_	d1s0yi_	d1s0yk_	d1s1hq_	d1s1ie_	d1s1ii_
d1s30a1	d1s32c_	d1s32g_	d1s3qa1	d1s3qb1	d1s3qc1	d1s3qd1	d1s3qe1
d1s3qg1	d1s3qh1	d1s3qi1	d1s3qj1	d1s3qk1	d1s3ql1	d1s62a1	d1s6da_
d1s6vd_	d1s72a2	d1s72i_	d1s72q_	d1s72z_	d1s7za_	d1sb6a_	d1sbni_
d1sebe2	d1sema_	d1semb_	d1seua1	d1sf0a_	d1sf7a_	d1sg7a1	d1sgva1
d1sh5a1	d1sh5b1	d1sh6a1	d1shfa_	d1shfb_	d1shga_	d1sija1	d1sj7a1
d1sj7c1	d1skta_	d1skud2	d1skye1	d1smxa_	d1sn6a1	d1sn8a_	d1sofa1
d1sofd1	d1sofe1	d1sofg1	d1sofh1	d1sotc1	d1spka_	d1sq3b1	d1sq3c1
d1sq3e1	d1sq3g1	d1sq3h1	d1sq3i1	d1sq3l1	d1sq8a_	d1sqbc1	d1sqpc1
d1sqvc1	d1sqvf1	d1sqxc1	d1sr5.1	d1srma_	d1sroa_	d1srqd_	d1srsa_
d1ss2a_	d1ssfa1	d1ssha1	d1sska_	d1st6a2	d1st6a3	d1st6a7	d1stua_
d1sv0d_	d1sxda_	d1sxjb1	d1sxjc1	d1sxjd1	d1sxje1	d1sxla_	d1syxb1
d1syxf1	d1t07a_	d1t0za_	d1t0zb_	d1t11a1	d1t11b1	d1t1ha_	d1t33a1
d1t3qa1	d1t3qd1	d1t3ta1	d1t4aa_	d1t4ab_	d1t56a2	d1t5wa2	d1t5wd2
d1t6ia_	d1t6ib_	d1t6ic_	d1t6qa_	d1t6qb_	d1t6qc_	d1t6ua_	d1t6ub_
d1t6ud_	d1t6ue_	d1t6uf_	d1t6ug_	d1t6uh_	d1t6ui_	d1t6uj_	d1t6uk_
d1t84a_	d1t9ha1	d1taua1	d1tbaa_	d1tfba1	d1tfsa_	d1tgxa_	d1tgxb_
d1ths.1	d1tjca_	d1tjcb_	d1tjda2	d1tjoa_	d1tjob_	d1tjoc_	d1tjod_
d1tk6b_	d1tk6c_	d1tk6d_	d1tkoa_	d1tkob_	d1tkoc_	d1tkod_	d1tkpa_
d1tkpc_	d1tkpd_	d1tnsa_	d1tnta_	d1tp2b_	d1tp4a_	d1tpkc_	d1tpma_
d1tpxa_	d1tqba_	d1tqca_	d1tqep_	d1tqeq_	d1tqer_	d1tqes_	d1tthb2
d1ttya_	d1tu0b2	d1tu8c1	d1tuca_	d1tuda_	d1tugb2	d1tugd2	d1tuka1
d1tv9a2	d1tvaa1	d1tw9b1	d1tw9c1	d1tw9g1	d1twja_	d1twjb_	d1twjd_
d1txvb3	d1txyb_	d1ty6b3	d1ty7b3	d1tyed3	d1tzya_	d1tzye_	d1u06a1
d1u0lb1	d1u0lc1	d1u2fa_	d1u2ma_	d1u35c1	d1u35g1	d1u3ma_	d1u4ja_
d1u5ka1	d1u5kb1	d1u5la_	d1u5ma_	d1u5ta2	d1u5tb2	d1u5tc1	d1u6gb_
d1u74d_	d1u89a1	d1u8ra1	d1u8rb1	d1u8rc1	d1u8rd1	d1u8rg1	d1u8rh1
d1u8rj1	d1u9la_	d1u9lb_	d1u9na2	d1u9oa2	d1ua5a1	d1ub1a_	d1ubfa2
d1ucva_	d1udla_	d1ue9a_	d1ueba2	d1ueba3	d1uebb2	d1uebb3	d1uega_
d1ufba_	d1ufbb_	d1ufbc_	d1ufbd_	d1uffa_	d1ufza_	d1ug0a_	d1ug7a_
d1ugva_	d1uhca_	d1uhua_	d1ui6a1	d1uj0a_	d1ukwa1	d1ukwb1	d1ul4a_
d1umnb_	d1umnc_	d1umnd_	d1umne_	d1umnf_	d1umng_	d1umnh_	d1umni_
d1umnk_	d1umnl_	d1unge_	d1uoya_	d1upha_	d1usja2	d1ussa_	d1utaa_
d1uuea_	d1uuja_	d1uvba_	d1uvcb_	d1uvhb1	d1uvhc1	d1uvhd1	d1uvqa2
d1uw2a_	d1uw3a_	d1uwva1	d1uwxa1	d1uwxb1	d1uzja2	d1uzjb2	d1uzjc2
d1uzpa2	d1uzqa2	d1v07a_	d1v0ea2	d1v0eb2	d1v0ec2	d1v0ed2	d1v0ee2

Continued on Next Page…

Table 1 – Continued

**Domain Identifiers**							
d1v0fa2	d1v0fb2	d1v0fc2	d1v0fd2	d1v0fe2	d1v0ff2	d1v15a_	d1v1qa_
d1v2za_	d1v38a_	d1v54e_	d1v54r_	d1v55e_	d1v55r_	d1v57a2	d1v57b2
d1v58b2	d1v5na_	d1v6pa_	d1v6pb_	d1v6za1	d1v6zb1	d1v7ba2	d1v7bb2
d1vasa_	d1vb0a_	d1vcba_	d1vcbd_	d1vcbg_	d1vcbj_	d1vdia1	d1veqf_
d1vewb1	d1vewc1	d1vewd1	d1vf5b_	d1vf5d1	d1vfya_	d1vgha_	d1vi0a2
d1viea_	d1vifa_	d1vit.2	d1vjxa_	d1vk5a_	d1vkeb_	d1vkec_	d1vked_
d1vlba1	d1vlga_	d1vlgb_	d1vlgc_	d1vlgd_	d1vlge_	d1vlgf_	d1vlgg_
d1vlsa_	d1vltb_	d1vmaa1	d1vmab1	d1vola1	d1voma1	d1vore_	d1voue_
d1voxm_	d1voye_	d1vp0e_	d1vpia_	d1vq0a2	d1vq0b2	d1vq3a_	d1vq3b_
d1vq3d_	d1vq4a2	d1vq4q1	d1vq4z1	d1vq5a2	d1vq5i1	d1vq5z1	d1vq6a2
d1vq6q1	d1vq6z1	d1vq7a2	d1vq7q1	d1vq7z1	d1vq8a2	d1vq8i1	d1vq9a2
d1vq9z1	d1vqka2	d1vqki1	d1vqla2	d1vqli1	d1vqlz1	d1vqma2	d1vqmi1
d1vqna2	d1vqni1	d1vqnz1	d1vqoa2	d1vqoi1	d1vqoz1	d1vqpa2	d1vqpi1
d1vr4e1	d1vr9a2	d1vr9b2	d1vvda2	d1vvea2	d1vyca_	d1vzga2	d1vzgb2
d1vzhb2	d1vzia2	d1vzib2	d1vzya2	d1vzyb2	d1w07a1	d1w07b1	d1w2by_
d1w7oa_	d1w7xl1	d1w9pa2	d1w9pb2	d1w9ua2	d1w9ub2	d1w9va2	d1w9vb2
d1wama2	d1watb_	d1wb1a1	d1wb1a2	d1wb2a1	d1wb2c2	d1wb3a1	d1wcla1
d1wdka2	d1wdkb2	d1wdla2	d1wdlb2	d1wdma2	d1wdmb2	d1wejf_	d1wema_
d1wf9a1	d1wfka_	d1wfqa_	d1wg8a1	d1wg8b1	d1wgma_	d1wgya_	d1wh6a_
d1wiba_	d1wiza_	d1wj0a_	d1wjaa_	d1wjab_	d1wjba_	d1wjbb_	d1wjoa_
d1wjwa_	d1wlza1	d1wnla1	d1wnlb1	d1wnoa2	d1wnob2	d1wpya1	d1wpyb1
d1wq7b1	d1wqji1	d1wqwa1	d1wqwb1	d1wrsr_	d1wrss_	d1wrtr_	d1ws9a1
d1wsua2	d1wsub1	d1wsub2	d1wsuc1	d1wsud2	d1wtyc_	d1wuda1	d1wudd1
d1wuma1	d1wv8a1	d1wwva1	d1wz0a1	d1x01a1	d1x01b1	d1x27a1	d1x27b1
d1x27d1	d1x27e1	d1x27f1	d1x2ia1	d1x2ib1	d1x2la1	d1x3aa1	d1x47a1
d1x49a1	d1x4sa1	d1x90a_	d1x90b_	d1x91a_	d1xb2b3	d1xb4a2	d1xb4b2
d1xfvo1	d1xfvq1	d1xfvr1	d1xfvt1	d1xgna1	d1xgsa1	d1xhja_	d1xjha_
d1xk4a1	d1xk4b1	d1xk4e1	d1xk4f1	d1xk4i1	d1xk4j1	d1xk4k1	d1xk4l1
d1xmoc2	d1xmoj_	d1xmwa2	d1xn7a_	d1xnia1	d1xnib1	d1xnic1	d1xnid1
d1xnif1	d1xnig1	d1xnih1	d1xnii1	d1xnij1	d1xnqc1	d1xnqj_	d1xnrc1
d1xnsb1	d1xo0a1	d1xo0b1	d1xo2a2	d1xpob2	d1xraa1	d1xraa2	d1xrba2
d1xs8a_	d1xsla1	d1xsla2	d1xsle1	d1xsle2	d1xsli2	d1xslm2	d1xt3a_
d1xvha1	d1xvhb1	d1xw5a1	d1xw5b1	d1xw6a1	d1xw6b1	d1xw6c1	d1xw6d1
d1xwkb1	d1xwkc1	d1xwrb1	d1xwrc1	d1xwrd1	d1xxba_	d1xxhj1	d1xyja_
d1xyua_	d1xywa_	d1xyxa_	d1y02a2	d1y0na_	d1y14b2	d1y1wi_	d1y38b1
d1y6ia1	d1y6xa1	d1y77i_	d1y7qa1	d1y7qb1	d1y8ea1	d1y8tc1	d1y9qa1
d1ycsb2	d1yd8u1	d1yd8v1	d1ydla1	d1yeba_	d1yfha2	d1ygwa_	d1yhqa2
d1yhqq1	d1yi2a2	d1yi2i1	d1yi2q1	d1yi2z1	d1yica_	d1yija2	d1yiji1
d1yijz1	d1yioa1	d1yita2	d1yitq1	d1yitz1	d1yj6a1	d1yj6b1	d1yj6c1
d1yjna2	d1yjnz1	d1yjwa2	d1yjwi1	d1ykca1	d1ykcb1	d1yl3k1	d1yl3k2
d1yl3x1	d1yl4h2	d1yl4m1	d1ynnk1	d1ynte1	d1yoya1	d1ysea1	d1ysya1
d1yuxa1	d1yuxb1	d1yuza1	d1yuzb1	d1yv0i1	d1yv1a1	d1yv1b1	d1yvha2
d1yvwb1	d1yvwc1	d1yvwd1	d1yxba1	d1yxbb1	d1yxbc1	d1yxbd1	d1yxbe1
d1yxbg1	d1yxbh1	d1yyka2	d1yykb2	d1yyob2	d1yywa2	d1yywb2	d1yywc2
d1yz9a2	d1yz9b2	d1z00a1	d1z21a1	d1z2ma1	d1z3eb1	d1z3xa1	d1z3ya1
d1z6rb1	d1z6rc1	d1z6rd1	d1z77a2	d1z92b2	d1z9ea1	d1za1b2	d1za1d2
d1zfia1	d1zfja3	d1zhva2	d1zjka2	d1zjma1	d1zjma2	d1zjna1	d1zjna2

Continued on Next Page…

Domain Identifiers

d1zkgb2	d1zkra1	d1zkrb1	d1zkrb2	d1zl3a1	d1zlac1	d1zlag1	d1zlwm2
d1zlzb1	d1zm3e4	d1zm4c4	d1zm9c4	d1zm9e4	d1zn2a1	d1zpqa1	d1zpqd1
d1zqaa3	d1zqba1	d1zqba3	d1zqca1	d1zqca3	d1zqda1	d1zqda3	d1zqea1
d1zqfa1	d1zqfa3	d1zqga1	d1zqga3	d1zqha1	d1zqia1	d1zqia3	d1zqja1
d1zqka1	d1zqka3	d1zqla1	d1zqla3	d1zqma1	d1zqma3	d1zqna1	d1zqna3
d1zqoa3	d1zqpa1	d1zqpa3	d1zqqa1	d1zqqa3	d1zqra1	d1zqra3	d1zqsa1
d1zqta1	d1zqta3	d1zs4a1	d1zs4c1	d1zs5a1	d1ztda1	d1ztdb1	d1zuga_
d1zvpa1	d1zvpa2	d1zvpb1	d1zvpb2	d1zvpc1	d1zvpc2	d1zvpd1	d1zvpd2
d1zymb1	d1zz8a1	d1zz8b1	d1zz9a1	d1zz9b1	d1zzka1	d256ba_	d256bb_
d2a1jb1	d2a3aa2	d2a3ab2	d2a3ba2	d2a3bb2	d2a3ca2	d2a3cb2	d2a3ea2
d2a3qa1	d2a3qb1	d2a4ha1	d2a6qb1	d2a7la1	d2a7lb1	d2a7wa1	d2a7wb1
d2a7wd1	d2a7we1	d2a7wf1	d2a7wg1	d2a7wh1	d2a7wi1	d2a7wj1	d2a7wk1
d2ab6a1	d2ab6b1	d2ab6c1	d2ab6d1	d2abla1	d2aboa1	d2abxb_	d2acja1
d2acjc1	d2acjd1	d2afpa_	d2airb2	d2airh2	d2alyb5	d2amcb5	d2amga1
d2ap3a1	d2aq0a1	d2aq0b1	d2aroa1	d2aroe1	d2asra_	d2asya1	d2avua1
d2avud1	d2aw4v1	d2aw6a1	d2aw6b1	d2awia1	d2awib1	d2awic1	d2awid1
d2awif1	d2awig1	d2awih1	d2awii1	d2awij1	d2awik1	d2awil1	d2axua1
d2axuc1	d2axud1	d2axue1	d2axuf1	d2axug1	d2axuh1	d2axui1	d2axuj1
d2axul1	d2axva1	d2axvb1	d2axvc1	d2axvd1	d2axza1	d2axzb1	d2axzc1
d2ayga1	d2aygb1	d2b0ja1	d2b2ta2	d2b2tb2	d2b2ua1	d2b2ub1	d2b2va1
d2b2wb1	d2b2ya1	d2b2yb1	d2b48a1	d2b4jc1	d2b4jd1	d2b5aa1	d2b5ab1
d2b5ad1	d2b5id2	d2b63c1	d2b63e2	d2b64j1	d2b64l1	d2b64m1	d2b64o1
d2b66x1	d2b76d1	d2b76p1	d2b7dt2	d2b7ta1	d2b7va1	d2b8ke2	d2b8tb2
d2b97b1	d2b9mj1	d2b9ml1	d2b9mm1	d2b9mo1	d2b9n21	d2b9nx1	d2b9oj1
d2b9om1	d2b9oo1	d2b9p21	d2b9pk1	d2b9px1	d2bbia_	d2bbya_	d2bccc2
d2be9b2	d2bgwa1	d2bgwb1	d2bhia1	d2bhib1	d2bhna1	d2bhnb1	d2bhnc1
d2bjya1	d2bjyb1	d2bjyc1	d2bjyd1	d2bjye1	d2bjyf1	d2bjyg1	d2bjyh1
d2bjyj1	d2bjyk1	d2bjyl1	d2bk6a1	d2bk6b1	d2bk6c1	d2bk6d1	d2bk6e1
d2bkca1	d2bkcb1	d2bkcc1	d2bkcd1	d2bkce1	d2bkcf1	d2bkcg1	d2bkch1
d2bkcj1	d2bkck1	d2bkcl1	d2bkcm1	d2bkcn1	d2bkco1	d2bkcp1	d2bkcq1
d2bkcs1	d2bkct1	d2bkcu1	d2bkcv1	d2bkcx1	d2bkcy1	d2bl7a1	d2bnnb1
d2bo9d1	d2bpca1	d2bpfa1	d2bpfa3	d2bpga1	d2bpgb1	d2btxa_	d2bw1a1
d2bw1c1	d2bw1d1	d2bw1e1	d2bw1f1	d2bw1g1	d2bw1h1	d2bw1i1	d2bw1j1
d2bw1l1	d2bxxb1	d2bzfa1	d2c0ea1	d2c0eb1	d2c0fa1	d2c0fb1	d2c0ga1
d2c12b1	d2c1ya1	d2c2ca_	d2c2la2	d2c2lb2	d2c2lc2	d2c2ld2	d2c2vs1
d2c35b2	d2c35d2	d2c4ja1	d2c4jb1	d2c4jc1	d2c4jd1	d2cazb1	d2caze1
d2cbib1	d2cbja1	d2cbjb1	d2cbla2	d2ccxa_	d2ccya_	d2cdqa3	d2cdqb3
d2cg6a1	d2cg6a2	d2cg7a1	d2cg7a2	d2chna1	d2chnb1	d2choa1	d2chob1
d2ciwa1	d2cixa1	d2ciya1	d2ciza1	d2cj0a1	d2cj1a1	d2cj2a1	d2ckba2
d2ckua2	d2cpna1	d2cpoa1	d2croa_	d2crta_	d2crxa1	d2crxb1	d2csba1
d2csba3	d2csba4	d2csfa1	d2ct7a1	d2ctea1	d2cthb_	d2ctna_	d2ctxa_
d2cv5g1	d2cw0o1	d2cwya1	d2cx8a1	d2cx9a1	d2cx9d1	d2cxda1	d2cxdb1
d2d0na1	d2d2qb3	d2d3ta2	d2d3tb2	d2d4za1	d2d4za2	d2d4zb2	d2d82a1
d2d9ta1	d2dgza1	d2dixa1	d2dkga1	d2dkgb1	d2dl2a2	d2dmya1	d2do3a1
d2dspi1	d2dsri1	d2dtha1	d2dthb1	d2dtia1	d2dtib1	d2dtoa1	d2dtob1
d2dy8a1	d2dyre1	d2dyrr1	d2dyse1	d2dysr1	d2e2je2	d2e2ji1	d2e5lm1
d2e7sk1	d2e7sl1	d2e7sm1	d2eifa2	d2eije1	d2eijr1	d2eike1	d2eikr1

Continued on Next Page…

**Domain Identifiers**							
d2eilr1	d2eine1	d2einr1	d2ejna1	d2ejnb1	d2ejnb2	d2eraa_	d2erja1
d2erje1	d2erje2	d2es2a1	d2es9a1	d2etda1	d2etnc1	d2etsa1	d2euca1
d2eufa2	d2ev6a1	d2ewna1	d2ewna2	d2ewnb1	d2ewnb2	d2exda1	d2eyqb1
d2ez6b2	d2ezaa1	d2ezba1	d2ezca1	d2ezka_	d2ezla_	d2ezxa_	d2ezxb_
d2ezyb_	d2ezza_	d2ezzb_	d2f2ac1	d2f2ca2	d2f2va1	d2f3ma1	d2f3mb1
d2f3md1	d2f3me1	d2f3mf1	d2f43b1	d2f4vl1	d2f52a1	d2f5ca1	d2f5fa1
d2f6md1	d2f8ng1	d2f9bt2	d2fb1a1	d2fb1b1	d2fb1c1	d2fb1d1	d2fb7a1
d2fefb1	d2fefc1	d2ffha2	d2ffhc2	d2fhea1	d2fheb1	d2fi2a1	d2fi2b1
d2fkxa1	d2fkza1	d2fkzc1	d2fkzd1	d2fkze1	d2fkzf1	d2fkzg1	d2fkzh1
d2fl0c1	d2fl0d1	d2fl0f1	d2fl0g1	d2fl0h1	d2flbt2	d2flrt2	d2fmma1
d2fmmc1	d2fmmd1	d2fmpa1	d2fmpa2	d2fmqa1	d2fmsa1	d2fo0a1	d2fowa_
d2fuga1	d2fugc1	d2fugj1	d2fugl1	d2fugs1	d2fugu1	d2fupa1	d2fx0a1
d2fykb1	d2fzfa1	d2g3ba2	d2g3bb2	d2g3ra1	d2g5ca1	d2g5cb1	d2g5ic1
d2gaqa1	d2gb1a_	d2gb8b1	d2gbqa_	d2gdua1	d2gdub1	d2gdva1	d2gdvb1
d2gecb1	d2gena2	d2gepa1	d2gf7a2	d2gf7b2	d2gf7c2	d2gf7d2	d2giwa_
d2gnoa1	d2go5b1	d2gola1	d2gqva1	d2gr8a1	d2gr8b1	d2gr8c1	d2gr8d1
d2gr8f1	d2grla1	d2grlb1	d2grlc1	d2grld1	d2gs4a1	d2gs4b1	d2gsqa1
d2gtua1	d2gtub1	d2gwsa2	d2gwsi2	d2gwsm2	d2gxba1	d2gxbb1	d2gyc32
d2gyqa1	d2gyqb1	d2h0ga2	d2h0gb2	d2h0ha2	d2h0hb2	d2h0ia2	d2h0ib2
d2h3fa1	d2h3ia1	d2h3qa1	d2h3va1	d2h3za1	d2h8ua1	d2h8ub1	d2h9ec1
d2haxa1	d2haxb1	d2hbba1	d2hcka1	d2hckb1	d2hdha1	d2hdhb1	d2hfha_
d2hgim1	d2hgio1	d2hgph2	d2hgpm1	d2hgpo1	d2hgrh2	d2hioa_	d2hmfa2
d2hmfc2	d2hmfd2	d2hoea1	d2hr5a1	d2hr5b1	d2htna1	d2htnc1	d2htnd1
d2htnf1	d2htng1	d2htnh1	d2hv4a1	d2hyfa1	d2hyfc1	d2hygd1	d2i10a1
d2i10b2	d2i2t11	d2i2tl1	d2i2tu1	d2i2v11	d2i2vl1	d2i2vu1	d2i9ga1
d2iada2	d2iama2	d2iana2	d2ianf2	d2iank2	d2ianp2	d2ibzf1	d2icwa2
d2id3a2	d2id3b2	d2ig0a1	d2igda_	d2igga_	d2igha_	d2iima1	d2il6a_
d2indc1	d2io3b1	d2ipka2	d2isoa1	d2isoa2	d2ispa1	d2isya1	d2isyb1
d2iszb1	d2iszc1	d2iszd1	d2it0a1	d2it0b1	d2it0c1	d2it0d1	d2iu7a1
d2iu7c1	d2iu7d1	d2iu7e1	d2iu7f1	d2iu7g1	d2iu7h1	d2iu7i1	d2iu7j1
d2iuob1	d2iuoc1	d2iuod1	d2iuoe1	d2iuof1	d2iuog1	d2iuoh1	d2iuoi1
d2iuza2	d2iuzb2	d2j00e2	d2j00l1	d2j01i2	d2j02e2	d2j02l1	d2j03i2
d2j0xa3	d2j0xb3	d2j18a1	d2j19a1	d2j37b1	d2j47a1	d2j5ma1	d2j5oa1
d2ja5e2	d2ja6e2	d2ja7e2	d2ja7q2	d2ja7s2	d2ja8e2	d2ja8g2	d2jdie1
d2ji1b2	d2ji1c2	d2ji1d2	d2ji2a2	d2ji2b2	d2ji2c2	d2ji2d2	d2ji3a2
d2ji3c2	d2ji3d2	d2jiaa_	d2jmga1	d2liga_	d2ligb_	d2lisa_	d2lyna_
d2lynd_	d2msja_	d2mysa1	d2nmqa1	d2np3a2	d2np3b1	d2np3b2	d2np5a2
d2np5c2	d2np5d2	d2nrpa1	d2nuza1	d2nvuj1	d2nvyc1	d2nyca1	d2nzdc1
d2o16a2	d2o39c1	d2o39d1	d2o6vb1	d2o6vf1	d2oaua2	d2oaub2	d2oauc2
d2oaue2	d2oauf2	d2oaug2	d2odga1	d2odgb1	d2oeda1	d2ofja2	d2ofjb2
d2ofjd2	d2ojea2	d2ojee2	d2ok5a2	d2ok5a3	d2ok5a4	d2opoa1	d2opob1
d2opod1	d2or1l_	d2or1r_	d2orca_	d2orla1	d2osna1	d2otja2	d2otji1
d2otjz1	d2otla2	d2otli1	d2otlq1	d2ovpa1	d2ovpa2	d2ovqa1	d2ovqa2
d2ovra2	d2ow8d1	d2ow8k1	d2ow8m1	d2ow8n1	d2ow8q1	d2ow8u1	d2p66a1
d2p6aa1	d2p7cb1	d2pcbb_	d2pccb_	d2pccd_	d2pfna2	d2pfpa2	d2pfqa1
d2pheb1	d2plpa1	d2pnaa_	d2pnba_	d2pnga1	d2pnia_	d2proa1	d2prob1
d2ptla_	d2q3ta1	d2r63a_	d2seba2	d2seci_	d2sema_	d2semb_	d2srca1

Continued on Next Page…

Table 1 – Continued

Domain Identifiers							
d2uu9c1	d2uu9e2	d2uuae2	d2uuaj1	d2uube2	d2uuce2	d2uwma2	d2uwma3
d2uwmb3	d2uxce2	d2vgha_	d2ycca_	d3amea_	d3at1b2	d3at1d2	d3bccc2
d3caoa_	d3cara_	d3crol_	d3cror_	d3crxa1	d3crxb1	d3cyti_	d3cyto_
d3eraa_	d3erab_	d3ezaa1	d3ezba1	d3ezea1	d3gb1a_	d3gbqa_	d3gtua1
d3hada1	d3hadb1	d3hdha1	d3hdhb1	d3hipa_	d3hipb_	d3hipc_	d3lyna_
d3proc1	d3proc2	d3prod2	d3psra_	d3psrb_	d3sdpb2	d3sema_	d3semb_
d4at1d2	d4crxa1	d4crxb1	d4gbqa_	d4gepa1	d4gsta1	d4gstb1	d4gtua1
d4gtuc1	d4gtud1	d4gtue1	d4gtug1	d4hcka_	d4icba_	d4otaa_	d4otab_
d4otad_	d4otae_	d4otaf_	d4otag_	d4otah_	d4otai_	d4otaj_	d4otak_
d4otam_	d4otan_	d4otao_	d4otap_	d4otaq_	d4otar_	d4otba_	d4otbb_
d4otbd_	d4otbe_	d4otbf_	d4otbg_	d4otbh_	d4otbi_	d4otbj_	d4otbk_
d4otca_	d4otcb_	d4otcc_	d4otcd_	d4otce_	d4otcf_	d4otcg_	d4otch_
d4proc1	d4proc2	d4prod2	d5at1d2	d5croa_	d5crob_	d5croo_	d5crxa1
d5cytr_	d5ebxa_	d5fwga1	d5fwgb1	d5gsta1	d5hcka_	d6at1b2	d6at1d2
d6ebxb_	d6gsva1	d6gsvb1	d6gswb1	d6gsxa1	d6gsxb1	d6paxa1	d7at1d2
d7icea1	d7icea3	d7icfa1	d7icfa3	d7icga1	d7icga3	d7icha1	d7icha3
d7icia3	d7icja1	d7icka1	d7icka3	d7icla1	d7icla3	d7icma1	d7icna1
d7icoa1	d7icpa1	d7icpa3	d7icqa1	d7icqa3	d7icra1	d7icra3	d7icsa1
d7icta3	d7icua1	d7icua3	d7icva1	d7icva3	d7msia_	d8amea_	d8gepa1
d8icaa3	d8icba1	d8icba3	d8icca1	d8icca3	d8icea1	d8icfa1	d8icfa3
d8icga3	d8icha1	d8icha3	d8icia1	d8icia3	d8icja1	d8icja3	d8icka1
d8icla1	d8icla3	d8icma1	d8icma3	d8icna1	d8icna3	d8icoa1	d8icoa3
d8icpa3	d8icqa1	d8icqa3	d8icra1	d8icra3	d8icsa1	d8icsa3	d8icta1
d8icua1	d8icua3	d8icva1	d8icva3	d8icwa1	d8icwa3	d8icxa1	d8icxa3
d8icza1	d8kme.1	d9atcb2	d9icaa1	d9icba1	d9icba3	d9icca1	d9icca3
d9icea3	d9icfa1	d9icfa3	d9icga1	d9icga3	d9icha1	d9icha3	d9icia1
d9icja3	d9icka1	d9icka3	d9icla1	d9icla3	d9icma1	d9icma3	d9icna1
d9icoa1	d9icoa3	d9icpa1	d9icpa3	d9icqa1	d9icqa3	d9icra1	d9icra3
d9icsa3	d9icta1	d9icta3	d9icua1	d9icua3	d9icva1	d9icva3	d9icwa1
